# Asymmetric Single‐Atom Catalysts Govern Diffusible and Non‐Diffusible Non‐Radical Pathways for Selective Degradation of Antibiotic Resistance Genes

**DOI:** 10.1002/advs.76781

**Published:** 2026-07-29

**Authors:** Chen Gao, Jun Sun, Liang Zhang, Litao Lin, Zongfeng Wan, Bingcai Pan, Sheng Tang

**Affiliations:** ^1^ School of Environmental and Chemical Engineering Jiangsu University of Science and Technology Zhenjiang People's Republic of China; ^2^ Department of Civil and Environmental Engineering Carnegie Mellon University Pittsburgh Pennsylvania United States; ^3^ State Key Laboratory of Water Pollution Control and Green Resource Recycling Foundation School of Environment Nanjing University Nanjing People's Republic of China

**Keywords:** ARGs degradation, asymmetric single atom, nonradical pathway

## Abstract

To address the global health threat from antibiotic resistance genes (ARGs), non‐radical dominated advanced oxidation processes (AOPs) show promise for ARG degradation. To better understand and develop the process, a comparative study on diffusible and non‐diffusible non‐radical‐driven AOP would be of great significance. Herein, asymmetric M‐NC (M = Co, Fe) single‐atom catalysts were engineered to enable tunable switching between distinct non‐radical pathways via peroxymonosulfate (PMS) activation. The non‐diffusible electron transfer pathway (ETP)‐dominated Fe‐NC/PMS system displayed higher intrinsic reactivity toward guanine (G) but lower efficiency for ARGs degradation. Compared with diffusible ^1^O_2_, non‐diffusible ETP exhibited a limited spatial accessibility to ARGs, which significantly reduces the degradation efficiency of the ETP pathway. In contrast, the diffusible ^1^O_2_‐driven Co‐NC/PMS system selectively oxidized G, achieving irreversible ARG removal with the highest degradation rate constant (2.0 min^−1^) among reported M‐NC/PMS‐based systems. Highlighting the unique advantage of diffusible ^1^O_2_ in ARG elimination. Moreover, this system maintained high ARG removal (>5.4 log copies/mL) under high salinity (3000 mg/L), low temperature (4°C), and over 20 consecutive cycles, demonstrating exceptional stability and environmental resilience. This study clarifies the inherent advantage of diffusible ^1^O_2_ over non‐diffusible ETP in elimination and delineates a clear direction for future development.

## Graphical abstract

Asymmetric single‐atom metal‐nitrogen‐carbon catalysts modulate the non‐radical pathway during peroxymonosulfate (PMS) activation. A fundamental contrast was revealed between diffusible singlet oxygen (^1^O_2_) and non‐diffusible electron‐transfer pathways (ETP) in antibiotic resistance gene (ARG) degradation. Limited ARG accessibility restricts ETP oxidation, whereas diffusible ^1^O_2_ enables selective guanine oxidation, irreversible ARG inactivation, and robust performance under harsh conditions.

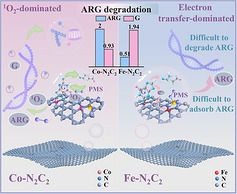

## 1 Introduction

Antibiotic resistance genes (ARGs), which readily undergo horizontal transfer through mobile genetic elements such as plasmids and bacteriophages, have emerged as pervasive environmental contaminants that significantly accelerate the spread of antimicrobial resistance [1, 2, 3]. Notably, ARGs are ubiquitously enriched in pharmaceutical effluents, hospital wastewater, and aquaculture wastewater. These real‐world aquatic environments are typically characterized by high salinity, low operating temperatures, and continuous‐flow conditions, imposing far more stringent constraints on the efficient and stable removal of ARGs [4]. Consequently, the development of ARGs abatement technologies that simultaneously offer high efficiency, strong environmental adaptability, and long‐term operational stability is urgently needed to safeguard public health and aquatic environmental security. Peroxymonosulfate (PMS)‐based advanced oxidation processes (AOPs) have been widely investigated for the effective degradation of ARGs [5, 6], primarily through highly reactive radicals that non‐selectively oxidize nucleobases and disrupt deoxyribonucleic acid (DNA) structures [7, 8, 9]. While such radical‐based pathways can be highly effective under ideal conditions, their performance deteriorates markedly in real water matrices such as low‐temperature environments and in the presence of coexisting ions (e.g., Cl^−^, SO_4_^2−^) [10]. In contrast, non‐radical oxidation pathways have recently attracted increasing attention due to their intrinsic molecular selectivity governed by nucleobase electronic structure matching and prolonged lifetime, which together enable more targeted and less matrix‐sensitive oxidation behavior compared with radical‐based processes [11, 12]. However, despite this promise, research on non‐radical ARG degradation remains extremely limited.

The macromolecular nature and structural complexity of ARGs render their degradation highly dependent on the spatial interaction mode of non‐radical. Generally, non‐radical degradation includes diffusible and non‐diffusible modes, but a systematic comparison between the two modes is still missing. Diffusible species such as singlet oxygen (^1^O_2_) migrate freely in the aqueous phase and enable remote, non‐contact oxidation, conferring intrinsic advantages in degrading bulky macromolecules with complex conformations. In contrast, non‐diffusible non‐radical pathways, particularly electron‐transfer processes (ETP), are confined to the catalyst surface and governed by interfacial electron exchange. While the surface‐controlled nature of ETP may enhance electron utilization efficiency and reaction selectivity, its effectiveness toward macromolecular ARGs can be limited by steric accessibility [13]. Therefore, clarifying the mechanistic distinctions and functional boundaries between diffusible ^1^O_2_ and non‐diffusible ETP is essential for advancing non‐radical oxidation chemistry and rationally designing ARG‐targeted AOP systems for complex water matrices. To date, only a few studies have been reported, diffusible ^1^O_2_‐driven AOPs for ARG degradation [14, 15]. As a result, our current understanding of non‐radical ARG degradation is restricted to a single mechanistic paradigm, with research on non‐diffusible reactive oxygen species (ROS) still entirely unexplored. This knowledge gap significantly limits our ability to tailor oxidation pathways according to the spatial and structural characteristics of ARGs.

The tunable coordination and electronic structure of single‐atom catalysts (SACs) enable directional control over non‐radical generation, providing a rational platform to design selective oxidation pathways [16, 17]. Currently, symmetric Metal‐N_4_‐C SACs with relatively high metal loadings (18.4 wt%) have been applied for ARGs degradation via a ^1^O_2_‐dominated pathway [18]. Building upon this, asymmetric SACs, such as heteroatom‐doped planar structures, offer additional opportunities for enhancing catalytic performance by inducing electron redistribution at the metal center, modulating the occupancy of d_x_^2–^d_y_^2^, d_xy_ and d_z_^2^ orbitals, and enhancing the internal electric field, thereby promoting directional electron transfer. These properties suggest that asymmetric SACs could offer more effective non‐radical pathways [19, 20]. Against this backdrop, it is both scientifically compelling and practically important to develop asymmetric SACs that enable access to multiple non‐radical pathways and to clarify their distinct roles in ARG degradation. Accordingly, to better understand and develop non‐radical dominated AOPs, this study aims to (i) develop asymmetric SACs for effective and selective remediation of ARGs, and (ii) systematically elucidate and compare the mechanistic contributions of diffusible non‐radical pathways (^1^O_2_) and non‐diffusible non‐radical pathways (ETP) in governing ARG degradation.

In this study, a series of asymmetric SACs (Co‐NC, Fe‐NC, Ni‐NC and Cu‐NC) were designed to disentangle the selectivity and mechanistic differences among non‐radical pathways in ARG degradation. By tuning the coordination environment of isolated metal sites, Fe‐NC and Co‐NC were directed toward the ETP and ^1^O_2_‐dominated pathway, respectively. Despite exhibiting a substantially higher degradation rate to guanine (G), the non‐diffusible ETP‐dominated Fe‐NC system showed markedly lower efficiency for ARG degradation compared with the diffusible ^1^O_2_‐dominated Co‐NC system. Detailed experiments and DFT calculations revealed that the ETP‐based pathway is limited by the restricted accessibility of Fe‐NC active sites to macromolecular ARGs. These findings suggest that future catalyst design should focus on creating ARG‐specific adsorption sites that match the spatial conformation and size of ARGs, potentially overcoming accessibility limitations and enabling more effective ETP‐driven degradation. Notably, asymmetric Co‐NC displayed the highest degradation rate constant among all existing M‐NC/PMS‐based oxidation systems for ARG remediation, thereby demonstrating the unique advantage of diffusible ^1^O_2_ in ARG degradation. Furthermore, continuous‐flow experiments further demonstrated the remarkable stability and environmental resilience of Co‐NC/PMS, which maintained high ARG removal efficiency under challenging conditions such as extremely high salinity, low temperature and remarkable stability for repeated cycles. Collectively, this work elucidates the critical advantage of diffusible ^1^O_2_ over non‐diffusible ETP in achieving efficient ARG degradation, and provides the design guidance for the optimization of ETP‐based oxidation systems to enable high‐efficiency ARG removal.

## 2 Results and Discussion

### 2.1 Synthesis and Characterizations of M‐NC Catalysts

**FIGURE 1:**
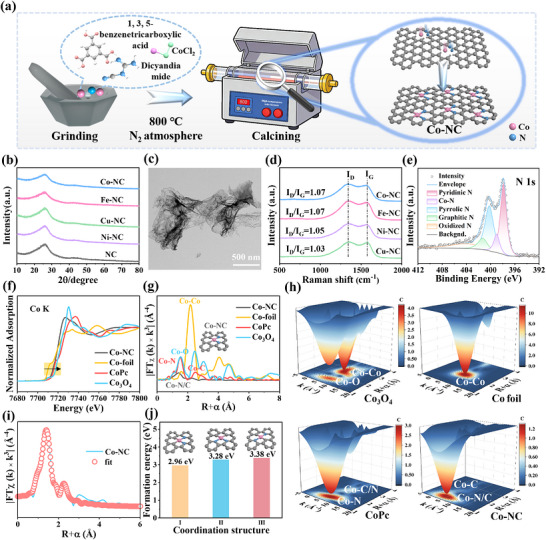
(a) Schematic of SACs synthesis featuring asymmetric coordination. (b) XRD patterns of various SACs. (c) TEM image of Co‐NC. (d) Raman spectra of different single atoms. (e) High‐resolution N 1s XPS spectra of Co‐NC. (f) Normalized Co K‐edge XANES spectra of Co‐NC and reference compounds. (g) Co K‐edge FT‐EXAFS spectra of Co‐NC and references. (h) Co K‐edge EXAFS fitting analysis for Co‐NC. (i) Calculated formation energies of three Co‐NC structural configurations. (j) The WT plot of Co‐NC.

To construct atomically dispersed metal sites, metal salts were intimately mixed with nitrogen‐rich organic precursors to establish uniform metal‐ligand coordination prior to thermal treatment. During high‐temperature pyrolysis, dicyandiamide and melamine condense into a polymeric CN framework that confines M^2+^ species (Co^2+^, Fe^2+^, etc.) and subsequently converts into N‐doped carbon, where strong coordination confinement and high migration barriers suppress metal aggregation. (Figure 1a). X‐ray diffraction (XRD) patterns (Figure 1b) of all M‐NC catalysts only depicted a characteristic peak at 27.7° assigned to graphitic carbon, consistently observed across all samples, while no diffraction signal corresponding to metallic or metal oxide phases was observed. Scanning electron microscope (SEM) and Transmission electron microscopy (TEM) images (Figure 1c; Figure S1) revealed the presence of micrometer‐sized graphene‐like nanosheets, with no observable Co nanoparticles, thereby excluding the aggregation of metallic species. Raman spectra of M‐NC exhibited characteristic D and G bands at ∼1350 cm^−1^ and ∼1580 cm^−1^, respectively, indicating the presence of structural defects and graphitic domains (Figure 1d). The intensity ratio (I_D_/I_G_) of M‐NC was calculated as 1.05‐1.07, indicating a high defect density within the carbon framework that provides abundant active sites while promoting the effective dispersion and stabilization of the metal species.

Besides, X‐ray photoelectron spectroscopy (XPS) was conducted to further elucidate the chemical environment of the metal species. The N 1s XPS spectra of the M‐NC samples exhibited five deconvoluted peaks located at 397.6 eV, 398.8 eV, 399.8 eV, 401.5 eV and 403.8 eV, corresponding to M‐N coordination, pyridinic N, pyrrolic N, graphitic N and oxidized N species, respectively (Figure 1e; Figures S4–S6) [21]. Among them, the M‐N coordination and pyridinic/pyrrolic N species are predominant, which are known to effectively coordinate with transition metal atoms and stabilize atomically dispersed catalytic sites. The nearly identical nitrogen configurations in different M‐NC catalysts reveal that the N‐doping pathway during pyrolysis is fundamentally unaffected by the coordinated metal center. Detailed analysis of O 1s and C 1s XPS spectra indicated the presence of various surface functionalities, including C═O, C─O, OH, C═C, C─N and C═N (Figure S3b,c). These heteroatom‐doped carbon moieties may also contribute to the overall catalytic activity by acting as auxiliary active sites or electronic regulators.

Furthermore, X‐ray absorption spectroscopy (XAS) was further employed to probe the electronic structure and coordination environment of Co atoms in Co‐NC. As shown in the normalized Co K‐edge X‐ray absorption near‐edge structure (XANES) spectra (Figure 1f), the absorption edge of Co‐NC lay between those of Co foil and Co phthalocyanine (CoPc), and was closer to the latter, indicating that Co existed predominantly in the +2‐oxidation state [22]. The Fourier‐transformed k^3^‐weighted extended X‐ray absorption fine structure (EXAFS) spectra (Figure 1g) revealed no Co‐Co scattering peak near 2.18 Å, confirming the absence of metallic Co clusters. Instead, a pronounced peak at ∼1.5 Å was observed, corresponding to Co‐N coordination, which was also supported by wavelet transform (WT) analysis (Figure 1h), further ruling out the presence of Co nanoparticles or sub‐nanoclusters. Notably, a well‐defined pre‐edge peak at ∼7710 eV indicated a low‐symmetry non‐centrosymmetric coordination environment. EXAFS fitting (Figure 1i; Figure S7 and Table S3) yielded coordination numbers of ∼2.3 for Co‐N and ∼1.8 for Co‐C, suggesting a Co‐NC coordination configuration embedded within the carbon matrix. Besides, Co‐NC exhibited a relatively large specific surface area of 220.74 m^2^ g^−1^, indicating a highly developed porous structure (Figure S8). Among the three possible Co‐NC structural motifs proposed (Figure 1j), formation energy calculations indicated that structure I was the most thermodynamically stable and thus most likely to form [23]. Importantly, the optimized CIF‐based structural model I used for EXAFS fitting is in good consistency with the experimental spectra (Figure S9), while complementary TOF‐SIMS fragment analysis further confirms the presence of Co‐N_2_C_2_ coordination signatures (Figure S10), collectively providing multi‐scale evidence for the atomically dispersed Co‐NC configuration with structure I. Therefore, in the following discussion, structure I was designated as the representative model for Co‐N_2_C_2_ coordination, serving as the basis for subsequent investigations.

### 2.2 Selective Catalytic Activity of M‐NC Catalysts toward ARGs and G Removal

**FIGURE 2:**
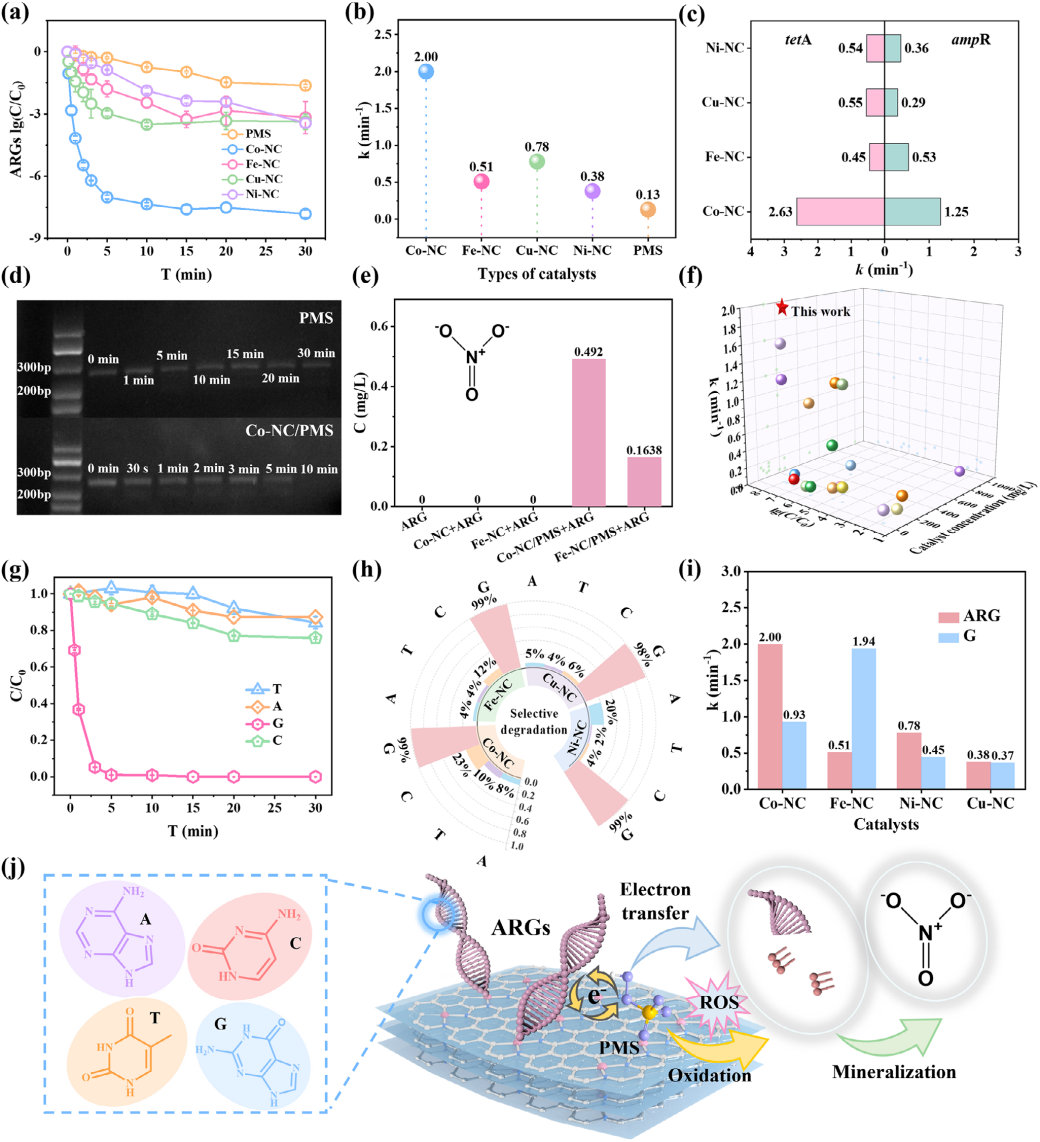
(a) Catalytic performance of different catalysts for *bla*_TEM‐1_. (b) Comparison of degradation and first‐order rate constant of ARGs by Co‐NC, Fe‐NC, Cu‐NC and Ni‐NC. (c) First‐order reaction rate constants of ARGs by different SACs. (d) Gel electrophoresis of *bla*_TEM‐1_. (e) The concentration of NO_3_^−^ after processing *pBR*322 under different reaction systems. (f) Comparisons of recently reported degradation *k* values of ARGs with those in this work. (g) Degradation of different bases by Co‐NC. (h) Selective degradation of A, T, C and G by different SACs. (i) Comparison of *k* values for the degradation of ARGs and G by prepared catalysts. (j) Schematic diagram of ARGs degradation. Experimental conditions: [Catalyst] = 100 mg/L, [PMS] = 0.5 mM, T = 25°C, [ARGs] = 10^12^ copies/mL.

ARGs disseminate through mobile genetic elements, facilitating interspecies horizontal transfer and thereby accelerating multidrug resistance, undermining antibiotic efficacy, and perpetuating resistance within environmental reservoirs. (Figure S11). From a molecular structural perspective, the recalcitrance of ARGs originates from their intrinsic physicochemical properties as functional DNA fragments. ARGs typically exist in highly stable double‐helical supercoiled structures or circular plasmid conformations. Within these configurations, base pairs are stabilized by π–π stacking interactions and extensive hydrogen‐bonding networks, forming a densely packed hydrophobic core. Such compact architectures significantly restrict the accessibility of reactive oxidants to the active sites, thereby markedly limiting reaction efficiency. Accordingly, *bla*_TEM‐1_, a widely distributed plasmid‐borne β‐lactamase gene with a well‐defined DNA sequence and high qPCR amplifiability, was selected as a model ARG to sensitively probe oxidative cleavage and loss of genetic integrity in the M‐NC/PMS system. As shown in Figure S12, in the absence of PMS, the Co‐NC catalyst only showed negligible adsorption of *bla*_TEM‐1_, a trend similarly observed for all other M‐NC catalysts. PMS alone also showed limited degradation capability toward *bla*_TEM‐1_. In contrast, the Co‐NC/PMS system eliminated 7.82 log copies/mL of *bla*_TEM‐1_ within 10 min, with an apparent first‐order rate constant (*k*) of 2.0 min^−1^, approximately 15.4 times higher than PMS alone, underscoring the pivotal role of Co‐NC in PMS activation (Figure 2a). Compared with Co‐NC, Fe‐NC, Ni‐NC and Cu‐NC systems, which achieved relatively lower *bla*_TEM‐1_ removals of 3.36 log copies/mL, 3.80 log copies/mL, and 3.58 log copies/mL, with corresponding *k* values of 0.51 min^−1^, 0.78 min^−1^, and 0.38 min^−1^, respectively (Figure 2b). Except for *bla*_TEM‐1_, the superior catalytic efficiency of Co‐NC extended to different ARGs (Figure 2c; Figure S13), including *amp*R (a regulatory gene that induces β‐lactamase expression and confers resistance to β‐lactam antibiotics) and *tet*A (a tetracycline efflux pump gene that mediates resistance by actively exporting tetracycline from the cell). The Co‐NC catalyst achieved the removal of 7.08 log copies/mL and 8.24 log copies/mL of *amp*R and *tet*A within 30 min, exhibiting *k* values of 1.25 and 2.63 min^−1^. Additional comparative experiments conducted under otherwise identical conditions revealed that both Co‐NC and Fe‐NC exhibited negligible degradation activity toward G and ARGs in H_2_O_2_‐ and peroxydisulfate (PDS)‐based systems (Figure S14), indicating that efficient oxidative transformation occurred selectively in the PMS system and further confirming the oxidant‐dependent activation behavior of the single‐atom catalytic sites. Agarose gel electrophoresis was further employed to trace the structural integrity and fragment size evolution of *bla*_TEM‐1_ during catalytic degradation. As depicted in Figure 2d, the DNA bands corresponding to *bla*_TEM‐1_ progressively faded and ultimately vanished in the Co‐NC/PMS system, signifying extensive cleavage of the DNA backbone and consequent loss of genetic amplifiability. These results were in strong agreement with the structural disruptions revealed by nanopore sequencing (Figure S15), thereby providing complementary evidence of irreversible ARG damage at the molecular level. To further elucidate the oxidative transformation pathways, the formation of nitrate ions (NO_3_^−^), as a representative terminal product of nitrogenous nucleobase oxidation, was quantitatively analyzed by ion chromatography. As shown in Figure 2e, the NO_3_^−^ concentrations reached 0.163 mg L^−1^ and 0.492 mg L^−1^ in the Fe‐NC/PMS and Co‐NC/PMS systems after 30 min, respectively, confirming the conversion of nucleobase‐derived nitrogen into inorganic species. Based on these results, the transformation efficiencies of organic nitrogen in ARGs to inorganic nitrate nitrogen were calculated to be 9.8% for the Fe‐NC/PMS system and 29.4% for the Co‐NC/PMS system. Notably, the higher conversion efficiency observed in the Co‐NC/PMS system is well consistent with its superior performance in ARG degradation, indicating a more effective oxidative conversion of nitrogen‐containing moieties. Although complete mineralization was not achieved under the investigated conditions, the significantly enhanced nitrogen conversion further demonstrates the stronger capability of the Co‐NC/PMS system in driving the oxidative transformation of nitrogen species within ARGs. Taken together, these results clearly demonstrate that *bla*_TEM‐1_ undergoes substantial disruption of its DNA architecture under catalytic oxidation conditions. However, the nucleotide framework is not fully mineralized, indicating that partial structural remnants may persist within the Co‐NC/PMS catalytic system, despite the pronounced degradation of its genetic integrity. Time‐dependent TOC measurements further revealed distinct mineralization behaviors between the two systems (Figure S16), where Co‐NC exhibited a rapid initial TOC removal (∼6.0% within 5 min) followed by a pronounced plateau, whereas Fe‐NC showed a gradual and sustained increase from ∼5.0% to ∼8.0% over 15 min, indicating a higher extent of progressive mineralization and deeper oxidative conversion of G intermediates in the Fe‐NC system.Taken together, these findings clearly demonstrated that *bla*_TEM‐1_ underwent disruption of its DNA architecture, but the nucleotide sequence remained incompletely degraded within the Co‐NC/PMS catalytic system.

Notably, when benchmarked against recently reported catalytic systems for ARG abatement (Figure 2f), the Co‐NC/PMS platform displayed markedly superior degradation kinetics and higher removal efficiency at lower catalyst dosage, underscoring its exceptional promise for practical application in environmental remediation. Besides, various reaction parameters, including the concentration of PMS and catalyst, were systematically tuned to evaluate their effects on the catalytic performance of the Co‐NC/PMS system (Figure S18).

To probe the molecular basis of catalytic differences among M‐NC catalysts, the four nucleobases, A, T, C and G, were employed as model substrates, as disruption of specific bases drives ARG degradation. As shown in Figure 2g, the Co‐NC/PMS system exhibited the highest catalytic activity toward G among the four nucleobases, whereas its oxidation of the other bases was relatively limited. This selective degradation of G was consistently observed in other M‐NC (Figure 2h; Figure S19). These results suggested that each catalyst may preferentially target G sites on the DNA strand, likely due to the lower oxidation potential and higher highest occupied molecular orbital (HOMO) energy of G. Such site‐specific cleavage could trigger fragmentation of ARG molecules. Notably, as shown in Figure 2i, although Co‐NC demonstrated superior overall ARG oxidation efficiency compared with Fe‐NC, its G degradation kinetic (k = 0.93 min^−1^) was more than two‐fold lower than that of Fe‐NC (k = 1.94 min^−1^) (Figure S20). This indicated that, while G oxidation was critical, it was not solely responsible for governing the overall degradation performance of ARGs. The oxidative decomposition of different nucleotide bases within ARGs can lead to the cleavage of the DNA structures (Figure 2j). Differences in degradation outcomes likely arise from ROS types and distribution, ARG and G adsorption, catalyst‐driven downstream oxidation and electron‐transfer efficiency.

### 2.3 Selective ^1^O_2_ Generation and ETP in Fe‐NC and Co‐NC Systems

**FIGURE 3:**
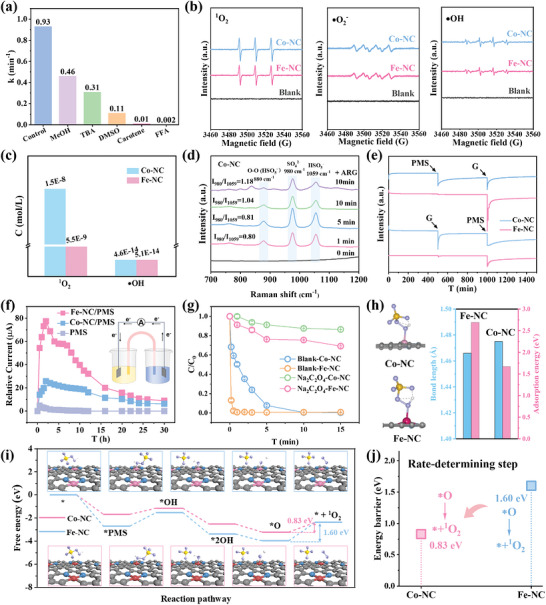
(a) The impact of different quenchers in the Co‐NC system. (b) EPR spectra of •O_2_^−^, •OH/SO_4_^•−^ and ^1^O_2_. (c) Steady state concentration of ROS in Co‐NC and Fe‐NC systems. (d) In situ Raman spectra of Co‐NC under different conditions. (e) Changes in the transient current response curve after adding PMS and G in Co‐NC and Fe‐NC systems. (f) Current variation of the salt‐bridge dual‐cell in the Fe‐NC system. (g) Effect of Na_2_C_2_O_4_ on the degradation of G in Fe‐NC/PMS and Co‐NC/PMS systems. (h) Optimized configurations of PMS adsorbed on Co‐NC and Fe‐NC and the corresponding O─O bond length and adsorption energy. (i) Comparison of reaction energy barriers in the reaction pathway of PMS activation, forming ^1^O_2_ at Co and Fe sites. (j) The rate‐determining step for ^1^O_2_ generation in the Fe‐NC and Co‐NC system. Experimental conditions: [Catalyst] = 100 mg/L, [PMS] = 0.5 mm, [KSCN] = 10 mm, T = 25°C, [G] = 50 µm.

To elucidate the differences in the selective degradation behavior of ARGs and G between Fe‐NC and Co‐NC catalysts, ROS generation in the Fe‐NC and Co‐NC reaction systems was initially conducted. As shown in Figure 3a and Figure S21, the addition of furfuryl alcohol (FFA), methanol (MeOH), tert‐butyl alcohol (TBA), and dimethyl sulfoxide (DMSO) scavengers exhibited varying degrees of inhibition on the M‐NC catalytic degradation process [24, 25]. Notably, the addition of FFA markedly suppressed pollutant degradation in the Co‐NC/PMS (*k* = 0.002 min^−1^) and Fe‐NC/PMS (*k* = 0.15 min^−1^)) systems, highlighting the dominant role of ^1^O_2_. Considering that FFA rapidly consumes PMS (Figure S22), β‐carotene, a slower PMS quencher [26], was further employed and yielded nearly complete inhibition in the M‐NC system, especially Co‐NC (*k* = 0.01 min^−1^) and Fe‐NC (*k* = 0.17 min^−1^) system, thereby validating ^1^O_2_ as the essential ROS in the M‐NC system. In single‐atom catalytic systems, PMS may also undergo oxygen atom transfer to generate high‐valent metal species (HVMS). However, DMSO can also react rapidly with PMS, leading to apparent inhibition unrelated to ROS quenching (Figure S22). To probe this, the selective oxidation of methyl phenyl sulfoxide (PMSO) to methyl phenyl sulfone (PMSO2^)^ of different M‐NC systems was evaluated (Figure S23), yet the conversion (∼15%) was comparable to the PMS‐only system, suggesting negligible involvement of HVMS in all M‐NC systems. Meanwhile, the slight inhibition by MeOH and TBA in the M‐NC reaction system indicates that radicals contribute only marginally, acting as secondary ROS in G degradation.

Electron paramagnetic resonance (EPR) spectroscopy was further employed to identify ROS. As shown in Figure 3d, characteristic signals of 5,5‐Dimethyl‐1‐pyrroline N‐oxide （DMPO)‐•O_2_^−^ (quartet, 1:1:1:1), DMPO‐•OH, and DMPO‐SO_4_^•−^ adducts were detected, with all M‐NC catalysts markedly enhancing their intensities compared to PMS alone, confirming the involvement of •O_2_^−^, •OH and SO_4_^•−^ in pollutant degradation. In addition, a distinct three‐line signal (1:1:1) corresponding to 2,2,6,6‐Tetramethylpiperidine (TEMP)‐^1^O_2_ was observed in all catalytic systems (Figure 3b; Figure S24), validating the generation of ^1^O_2_. It should be noted that quenching experiments may misrepresent ROS contributions due to side reactions such as PMS consumption or interference with ROS generation, and EPR signal intensity alone cannot be used as a quantitative measure of ROS levels across catalytic systems.

To address potential inaccuracies, steady‐state ROS concentrations were evaluated using kinetic modeling combined with multiple chemical probes (Figure S25) [27, 28, 29]. In the Co‐NC/PMS system, ^1^O_2_ predominated over •OH, with other ROS negligible, a trend also observed for Fe‐NC/PMS (Figure 3c). Notably, ^1^O_2_ levels in Co‐NC/PMS were an order of magnitude higher than in Fe‐NC/PMS, while •OH remained comparable, likely explaining the superior ARGs degradation by Co‐NC. Additional validation was provided by deuterium oxide (D_2_O) isotope substitution experiments, which exploit the prolonged lifetime of ^1^O_2_ in D_2_O (Figure S26). Consistent with the steady‐state ROS quantification results, the catalytic activity of Co‐NC increased significantly following D_2_O substitution, confirming the dominant contribution of ^1^O_2_ in the Co‐NC/PMS system. In contrast, Fe‐NC exhibited no obvious activity enhancement under identical conditions, indicating that although ^1^O_2_ was detectable, it was unlikely to function as the dominant reactive species in Fe‐NC/PMS. Collectively, these results demonstrate that the superior ARG degradation performance of Co‐NC originates primarily from its enhanced capability for ^1^O_2_ generation, whereas oxidation in Fe‐NC/PMS is likely governed by alternative non‐radical pathways. In‐situ Raman spectroscopy of Co‐NC further revealed that the I_980_/I_1059_ ratio gradually enhanced during the reaction process, indicating the generation of ^1^O_2_ via PMS cleavage (Figure 3d). Moreover, the introduction of ARGs significantly increased the I_980_/I_1059_ ratio in Co‐NC/PMS systems, suggesting that Co‐NC can efficiently degrade ARGs via PMS activation.

However, despite higher ^1^O_2_, G degradation in Co‐NC/PMS was not superior to Fe‐NC/PMS, indicating that additional oxidation pathways contribute to G breakdown in the Fe‐NC system. Subsequently, ETP was explored, in which single‐atom sites can coordinate with PMS to form activated complexes (PMS^*^) and then interact with pollutants to accelerate degradation. Upon PMS addition, the Fe‐NC system exhibits a pronounced enhancement in transient current response together with a clear shift in open‐circuit potential (Figure 3e; Figure S27), both of which are significantly stronger than those observed in the Co‐NC system, indicating more efficient interfacial electron transfer and electronic reconstruction at the Fe‐NC surface. These electrochemical signatures are consistent with an ETP process in which PMS is activated on Fe‐NC to form reactive PMS* species that mediate interfacial electron transfer between the catalyst and the pollutant.

Moreover, current‐time (I‐T) responses highlighted different PMS activation pathways: Co‐NC generates ^1^O_2_ via direct electron transfer, whereas Fe‐NC acts mainly as an electron‐transfer bridge, operative only when both G and PMS coexist. Salt‐bridge dual‐cell electrochemical measurements reveal that Fe‐NC exhibits a pronounced increase in transient current response upon PMS addition, whereas Co‐NC and PMS systems show only a much weaker current enhancement under identical conditions. These results indicate that Fe‐NC enables significantly more efficient PMS activation through interfacial electron transfer, consistent with the formation of strongly coupled PMS^*^ species and a dominant ETP pathway (Figure 3f). Comparison of PMS consumption rates showed that G markedly accelerated PMS activation in Fe‐NC systems, confirming that G promotes interaction between PMS and catalytic sites (Figure S28). The accelerated PMS consumption rate observed in the Fe‐NC catalyst further confirmed that, in the presence of G, ETP enabled more efficient PMS cleavage, consistent with the reaction kinetics of G degradation. Besides, under high‐concentration PMS pre‐equilibration conditions, PMS consumption in the Fe‐NC system first reaches a quasi‐steady interfacial regime, followed by a pronounced acceleration upon G addition, with further stepwise enhancement observed upon sequential G dosing (Figure S29). This feedback‐accelerated PMS depletion cannot be rationalized by adsorption‐controlled or radical‐only pathways, but is consistent with an ETP‐mediated mechanism in which Fe‐NC enables coupled electron transfer between G oxidation and PMS activation, leading to mutual reinforcement of reaction kinetics. These phenomena can be attributed to PMS serving as the terminal electron acceptor in the ETP pathway, where rapid charge transfer among G, the catalyst, and PMS drives its accelerated decomposition. Similar electrochemical impedance spectroscopy across M‐NC catalysts ruled out conductivity effects (Figure S31), indicating that differences in activation mechanisms stem primarily from variations in the electronic structures of the metal centers.

Previous studies have reported that non‐metallic sites such as pyridinic N, pyrrolic N and graphitic N may also interact with PMS to promote pollutant degradation [30, 31]. To evaluate their role, a correlation analysis was conducted between the content of functional groups (C═O, pyridinic N, pyrrolic N and graphitic N) and *k* of G degradation across various SACs (Figure S32). The analysis revealed no significant correlation between non‐metallic site content and reaction kinetics, thereby excluding their dominant role in PMS activation. To further elucidate the role of metal centers in the catalytic process, sodium oxalate (Na_2_C_2_O_4_) was employed as a selective probe to block single‐atom metal sites, replacing potassium thiocyanate (KSCN) to avoid its non‐specific consumption of PMS. In contrast to KSCN, which induces rapid PMS depletion and thereby interferes with the intrinsic oxidizing environment, Na_2_C_2_O_4_ exhibits negligible reactivity toward PMS, with no appreciable change in PMS concentration observed within 15 min, ensuring a more reliable evaluation of active site involvement (Figure S33). Upon Na_2_C_2_O_4_ addition, the catalytic efficiency decreased markedly (Figure 3g), indicating effective suppression of the catalytic pathway. Furthermore, the significantly higher performance of M‐NC/PMS systems compared with the NC/PMS system further confirms the indispensable contribution of metal centers. Collectively, these results substantiate that the metal active sites play a pivotal role in PMS activation and subsequent pollutant degradation. These findings reinforced the conclusion that metal centers were crucial active sites for PMS activation and pollutant degradation.

Systematic DFT calculations were performed to compare three representative PMS adsorption configurations on Co‐NC and Fe‐NC surfaces, including terminal O coordination, ‐OSO_3_ oxygen binding, and ‐OH terminal oxygen adsorption modes (Figure S34). The results show that the ‐OH terminal oxygen configuration exhibits relatively weaker adsorption strength, whereas the terminal O and ‐OSO_3_ binding modes present significantly stronger adsorption energies. Considering that excessively strong adsorption may artificially amplify the energy differences associated with subsequent key reaction steps (e.g., PMS activation and intermediate transformation), thereby overestimating reaction barriers and conflicting with the experimentally observed fast reaction kinetics, the ‐OH terminal oxygen configuration was therefore selected as the representative adsorption mode. This choice provides a more realistic description of the interfacial interaction strength and the associated kinetic processes, and avoids overestimation of the endothermic barrier in the ^*^PMS‐to‐^*^OH transformation step.

DFT calculations were performed to elucidate the mechanism underlying the preferential generation of ^1^O_2_ on the metal sites, focusing on the interaction between PMS and M‐NC complexes. Upon approaching the Co‐NC surface, PMS is preferentially adsorbed at the metal center via coordination with the peroxo (O─O) moiety, accompanied by significant charge redistribution that progressively weakens the O─O bond and facilitates its cleavage. Compared with Fe‐NC, the Co‐NC complex exhibits more pronounced O─O bond elongation and moderately weaker adsorption energy, indicating a more balanced adsorption‐activation behavior that favors efficient PMS activation and subsequent release of reactive intermediates (Figure 3h). Following O─O bond cleavage of the adsorbed HSO_5_^−^ species, surface‐bound ^*^OH intermediates are generated at the Co‐NC active site. Subsequently, an additional HSO_5_^−^ molecule is captured at the Co‐NC active site via interfacial coupling interactions, leading to the formation of dual surface‐bound ^*^OH. This intermediate then undergoes stepwise deprotonation, ultimately yielding ^1^O_2_ as the final reactive oxidizing species (Figure 3i). Within this pathway, the rate‐determining step in the Co‐NC/PMS system is identified as the coupling–deprotonation process associated with the transformation of surface oxygenated intermediates toward ^1^O_2_ formation, with a calculated energy barrier of 0.83 eV. In contrast, for the Fe‐NC/PMS system, the conversion of ^*^O intermediates for ^1^O_2_ formation remains the primary kinetic bottleneck (1.6 eV). Notably, the lower barrier observed for Co‐NC relative to Fe‐NC is consistent with its more favorable thermodynamic and kinetic propensity for ^1^O_2_ generation (Figure 3j). This mechanistic interpretation aligns well with the experimentally observed higher steady‐state concentration of ^1^O_2_ in the Co‐NC/PMS system, further supporting the validity of the proposed non‐radical activation pathway

Based on the selective generation of ^1^O_2_ and ETP non‐radical pathways by Co‐NC and Fe‐NC, ^1^O_2_, as a freely diffusing reactive oxygen species, can oxidize pollutants beyond the immediate catalyst‐substrate interface. In contrast, the ETP pathway relies on specific adsorption of the pollutant onto the catalyst surface, functioning as an electron‐conducting bridge between electron donors and acceptors. Consequently, in ETP‐dominated systems, the adsorption behavior of supramolecular ARGs at the catalyst interface plays a critical role in their degradation. This mechanistic distinction underpins the divergent behaviors of the two electrophilic non‐radical pathways and is particularly pronounced in the degradation of structurally complex contaminants such as ARGs.

### 2.4 Catalytic Mechanism toward ARGs and G via ^1^O_2_ and ETP Pathways

In order to elucidate the selective adsorption and degradation behavior of ARGs and G by Co‐NC and Fe‐NC catalysts, two key pollutant removal pathways were first considered: (1) The interfacial adsorption‐direct oxidation pathway concentrates pollutants on the catalyst surface, enabling direct electron transfer or short‐range reactions with ROS at active sites to accelerate bond cleavage and molecular transformation [32, 33, 34]. (2) The homogeneous radical oxidation pathway involves surface‐activated peroxides generating ROS that diffuse into the bulk solution and initiate radical chain reactions, driving non‐ or semi‐selective transformations, including bond cleavage, polymerization and ring‐opening [35, 36]. The dominance of either pathway is primarily determined by the nature of interactions between pollutants and the catalyst surface. Considering that ^1^O_2_ has a relatively long lifetime in solution and can freely diffuse to attack different sites of DNA molecules, whereas ETP reactions generally occur within the confined region near the catalyst surface, requiring DNA adsorption or proximity to active centers for electron transfer, pathway selectivity is strongly influenced by adsorption configurations and binding modes.

**FIGURE 4:**
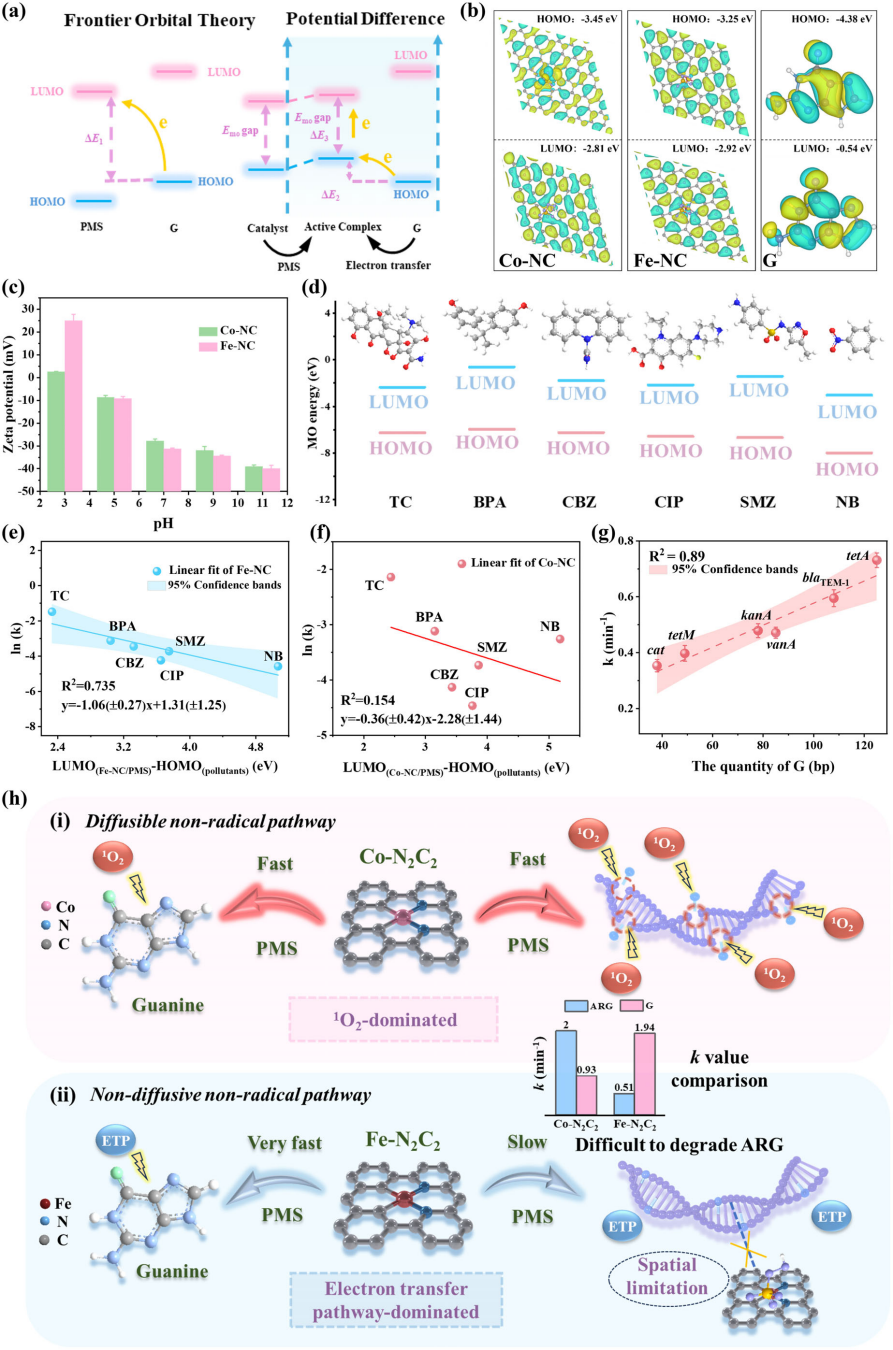
(a) Schematic diagram of the frontier orbital theory for direct oxidation and catalytic oxidation of Fe‐NC. (b) LUMO and HUMO of Co‐PMS^*^, Fe‐PMS^*^ and G. (c) Zeta potentials of Fe‐NC and Co‐NC at different pH values. (d) The Molecular orbital energies of different pollutants. (e, f) Linearity between the electrophilic indexes of different pollutants and their ln (*k*) values in Fe‐NC/PMS and Co‐NC/PMS systems. (g) The correlation between *k* value and G in ARGs. (h) Schematic diagram of Co‐NC and Fe‐NC degradation. Experimental conditions: [Catalyst] = 100 mg/L, [PMS] = 0.5 mm, [Antibiotic] = 100 µm, [ARGs] = 10^13^ copies/mL, T = 25°C.

Notably, Zeta‐potential analysis of Fe‐NC and Co‐NC (Figure 4c) revealed that at pH 3, Fe‐NC exhibited a higher positive surface charge than Co‐NC, whereas at higher pH values, their Zeta‐potentials gradually converged, showing no significant difference. Adsorption experiments for *bla*_TEM‐1_ were conducted at neutral pH, while those for G were performed under acidic conditions. Under these respective conditions, Co‐NC revealed slightly stronger adsorption capacity than Fe‐NC toward *bla*_TEM‐1_, but under acidic conditions, Fe‐NC displayed stronger adsorption affinity for G (Figure S36). This enhanced adsorption is likely attributable to the increased positive surface charge of Fe‐NC at low pH, which facilitates electrostatic interactions or specific binding with deprotonated G molecules. The differential adsorption behavior of ARGs and G on Fe‐NC and Co‐NC suggested that variations in degradation performance may stem from differences in substrate affinity. Specifically, the stronger adsorption of G on Fe‐NC can account for its higher catalytic activity in G degradation, whereas for *bla*_TEM‐1_, where the adsorption capacities of the two catalysts are comparable, overall degradation efficiency is more likely limited by factors beyond adsorption. These observations indicated that small nucleobases are more readily enriched at the catalyst interface, participating in localized interfacial reactions. In contrast, larger ARG molecules, with considerable steric hindrance, tended to undergo degradation primarily via indirect oxidation mediated by solution‐phase ROS. The molecular size, 3D structure and surface charge characteristics of ARGs and nucleobases significantly influenced their affinity for the catalyst surface and directly determined their preferred degradation pathways. Therefore, for ARGs, spatial accessibility emerges as a critical factor governing the reactivity of non‐diffusible non‐radical pathways. Elucidating these mechanistic differences provided a theoretical basis for the rational design of substrate‐specific oxidation strategies, thereby enhancing the selectivity and efficiency of AOPs.

Owing to the reaction kinetics of the ETP, which depend on the electronic coupling strength between the substrate and the metal center, frontier molecular orbital theory was employed to elucidate the PMS‐mediated G degradation mechanism in the Fe‐NC system. In the absence of a catalyst, the direct reaction between G and PMS requires electron transfer from the HOMO of G to the lowest unoccupied molecular orbital (LUMO) of PMS, which involves a large energy gap (ΔE_1_). Upon addition of Fe‐NC, a PMS‐catalyst complex forms, reducing the energy gap (ΔE_3_). The potential difference between the pollutant and the PMS–catalyst complex facilitates electron transfer from the HOMO of G to the HOMO of the complex (ΔE_2_), followed by transfer to its LUMO, thereby promoting PMS decomposition (Figure 4a) [37, 38]. Both ΔE_3_ and ΔE_2_ are significantly smaller than ΔE_1_, rendering the transformation of G to organic radicals thermodynamically more favorable.

To further evaluate the electron transfer capability of the Co‐NC and Fe‐NC catalytic oxidation systems, the molecular orbital energy levels of the M‐NC (Fe and Co)/PMS complexes were calculated. As shown in Figure 4b, although both Fe‐NC/PMS and Co‐NC/PMS complexes meet the energetic requirement for electron transfer from the HOMO of G (−4.38 eV) to their respective LUMO levels, the quantitative energy alignment reveals a subtle difference. The Co‐NC/PMS complex possesses a LUMO at −2.81 eV, whereas that of Fe‐NC/PMS lies lower at −2.92 eV. Correspondingly, the HOMO‐LUMO gap between G and Fe‐NC/PMS (1.46 eV) is indeed smaller than that with Co‐NC/PMS (1.57 eV), which would generally suggest a more favorable ETP in the Fe‐NC/PMS system.

The electron transfer efficiency was assessed by calculating the energy gap between the HOMO of each pollutant and the LUMO of the M‐NC/PMS complex. Accordingly, pollutants with different HOMO and LUMO levels were selected for catalytic degradation studies (Figure 4d). As illustrated in Figure 4e, in the Fe‐NC/PMS system, the LUMO‐HOMO energy gap between the complex and the pollutant correlates significantly with the observed kinetic rate constants. Specifically, pollutants with higher electrophilicity (i.e., lower electron‐donating ability) exhibited slower degradation rates, indicating that a larger energy gap reduces the efficiency of the ETP pathway. In contrast, no similar correlation was observed in the Co‐NC/PMS system Figure 4f, consistent with previous results, suggesting that pollutant degradation by the Co‐NC catalyst is primarily driven by direct PMS activation rather than by an ETP‐dominated pathway. Importantly, adsorption experiments without PMS show that Fe‐NC exhibits only limited and comparable adsorption toward G, and no clear correlation is observed between adsorption capacity and degradation efficiency across different substrates, confirming that adsorption is not a governing factor in the overall degradation process (Figure S37).

Conversely, given the pronounced selectivity of ^1^O_2_ toward G residues within ARGs, it preferentially induces structural disruption, thereby effectively promoting ARG deactivation. To evaluate this effect, a series of ARGs exhibiting distinct G contents and resistance profiles against various antibiotics were selected to assess their degradation behavior in the Co‐NC/PMS system (Figure 4g). Notably, a positive correlation was observed between the degradation rate constants and the G content of the ARG sequences. As an electronically excited singlet state with paired electrons occupying either a single π^*^ orbital or two π^*^ orbitals with antiparallel spin, ^1^O_2_ is highly electrophilic and oxidatively potent, allowing it to selectively attack electron‐rich molecular sites. G, composed of a fused imidazole and pyrimidine ring, possesses an extensively conjugated π‐electron system with high electron density and an elevated HOMO energy level. These features render it a preferential target for ^1^O_2_ attack, thereby becoming the primary cleavage site during ^1^O_2_‐dominated oxidative processes.

The model system was further extended from an isolated G unit to sequence‐resolved dinucleotide motifs (GG, GA, GC and GT), combined with systematic Fukui function analyses (f^+^, f^−^ and f^0^), to capture the evolution of electronic structure and reactive‐site distribution under more realistic nucleic acid environments (Figure S38 and Tables S4 and S5). Compared with the isolated G unit, the formation of dinucleotide structures induced pronounced migration of the sites exhibiting the maximum f^+^, f^0^ and f^−^ values, which were no longer consistently localized on G but instead displayed strong sequence‐dependent electronic redistribution characteristics, indicating that inter‐base π‐π interactions and backbone electronic coupling jointly regulate local electron‐accepting ability, radical susceptibility, and electron‐donating capability. Notably, the sites associated with the maximum f^−^ values also underwent substantial reconstruction across different dinucleotide motifs, with the most pronounced variation observed in the GA structure, suggesting that the introduction of adjacent adenine effectively perturbs the original electron‐enriched state of G and promotes the emergence of alternative preferential oxidation centers. These results demonstrate that oxidative susceptibility in nucleic acids is not solely determined by the intrinsic electronic properties of individual bases but is cooperatively governed by sequence‐dependent electronic delocalization and charge polarization effects. Furthermore, this disparity fundamentally originates from a transition in the reaction system from single‐site localized electronic control to a multi‐base coupled electronic framework. In isolated G, oxidative behavior is primarily governed by the intrinsic HOMO energy level and localized electron density of a single nucleobase; as a result, both ^1^O_2_ and ETP exhibit highly G‐centered attack behavior. However, in realistic ARG or long‐chain DNA systems, inter‐base π–π stacking interactions and backbone charge coupling significantly enhance electron delocalization, leading to a reconstruction of local electronic hotspots and the emergence of sequence‐dependent spatial distribution patterns, thereby weakening the absolute dominance of a single “guanine‐centered” reactive site. Within this context, ^1^O_2_, owing to its diffusible nature and relatively mild electrophilic selectivity, preferentially engages in energy‐transfer‐driven reactions within electron‐enriched regions in a less site‐restricted manner. In contrast, the ETP process proceeds via direct interfacial electron abstraction between the catalyst and nucleic acid, manifesting in multi‐base systems as competitive oxidation driven by cross‐base electron redistribution, which is more strongly governed by interfacial adsorption behavior. This transition from “single‐site recognition” to a “networked electronic response” fundamentally accounts for the observed discrepancy between single‐base models and the behavior of realistic ARG systems.

These results demonstrate that ^1^O_2_‐mediated AOPs effectively induce functional inactivation of ARGs, thereby eliminating their mobility and associated ecological and health risks. Such biological deactivation is primarily attributed to the selective oxidative cleavage of G by ^1^O_2_. Although ETP‐dominated AOPs exhibit superior catalytic activity toward G oxidation, their effectiveness for ARG degradation is intrinsically limited by the supramolecular size and structural complexity of ARGs, which hinder efficient access to the catalyst surface (Figure 4h). This contrast highlights the distinct advantage of diffusible non‐radical species, particularly ^1^O_2_, in overcoming spatial accessibility constraints and achieving efficient ARG degradation.

### 2.5 Application Potential Assessments of Co‐NC Systems

**FIGURE 5:**
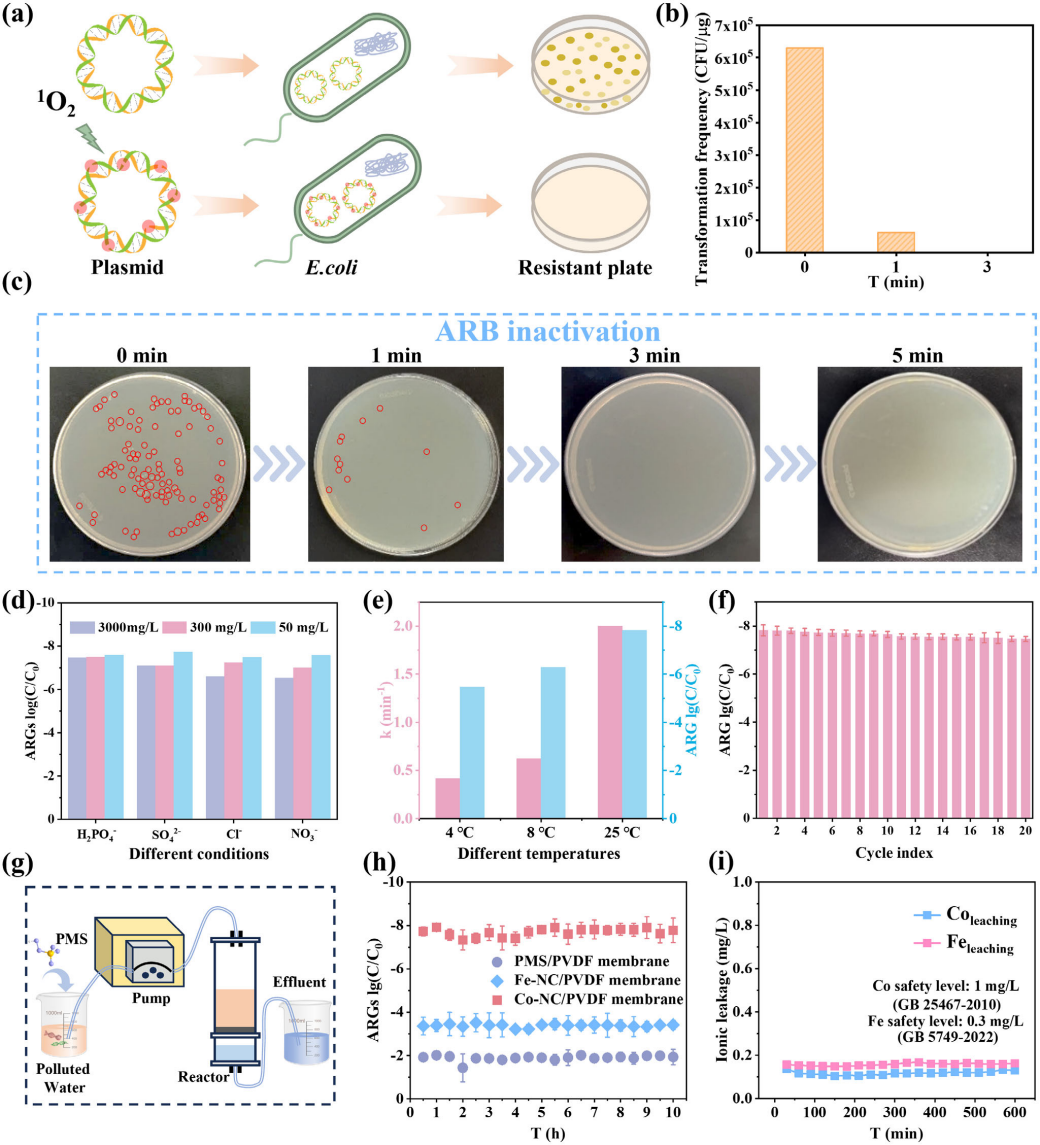
(a) Schematic diagram of plasmid transformation. (b) Transformation frequency of plasmids degraded by the Co‐NC system. (c) Survival status of bacteria after introducing a degraded plasmid into bacteria. (d) The degradation of *bla*_TEM‐1_ by the Co‐NC/PMS system after 30 min of reaction at different ion concentrations. (e) The degradation of *bla*_TEM‐1_ by the Co‐NC/PMS system after 30 min of reaction at different temperatures. (f) Cycle stability of Co‐NC. (g) Schematic diagram of a continuous flow device. (h) Degradation of ARG by different systems during the continuous flow process. (i) Leaching concentration of metal ions in continuous flow processes. Experimental conditions: [Catalyst] = 100 mg/L, [PMS] = 0.5 mm, [Antibiotic] = 100 µm, [ARGs] = 10^12^ copies/mL, T = 25°C.

Following this, to further investigate whether ARGs degraded by ^1^O_2_ retain the potential to induce antibioticresistant bacteria (ARB) through horizontal or vertical gene transfer, extracellular plasmids collected from the Co‐NC/PMS system at different reaction times were transformed into competent bacterial cells. As illustrated in Figure 5a–c, intact extracellular plasmids readily induced ARB formation upon transformation, enabling subsequent growth in antibiotic‐containing media. In contrast, plasmids extracted after 3 min of reaction within the Co‐NC/PMS system failed to produce any observable ARB colonies, and similar results were observed for those reacted for 5 min. Moreover, although partial plasmid structures remained detectable at early timepoints, the transformation frequency of ARGs declined significantly after 1 min of exposure and was completely abolished at 3 min.

ARGs are often enriched in high‐salinity wastewaters, where elevated ionic strength and complex anionic matrices markedly impair conventional AOPs, thereby rendering the development of salt‐tolerant catalytic systems both scientifically and practically imperative. The catalytic activity of the Co‐NC/PMS system was also evaluated under various water matrix conditions. The presence of common anions (SO_4_^2−^, CO_3_^2−^, HCO_3_^−^, H_2_PO_4_^−^ and multiple ions) exerted only slight inhibitory effects on ARG degradation (Figure 5d; Figure S41). Notably, the Co‐NC/PMS system maintained efficient ARG removal even under highly saline conditions with complex ionic interference and ionic strengths exceeding 3000 mg/L (Figure 5d; Figures S42–S44). Reductions exceeding 6 log copies/mL were achieved within 30 min, demonstrating its exceptional resistance to matrix effects and strong applicability for high‐salinity wastewater treatment. From an application perspective, effective ARG abatement under low‐temperature conditions is also essential for cold‐region wastewater treatment, decentralized systems and emergency scenarios. The ^1^O_2_‐dominated AOPs system achieved an ARG removal efficiency of >5.4 log copies/mL within 30 min, even at 4°C (Figure 5e; Figure S45), demonstrating that the low‐temperature‐operable Co‐NC oxidation system can serve as a robust and energy‐efficient strategy for ARG abatement in cold environments, decentralized wastewater, and emergency scenarios. Furthermore, as primary drivers of ARG propagation, Fe‐NC and Co‐NC exhibited pronounced and selective degradation toward diverse antibiotic pollutants, providing a mechanistic basis for targeted pollutant degradation (Figure S46).

Furthermore, the recyclability of Co‐NC was systematically evaluated (Figure 5f). Co‐NC maintained high catalytic activity toward ARG removal over 20 consecutive cycles, with the final cycle still achieving 7.2 log copies/mL removal of *bla*_TEM‐1_, demonstrating its excellent operational durability. Consistently, XRD and TEM analyses revealed negligible changes in crystal structure and morphology after both single and repeated catalytic runs (Figures S47 and S48), confirming the high structural stability of Co‐NC and its resistance to PMS‐induced oxidative reconstruction. Given the unique advantages of the ^1^O_2_‐dominated oxidation pathway in degrading ARGs, a continuous‐flow system was constructed to further evaluate the practical applicability of the Co‐NC/PMS system. In this design, M‐NC catalysts were immobilized onto a poly (vinylidene fluoride) (PVDF) membrane to assess the sustained ARG removal performance (Figure 5g). As shown in Figure 5h, after 600 min of continuous operation, the Co‐NC/PMS system maintained a high removal efficiency of ARGs. In contrast, the control systems using bare PVDF or Fe‐NC catalysts exhibited negligible or rapidly diminished degradation efficiency, underscoring the superior catalytic durability of Co‐NC. Importantly, the Co‐NC catalyst retained excellent structural integrity during the reaction. The concentration of leached metal ions in the treated effluent remained far below the limits stipulated in the Chinese water quality standard (GB 25467‐2010), indicating minimal secondary pollution (Figure 5i). To further exclude the potential interference from leached metal ions, the degradation of individual nucleobases was evaluated in homogeneous metal ion/PMS systems (Figure S47). Remarkably, all homogeneous ion‐catalyzed systems exhibited negligible degradation of G. Among them, Cu^2+^, Ni^2+^ and Fe^2+^ systems also showed minimal removal of the other nucleobases, whereas Co^2+^ was able to efficiently degrade A, T and C. This behavior was in stark contrast to the heterogeneous Co‐NC catalytic system.

In the degradation processes of G by PMS activated via SACs, two distinct oxidative pathways can be identified (Figures S50 and S51). In the ETP, G underwent direct electron abstraction at the catalytic site, generating organic radical intermediates, which led to rapid but relatively non‐selective oxidation and the formation of multiple degradation products. By contrast, ^1^O_2_ selectively attacked specific positions on the G molecule, such as the electron‐rich hydrogen or nitrogen atoms. This results in a more controlled oxidation process with fewer side products and higher selectivity (Figure S52). Therefore, while the ETP pathway maximized oxidation efficiency, the ^1^O_2_ pathway ensures targeted modification of the G structure. Collectively, these findings confirm that the Co‐NC/PMS system, as a green and efficient Fenton‐like catalyst, holds great promise for the sustainable elimination of ARGs and the mitigation of associated ecological and public health risks.

## 3 Conclusion

This study elucidates how asymmetric transition‐metal SACs regulate non‐radical pathways for ARG degradation. By tailoring coordination environments, Fe‐NC and Co‐NC enable selective activation of surface‐confined ETP and diffusible ^1^O_2_, respectively. Although Fe‐NC exhibits higher intrinsic reactivity toward G, systematic comparison reveals that confined ETPs are inherently limited in ARG degradation due to restricted macromolecular adsorption and interfacial accessibility. In contrast, the diffusible ^1^O_2_‐dominated Co‐NC/PMS system achieves efficient and irreversible ARG inactivation in bulk solution, effectively suppressing plasmid‐mediated resistance transfer, highlighting the advantages of diffusible ^1^O_2_ in the degradation of macromolecular ARGs. Frontier‐orbital analysis and first‐principles calculations clarify how asymmetric electronic modulation governs PMS activation thermodynamics and pathway selectivity. Sustained high activity under high salinity (3000 mg/L), low temperature (4°C), continuous operation and repeated cycling highlights the exceptional stability and environmental resilience of the asymmetric Co‐NC/PMS system. Mechanistically, the superior performance of non‐radical pathways originates from G‐targeted electronic selectivity, interface‐confined reaction kinetics, and long‐lived reactive species, which collectively enable efficient oxidation while conferring strong resistance to matrix interference and wide operational tolerance. Collectively, this work provides a strategy for sustainable ARG mitigation in complex water matrices through diffusible ^1^O_2_‐dominated oxidation systems, and simultaneously offers guidance for the rational design and optimization of non‐diffusible ETP‐driven systems to achieve efficient ARG degradation.

## 4 Experimental Section

### 4.1 Synthesis of M‐NC

Iron(II) chloride tetrahydrate (7 mg), cobalt chloride hexahydrate (6 mg), copper chloride dihydrate (3.5 mg) and nickel chloride hexahydrate (6 mg) were each individually mixed with cyanuric acid (100 mg) and dicyandiamide (1.0 g), followed by pyrolysis under a nitrogen atmosphere. The mixtures were heated to 800°C at a rate of 5°C min^−1^ and maintained at this temperature for 3 h. The resulting products are denoted as Fe‐NC, Co‐NC, Cu‐NC, and Ni‐NC, respectively. For comparison, metal‐free carbon nitride nanosheets were prepared under identical conditions.

### 4.2 Degradation and Analytical Methods of Extracellular ARGs (eARGs) and DNA Bases

For the degradation of eARGs, the total reaction volume was 20 mL, with an ARG concentration of 1 × 10^12^ copies/mL (the concentration in pharmaceutical wastewater typically ranges from 10^7^ copies/mL to 10^12^ copies/mL). A catalyst (2 mg) was first added to the reaction system, and after the adsorption equilibrium was reached, 100 µL of PMS (0.1 m) was introduced to initiate the reaction. Samples (200 µL) were collected at 0 min, 10 s, 30 s, 1 min, 2 min, 3 min, 5 min, 10 min, 15 min, 20 min and 30 min, and the reaction in each sample was immediately quenched by the addition of 10 µL of sodium thiosulfate (20 g/L). Different amplicons (Table S7) were designed for qPCR quantification of eARGs, including *tet*A and *bla*_TEM‐1_. qPCR analysis was performed using a Tianlong Gentier 96R system, with each sample tested in triplicate. Standard solutions for calibration were prepared by serial ten‐fold dilutions of plasmid DNA. All reactions were carried out in a final volume of 20 µL, consisting of 5 µL of template DNA, 0.8 µL of each primer, 3.4 µL of nuclease‐free water, and 10 µL of HPOGreen qPCR Master Mix. The thermal cycling program comprised an initial denaturation at 95°C for 1 min, followed by 40 cycles of denaturation at 95°C for 10 s, annealing at 60°C for 30 s, and extension at 60°C for 30 s. Melting curve analysis was subsequently performed to verify amplification specificity. The linearity (R^2^) of standard curves was greater than 0.9, and the average amplification efficiencies are summarized in Table S7.

Stock solutions of adenine (A), thymine (T), cytosine (C) and G (2 mm) were prepared by dissolving A, T and C in ultrapure water, while G was dissolved in 0.48 mm hydrochloric acid solution. Additionally, a PMS stock solution (0.1 m) was prepared for subsequent experiments. The degradation of A, T, C and G was analyzed using high‐performance liquid chromatography (HPLC; Agilent 1260 Infinity II Binary LC System, Germany), with each sample filtered through a 0.22 µm membrane prior to analysis.

### 4.3 Supplementary Experimental and Computational Details

The Supporting Information includes details on Chemicals, Characterization methods, Computational details for X‐ray Absorption Near‐Edge Structure (XANES) simulations, preparation of sensory bacteria and plasmid transformation procedures, normalized kinetic analysis for cross‐system comparison, quantitative analysis of steady‐state ROS concentrations, and ARGs sequences.

## Author Contributions

**Chen Gao**: investigation, validation, methodology, software, data curation, visualization, Writing – original draft. **Liang Zhang**: supervision, writing – review and editing, funding acquisition, visualization, validation, methodology, investigation. **Zongfeng Wan**: software, validation, methodology. **Bingcai Pan**: supervision, visualization, writing – review and editing. **Sheng Tang**: writing – review and editing, supervision, funding acquisition, project administration, resources, formal analysis, data curation. **Litao Lin**: validation, software. **Jun Sun**: conceptualization, methodology, visualization, writing – review and editing, formal analysis.

## Funding

The authors are grateful for the financial support from the National Natural Science Foundation of China (Grant Nos. 22276080, 22574067), the Natural Science Foundation of Jiangsu Province, China (Grant No. BK20241025) and Postgraduate Research & Practice Innovation Program of Jiangsu Province (Grant No. SJCX25_2539)

## Conflicts of Interest

The authors declare no conflicts of interest.

## Supporting information




**Supporting File**: advs76781‐sup‐0001‐SuppMat.docx.

## Data Availability

The data that support the findings of this study are available from the corresponding author upon reasonable request.

## References

[1] H. Sun , W. J. Chang , P. C. Xiong , Z. J. Zhou , Q. Tang , and H. Q. Yu , “Unveiling the Impact of Extracellular Polymeric Substances (EPS) on the Conjugative Transfer of Antibiotic Resistance Genes (ARGs),” Environmental Science & Technology 60, no. 1 (2025): 788–799, 10.1021/acs.est.5c11421. 41324333

[2] M. M. Ellabaan , C. Munck , A. Porse , L. Imamovic , and M. O. Sommer , “Forecasting the Dissemination of Antibiotic Resistance Genes Across Bacterial Genomes,” Nature Communications 12, no. 1 (2021): 2435, 10.1038/s41467-021-22757-1. PMC8065159 33893312

[3] Y. Q. Zhang , L. C. Cheng , F. J. Zhao , M. M. Chen , and P. Wang , “Chiral Pesticides Selectively Influence the Dissemination of Antibiotic Resistance Genes: An Overlooked Environmental Risk,” Environmental Science & Technology 59, no. 26 (2025): 13374–13384, 10.1021/acs.est.4c13010. 40455052

[4] Z. H. Chen , X. Q. Huang , Y. J. Zuo , et al., “High‐entropy Perovskite Embedded in Carbon‐based Catalyst Toward Peroxymonosulfate Activation to Degrade Rhodamine B: Performance and Mechanism Insights,” Water Research 282 (2025): 123919, 10.1016/j.watres.2025.123919. 40456201

[5] F. N. Anandraj , T. K. Panda , S. Thangarasu , G. Palanisamy , and K. E. Neerugatti , “Persulfate Salts to Combat Bacterial Resistance in the Environment Through Antibiotic Degradation and Biofilm Disruption,” Water Research 284 (2025): 123941, 10.1016/j.watres.2025.123941. 40532556

[6] F. Li , P. F. Wang , T. Zhang , et al., “Efficient Removal of Antibiotic Resistance Genes Through 4f‐2p‐3d Gradient Orbital Coupling Mediated Fenton‐Like Redox Processes,” Angewandte Chemie International Edition 62, no. 47 (2023): 202313298, 10.1002/anie.202313298. 37795962

[7] C. Y. Nie , Y. H. Hou , F. Y. Liu , et al., “Efficient Peroxymonosulfate Activation by Magnetic MoS_2_@Fe_3_O_4_ for Rapid Degradation of Free DNA Bases and Antibiotic Resistance Genes,” Water Research 239 (2023): 120026, 10.1016/j.watres.2023.120026. 37182307

[8] M. M. Li , P. F. Wang , K. D. Zhang , et al., “Single Cobalt Atoms Anchored on Ti_3_C_2_T_x_ with Dual Reaction Sites for Efficient Adsorption–Degradation of Antibiotic Resistance Genes,” Proceedings of the National Academy of Sciences 120, no. 29 (2023): 2305705120, 10.1073/pnas.2305705120. PMC10629531 37428922

[9] P. Z. Yang , Y. F. Ji , and J. H. Lu , “Transformation of Ammonium to Nitrophenolic Byproducts by Sulfate Radical Oxidation,” Water Research 202 (2021): 117432, 10.1016/j.watres.2021.117432. 34303167

[10] Y. Zhao , L. Yu , C. Y. Song , Z. L. Chen , F. Y. Meng , and M. Song , “Selective Degradation of Electron‐Rich Organic Pollutants Induced by CuO@Biochar: The Key Role of Outer‐Sphere Interaction and Singlet Oxygen,” Environmental Science & Technology 56, no. 15 (2022): 10710–10720, 10.1021/acs.est.2c01759. 35546088

[11] Z. P. Wang , Z. B. Chen , Q. B. Li , et al., “Non‐radical Activation of Peracetic Acid by Powdered Activated Carbon for the Degradation of Sulfamethoxazole,” Environmental Science & Technology 57, no. 28 (2023): 10478–10488, 10.1021/acs.est.3c03370. 37389809

[12] Y. Meng , Y. Q. Liu , C. Wang , et al., “Nanoconfinement Steers Nonradical Pathway Transition in Single Atom Fenton‐Like Catalysis for Improving Oxidant Utilization,” Nature Communications 15, no. 1 (2024): 5314, 10.1038/s41467-024-49605-2. PMC11192908 38906879

[13] L. He , L. Q. Liu , H. J. Zhou , et al., “Mechanistic Insight Into the Selective Singlet Oxygen Production at Cathode via the Activation of Anodic Water Splitting‐generated Oxygen Bubbles,” Water Research 286 (2025): 124193, 10.1016/j.watres.2025.124193. 40683048

[14] Z. Qi , X. F. Wu , Q. Li , et al., “Singlet Oxygen in Environmental Catalysis: Mechanisms, Applications and Future Directions,” Coordination Chemistry Reviews 529 (2025): 216439.

[15] Y. Q. Li , Z. H. Wei , Z. Y. Sun , H. Z. Zhai , S. H. Li , and W. X. Chen , “Sulfur Modified Carbon‐Based Single‐Atom Catalysts for Electrocatalytic Reactions,” Small 20, no. 38 (2024): 2401900, 10.1002/smll.202401900. 38798155

[16] W. T. Zhang , Y. Zhao , W. G. Huang , T. Y. Huang , and B. D. Wu , “Coordination Environment Manipulation of Single Atom Catalysts: Regulation Strategies, Characterization Techniques and Applications,” Coordination Chemistry Reviews 515 (2024): 215952, 10.1016/j.ccr.2024.215952.

[17] P. F. Li , Y. Deng , H. Y. Wang , et al., “Elucidating the Microenvironment Structure‐Activity Relationship of Cu Single‐Site Catalysts via Unsaturated N,O‐Coordination for Singlet Oxygen Production,” Advanced Functional Materials 34, no. 44 (2024): 2407147, 10.1002/adfm.202407147.

[18] Z. Y. Pan , X. H. Jiang , X. Feng , et al., “Controllable Supply–Demand Effect During Superior Fe Single‐Atom Catalyst Synthesis for Targeted Guanine Oxidation of Antibiotic Resistance Genes,” Environmental Science & Technology 59, no. 10 (2025): 5382–5393, 10.1021/acs.est.4c13667. 40045910

[19] P. P. Cao , X. Q. Mu , F. J. Chen , et al., “Breaking Symmetry for Better Catalysis: Insights Into Single‐atom Catalyst Design,” Chemical Society Reviews 54, no. 8 (2025): 3848–3905, 10.1039/D4CS01031K. 40079812

[20] X. H. Kong , Y. F. Li , G. L. Cai , et al., “Asymmetric Coordinated Single‐Atom Catalysts Offering Zero‐Order Sulfur Redox Kinetics for High Performance Li–S Batteries,” Angewandte Chemie International Edition 64, no. 37 (2025): 202510212, 10.1002/anie.202510212. 40696974

[21] Y. Luo , T. Y. Li , H. Z. Zhang , et al., “Endogenous Symbiotic Li_3_N/Cellulose Skin to Extend the Cycle Life of Lithium Anode,” Angewandte Chemie International Edition 60, no. 21 (2021): 11718–11724, 10.1002/anie.202017281. 33751713

[22] R. M. Zhao , L. Xie , R. S. Zhuang , et al., “Interfacial Defect Passivation and Charge Carrier Management for Efficient Perovskite Solar Cells via a Highly Crystalline Small Molecule,” ACS Energy Letters 6, no. 12 (2021): 4209–4219, 10.1021/acsenergylett.1c01898.

[23] Z. S. Zhu , Y. T. Wang , X. G. Duan , et al., “Atomic‐Level Engineered Cobalt Catalysts for Fenton‐Like Reactions: Synergy of Single Atom Metal Sites and Nonmetal‐Bonded Functionalities,” Advanced Materials 36, no. 32 (2024): 2401454, 10.1002/adma.202401454. 38685794

[24] Q. Zhong , Y. Xue , Z. H. Qi , et al., “FeSeS@C Cage‐in‐Cage Superlattices for Peroxymonosulfate Activation: Surface Acidity Regulates Fe Spin State,” Applied Catalysis B: Environment and Energy 360 (2025): 124539.

[25] P. J. Li , D. L. Xu , X. X. Cheng , et al., “Nonradical‐dominated Nanoconfined Iron Single‐atom Catalytic Membrane to Enhance Peroxymonosulfate Activation for Efficient Water Decontamination,” Water Research 285 (2025): 124106, 10.1016/j.watres.2025.124106. 40580834

[26] Q. Z. Fang , H. L. Yang , S. J. Ye , et al., “Generation and Identification of ^1^O_2_ in Catalysts/Peroxymonosulfate Systems for Water Purification,” Water Research 245 (2023): 120614, 10.1016/j.watres.2023.120614. 37717327

[27] Y. Y. Wang , B. Ma , J. Zhao , et al., “Rapid Inactivation of Fungal Spores in Drinking Water by Far‐UVC Photolysis of Free Chlorine,” Environmental Science & Technology 57, no. 51 (2023): 21876–21887, 10.1021/acs.est.3c05703. 37978925

[28] Y. H. Long , Z. H. Cao , W. R. Wu , et al., “Rational Modulation of Fe Single‐atom Electronic Structure in a Fe‐N_2_B_4_ Configuration for Preferential ^1^O_2_ Generation in Fenton‐Like Reactions,” Applied Catalysis B: Environment and Energy 344 (2024): 123643, 10.1016/j.apcatb.2023.123643.

[29] J. Z. Zhen , J. H. Sun , X. W. Xu , et al., “M−N_3_ Configuration on Boron Nitride Boosts Singlet Oxygen Generation via Peroxymonosulfate Activation for Selective Oxidation,” Angewandte Chemie International Edition 63, no. 26 (2024): 202402669, 10.1002/anie.202402669. 38637296

[30] G. J. Qu , P. Jia , S. Tang , et al., “Enhanced Peroxymonosulfate Activation via Heteroatomic Doping Defects of Pyridinic and Pyrrolic N in 2D N‑Doped Carbon Nanosheets for BPA Degradation,” Journal of Hazardous Materials 461 (2024): 132626, 10.1016/j.jhazmat.2023.132626. 37769450

[31] Y. Wang , H. Li , Y. B. Chen , et al., “Electro‐Fenton Degradation of PFOA in Nano‐confined Spaces: The New Degradation Mechanism That Only Cathode Involved,” Applied Catalysis B: Environment and Energy 374 (2025): 125388, 10.1016/j.apcatb.2025.125388.

[32] Y. J. Xu , R. Hou , K. X. Chi , et al., “A Water‐Resistant and Stable Pd‐Co_3_O_4_ Catalytic Interface for Complete Methane Oxidation With Insights on Active Structures and Reaction Pathway,” Chinese Journal of Catalysis 74 (2025): 191–201, 10.1016/S1872-2067(25)64728-0.

[33] Y. H. Peng , Q. M. Zhang , W. Ren , et al., “Thermodynamic and Kinetic Behaviors of Persulfate‐Based Electron‐Transfer Regime in Carbocatalysis,” Environmental Science & Technology 57, no. 47 (2023): 19012–19022, 10.1021/acs.est.3c02675. 37599507

[34] F. Wang , Y. Gao , H. F. Fu , et al., “Almost 100 % Electron Transfer Regime Over Fe−Co Dual‐Atom Catalyst Toward Pollutants Removal: Regulation of Peroxymonosulfate Adsorption Mode,” Applied Catalysis B: Environmental 339 (2023): 123178, 10.1016/j.apcatb.2023.123178.

[35] S. Meng , P. Zhou , Y. M. Sun , et al., “Reducing Agents Enhanced Fenton‐Like Oxidation (Fe(III)/Peroxydisulfate): Substrate Specific Reactivity of Reactive Oxygen Species,” Water Research 218 (2022): 118412, 10.1016/j.watres.2022.118412. 35453031

[36] C. Y. Teng , Z. H. Wang , Z. Xu , H. F. Zhang , and W. Chen , “Cu(II)‐mediated Homogeneous Advanced Oxidation Processes: Characteristics, Identification, and Environmental Applications of Cu(III),” Water Research 284 (2025): 124000, 10.1016/j.watres.2025.124000. 40532557

[37] J. Y. Liu , P. J. Duan , M. X. Li , et al., “Direct Electron Transfer‐driven Nontoxic Oligomeric Deposition of Sulfonamide Antibiotics onto Carbon Materials for In Situ Water Remediation,” Environmental Science & Technology 58, no. 27 (2024): 12155–12166, 10.1021/acs.est.4c05008. 38934735

[38] P. P. Zhang , Y. Y. Yang , X. G. Duan , Y. J. Liu , and S. B. Wang , “Density Functional Theory Calculations for Insight into the Heterocatalyst Reactivity and Mechanism in Persulfate‐Based Advanced Oxidation Reactions,” ACS Catalysis 11, no. 17 (2021): 11129–11159, 10.1021/acscatal.1c03099.

