# Supplementary material for: Asymmetric Single‐Atom Catalysts Govern Diffusible and Non‐Diffusible Non‐Radical Pathways for Selective Degradation of Antibiotic Resistance Genes
Source: Adv Sci (Weinh). 2026 Jul 29:e76781. Online ahead of print. doi: 10.1002/advs.76781 (PMC13418504; doi:10.1002/advs.76781)
Supplement: Supplementary file 1 — Supporting File: advs76781‐sup‐0001‐SuppMat.docx. [file ADVS-9999-e76781-s001.docx]

Supporting Information

Asymmetric Single-Atom Catalysts Govern Diffusible and Non-Diffusible Non-Radical Pathways for Selective Degradation of Antibiotic Resistance Genes

Chen Gao, ^[a], +^ Jun Sun, ^[b], +^ Liang Zhang,^[a],^ * Litao Lin,^[a]^ Zongfeng Wan,^[a]^ Bingcai Pan,^[c]^ Sheng Tang^[a],^ *

[a] C. Gao, Dr. L. Zhang, L. T. Lin, Z. F. Wan, Prof. S. Tang

School of Environmental and Chemical Engineering

Jiangsu University of Science and Technology

Zhenjiang, 212003, P.R. China

E-mail: zhangliang@just.edu.cn (L. Zhang); tangsheng.nju@gmail.com, chmts@just.edu.cn (S. Tang)

[b] Dr. J. Sun

Department of Civil and Environmental Engineering

Carnegie Mellon University

Pittsburgh, Pennsylvania 15213, United States

[c] Prof. B. C. Pan

State Key Laboratory of Water Pollution Control and Green Resource Recycling Foundation

School of Environment

Nanjing University

Nanjing, 210023, P. R. China

[+] Both authors contributed equally to this work.

**Table of Contents**

[**Text S1. Chemicals** 6](#_Toc233198091)

[**Text S2. Characterization** 7](#_Toc233198092)

[**Text S3. Computational Details for X-ray Absorption Near-Edge Structure (XANES) Simulations** 8](#_Toc233198093)

[**Text S4. Preparation of sensory bacteria and plasmid transformation procedure** 9](#_Toc233198094)

[**Text S5. Normalized kinetic analysis for cross-system comparison** 10](#_Toc233198095)

[**Text S6. Quantitative analysis of steady-state concentration of ROSs** 11](#_Toc233198096)

[**Text S7. ARGs sequence** 12](#_Toc233198097)

[**Figure S1.** TEM (a) and SEM (b) images of Co-NC and TEM (c) and SEM (d) images of Fe-NC. 20](#_Toc233198098)

[**Figure S2.** Full XPS spectra of Co-NC, Fe-NC, Cu-NC and Ni-NC. 21](#_Toc233198099)

[**Figure S3.** The high-resolution XPS spectra of the Co-NC: (a) Co 2p, (b) C 1s and (c) O 1s. 22](#_Toc233198100)

[**Figure S4.** The high-resolution XPS spectra of the Fe-NC: (a) Fe 2p, (b) C 1 s, (c) N 1s and (d) O 1s. 23](#_Toc233198101)

[**Figure S5.** The high-resolution XPS spectra of the Cu-NC: (a) Cu 2p, (b) C 1 s, (c) N 1s and (d) O 1s. 24](#_Toc233198102)

[**Figure S6.** The high-resolution XPS spectra of the Ni-NC: (a) Ni 2p, (b) C 1 s, (c) N 1s and (d) O 1s. 25](#_Toc233198103)

[**Figure S7.** The Fourier transformed k3-weight EXAFS curve and curve fit line in k space and R space for (a-b) Co-NC, (c-d) Co_3_O_4_, (e-f) Co-foil, (g-h) Co-Pc. 26](#_Toc233198104)

[**Figure S8.** (a) Nitrogen adsorption-desorption curve and (b) the corresponding pore-size distribution curves of Co-NC. 27](#_Toc233198105)

[**Figure S9.** CIF-based optimized structural model for EXAFS fitting. 28](#_Toc233198106)

[**Figure S10.** TOF-SIMS fragment analysis of Co-NC. 29](#_Toc233198107)

[**Figure S11.** The transfer of ARGs and the sources of ARBs resistance. 30](#_Toc233198108)

[**Figure S12.** The adsorption of ARGs by different single-atom catalyst systems. 31](#_Toc233198109)

[**Figure S13.** Comparison of degradation and first-order rate constant of different ARGs by Co-NC, Fe-NC, Cu-NC and Ni-NC. 32](#_Toc233198110)

[**Figure S14.** Degradation performance of (a) G and (b) ARGs in different activation systems, including Co-NC/PDS, Co-NC/H_2_O_2_, Fe-NC/PDS, and Fe-NC/H_2_O_2_ oxidation systems. 33](#_Toc233198111)

[**Figure S15.** Analysis of nucleic acid fragments after degradation of ARGs by Co-NC at 0 min (a), 1 min (b) and 3 min (c), respectively. 34](#_Toc233198112)

[**Figure S16.** Mineralization performance of G in Co-NC/PMS and Fe-NC/PMS oxidation systems. 35](#_Toc233198113)

[**Figure S17.** Comparison with the normalized activity evaluation parameters of G degradation reported in the literature. 36](#_Toc233198114)

[**Figure S18.** Optimization experiments of Co-NC under different conditions: (a) catalyst concentration, (b) PMS concentration. 37](#_Toc233198115)

[**Figure S19.** Degradation processes and (b) degradation rate of A, T, C and G by Fe-NC (a), Cu-NC (b) and Ni-NC(c)…………………………………………………………… …………………..38](#_Toc233198116)

[**Figure S20.** (a) Comparison of degradation of base G in different systems. (b) Comparison of apparent first-order rate constant of G by different catalysts. 39](#_Toc233198117)

[**Figure S21.** (a) Quenching experiments of the Co-NC/PMS system. (b) The effect of different quenchers on the degradation of G in Fe-NC system and (c) comparison of apparent first-order rate constant. 40](#_Toc233198118)

[**Figure S22.** Consumption of PMS by different quenchers. 41](#_Toc233198119)

[**Figure S23.** (a) Single PMS conversion experiment from PMSO to PMSO_2_. Experimental study on the conversion of PMSO to PMSO_2_ in Co-NC (b), Fe-NC (c), Cu-NC (d), Ni-NC (e) systems. 42](#_Toc233198120)

[**Figure S24.** (a) EPR spectra of the different systems captured by TEMP. (b-c) EPR spectra of the different systems captured by DMPO. 43](#_Toc233198121)

[**Figure S25.** Degradation efficiency of (a) FFA, (b) NB (c) BA and (d) p-CBA in different catalytic systems. 44](#_Toc233198122)

[**Figure S26.** Degradation efficiency of ARGs in Co-NC/PMS (a-b) and Fe-NC/PMS (c-d) systems with D_2_O and H_2_O as the reaction solvents, respectively. 45](#_Toc233198123)

[**Figure S27.** Open-circuit potential spectra of Co-NC and Fe-NC systems. 46](#_Toc233198124)

[**Figure S28.** The change of PMS concentration (a) and the apparent first-order rate constant of PMS consumption (b) in the reaction systems of Fe-NC/PMS/G and Fe-NC/PMS. 47](#_Toc233198125)

[**Figure S29.** PMS consumption process after introducing G at different reaction stages in the Fe-NC system. 48](#_Toc233198126)

[**Figure S30.** Consumption of PMS by different single-atom systems. 49](#_Toc233198127)

[**Figure S31.** Electrochemical impedance spectroscopy of different single-atom catalysts. 50](#_Toc233198128)

[**Figure S32.** The correlation between the first-order rate constant of G degradation in different catalysis system and the proportion of different chemical species. 51](#_Toc233198129)

[**Figure S33.** Consumption of PMS in the presence of Na_2_C_2_O_4_ and potassium thiocyanate KSCN. 52](#_Toc233198130)

[**Figure S34.** Optimized configurations of PMS adsorbed on Co-NC and Fe-NC and the corresponding O-O bond length and adsorption energy. 53](#_Toc233198131)

[**Figure S35.** The intermediate adsorption and reaction structure of Co-NC (a) and Fe-NC (b). 54](#_Toc233198132)

[**Figure S36.** The adsorption of *bla*_TEM-1_ (a) and G (b) by Co-NC and Fe-NC. (c) Adsorption and degradation of G by Co-NC and Fe-NC. 55](#_Toc233198133)

[**Figure S37.** (a) The correlation between adsorption capacity and degradation efficiency in the Fe-NC/PMS system. (b) The correlation between adsorption capacity and degradation efficiency for different pollutants. (c) The correlation between adsorption capacity and removal efficiency for different pollutants. 56](#_Toc233198134)

[**Figure S38.** Fukui function values of GA (a), GG (b), GC (c) and GT (d). 57](#_Toc233198135)

[**Figure S39.** Regeneration of ARG after degradation. 58](#_Toc233198136)

[**Figure S40.** The degradation of *bla*_TEM-1_ by the Co-NC/PMS system after 30 min of reaction at different pH. 59](#_Toc233198137)

[**Figure S41.** Effects of different inorganic anions on degradation of ARGs by Co-NC activated PMS. 60](#_Toc233198138)

[**Figure S42.** The degradation of *bla*_TEM-1_ by the Co-NC/PMS system after 30 min of reaction at different ions concentrations. 61](#_Toc233198139)

[**Figure S43.** The degradation of *bla*_TEM-1_ by the Co-NC/PMS system after 30 min of reaction at different ions concentrations. 62](#_Toc233198140)

[**Figure S44.** The degradation of *bla*_TEM-1_ by the Co-NC/PMS system after 30 min of reaction at the conditions of Ca^2+^, Na^+^, Cl^-^, SO_4_^2-^. 63](#_Toc233198141)

[**Figure S45.** The degradation of *bla*_TEM-1_ by the Co-NC/PMS system after 30 min of reaction at different temperatures. 64](#_Toc233198142)

[**Figure S46.** Degradation of different antibiotics by Co-NC (a) and Fe-NC (b). 65](#_Toc233198143)

[**Figure S47.** XRD patterns of Co-NC after cycling. 66](#_Toc233198144)

[**Figure S48.** TEM images of Co-NC initially (a), after 1 cycle (b) and after 10 cycles (c) and after 20 cycles (d) 67](#_Toc233198145)

[**Figure S49.** (a) Degradation performance of different metal ions to different bases: A, T, G and C. 68](#_Toc233198146)

[**Figure S50.** (a-b) Ultra-high-resolution mass spectra during the G degradation in Co-NC/PMS reaction system. (c) Proposed degradation pathways of G in Co-NC system. 69](#_Toc233198147)

[**Figure S51.** (a-b) Ultra-high-resolution mass spectra during the G degradation in Fe-NC/PMS reaction system. (c) Proposed degradation pathways of G in Fe-NC system. 70](#_Toc233198148)

[**Figure S52.** The relationship between degradation rate constant and ionization potential. 71](#_Toc233198149)

[**Table S1. The proportion of different kinds of C, N, and O in different single atoms catalysts** 72](#_Toc233198150)

[**Table S3. EXAFS fitting parameters at the Co K-edge for various samples.** 74](#_Toc233198151)

[**Table S4.** **Fukui function values of GA and GC** 75](#_Toc233198152)

[**Table S5. Fukui function values of GG and GT** 76](#_Toc233198153)

[**Table S6. Relative parameters of different pollutants.** 77](#_Toc233198154)

[**Table S7. HPLC for each pollutant** 78](#_Toc233198155)

[**Table S8. The information of primers and amplicons for qPCR analysis.** 79](#_Toc233198156)

[**Table S9. The information related to ion chromatography detection** 80](#_Toc233198157)

[**Table S10. The comparison of the efficiency of different AOPs for ARG degradation.** 81](#_Toc233198158)

[**Table S11. The intermediate products formed during the degradation of base G in Co-NC/PMS reaction system.** 83](#_Toc233198159)

[**Table S12. The intermediate products formed during the degradation of base G in Fe-NC/PMS system.** 84](#_Toc233198160)

[**References for SI only** 86](#_Toc233198161)

**Text S1. Chemicals**

Dicyandiamide (C_2_H_4_N_4_, 98%, CAS: 461-58-5), 1,3,5-benzenetricarboxylic acid (C_9_H_6_O_6_, 98%, CAS: 528-44-9), potassium persulfate (PMS, K_5_H_3_S_4_O_18_, CAS: 70693-62-8), sodium thiosulfate (Na_2_S_2_O_3_, CAS: 7772-98-7), iron(II) chloride tetrahydrate (FeCl_2_·4H_2_O, 98%, CAS: 13478-10-9), cobalt chloride hexahydrate (CoCl_2_·6H_2_O, 99%, CAS: 7791-13-1)), nickel chloride hexahydrate (NiCl_2_·6H_2_O, 99%, CAS: 7791-20-0), copric chloride dihydrate (CuCl_2_·2H_2_O, 99%, CAS: 10125-13-0), adenine (C_5_H_5_N_5_, 98%, CAS: 73-24-5), guanine (C_5_H_5_N_5_O, 99%, CAS: 73-40-5), cytosine (C_4_H_5_N_3_O, 99%, CAS: 71-30-7), thymine (C_5_H_6_N_2_O_2_, 99%, CAS: 65-71-4), , furfuryl alcohol (C_5_H_6_O_2_, 98%, CAS: 98-00-0), β-Carotene (C_40_H_56_, 96%, CAS: 7235-40-7), dimethyl sulfoxide (DMSO, C_2_H_6_SO, >99.9%, CAS: 67-68-5), methyl phenyl sulfoxide (PMSO, C_7_H_8_OS, 98%, CAS: 1193-82-4), tert-butanol (TBA, C_4_H_10_O, >99.9%, CAS: 75-65-0), L-Histidine (C_6_H_9_N_3_O_2_, 99.5%, CAS: 71-00-1), benzoic acid (BA, C_7_H_6_O_2_, >99.9%, CAS: 65-85-0), bisphenol A (BPA, C_15_H_16_O_2_, >99,9%, CAS: 80-05-7), tetracycline (TC, C_22_H_24_N_2_O_8_, CAS: 60-54-8), sulfamethoxazole (SMZ, C_10_H_11_N_3_O_3_S, 98%, CAS: 723-46-6), ciprofloxacin (CIP, C_17_H_18_FN_3_O_3_, 98%, CAS: 85721-33-1), oxytetracycline (OTC, C_22_H_24_N_2_O_9_, >98%, CAS: 79-57-2), p-Hydroxybenzoic Acid (p-HBA, C_7_H_6_O_3_, 99.5%, CAS: 99-96-7), nitrobenzene(NB, C_6_H_5_NO_2_, 99.5%, CAS: 98-95-3), phenol(NB, C_6_H_6_O, 99%, CAS:108-95-2) were purchased from Macklin Biochemical Technology Co., Ltd (Shanghai, China). carbamazepine (CBZ, C_15_H_12_N_2_O, >98%, CAS: 298-46-4) was purchased from Solarbio Science & Technology Co., Ltd (Beijing China). The plasmid pBR322 containing *tet*A and *bla*_TEM-1_ and the pUC19 introduced with gene fragments such as *cat*, *tet*M, *kan*A and *van*A were purchased from Shanghai Sangon Biological Co., Ltd. (Shanghai, China).

**Text S2. Characterization**

The microstructure of the prepared material was observed using a SUPRATM 40 scanning electron microscope (SEM) from Zeiss in Germany and a HT7700 transmission electron microscope (TEM) from Hitachi in Japan. Record powder X-ray diffraction (XRD) on a Pananlytical X-ray diffractometer from the Netherlands to analyze the crystal structure of the catalyst. The composition elements and chemical states of the catalyst were studied by X-ray photoelectron spectroscopy (XPS) on the ESCALAB250 electron spectrometer of Thermo Fisher Corporation in the United States. The Raman spectra of the catalyst were tested using a confocal Raman spectrometer (Burker), and in-situ Raman spectroscopy was performed using an in-situ confocal Raman spectrometer (WiTech alpha300R). Use the AVIO 200 series inductively coupled plasma spectrometer (ICP) from the United States to measure the metal content and metal ion leakage in catalysts. Analysis of reactive oxygen species generated during catalyst oxidation process using a German Bruker EMXplus-6/1 paramagnetic resonance spectrometer (EPR). Time-of-flight secondary ion mass spectrometry (TOF-SIMS) measurements were performed using a TOF-SIMS 5-100 instrument (IONTOF GmbH, Germany). A Bi_3_^+^ primary ion beam with an energy of 30 keV and an ion current of 0.75 pA was employed for analysis. Spectra were acquired in high mass resolution mode over a raster area of 500 × 500 μm^2^, with a mass range of 0-1000 u.

**Text S3. Computational Details for X-ray Absorption Near-Edge Structure (XANES) Simulations**

The first-principles simulation of X-ray absorption near-edge structure (XANES) was carried out using the FDMNES code. This code employs a full multiple scattering and self-consistent real-space X-ray absorption calculation method. The final photoexcited state is obtained by solving the Green's function for the Schrödinger-like equation, with the muffin-tin approximation being applied in the process. The atomic potentials derived from density functional theory (DFT) calculations are used as the basis, and the FDMNES code is only utilized to incorporate the absorption transition matrix elements, in order to determine the most reliable XANES simulation and avoid difficulties associated with the determination of the Fermi level. The calculations take into account spin-orbit coupling, dipole, quadrupole, and core-hole contributions. In the calculation parameter settings, the Energpho function was introduced as a quasi-reference function for the absorption edge. ^[1-2]^

**Text S4. Preparation of sensory bacteria and plasmid transformation procedure**

A single DH5α colony from overnight LB culture (~16 h, 37 ºC, shaking) was transferred (1 mL) into 100 mL fresh LB and grown at 37 ºC with vigorous shaking (250-300 rpm) for 2.5-3 h. Competent cells were prepared on ice using pre-cooled 0.1 M CaCl_2_: 1.5 mL of culture was chilled 10 min, centrifuged (3000 × g, 4 ºC, 5 min), and resuspended in 100 µL CaCl_2_ for 20 min on ice. After a second centrifugation, cells were resuspended in 100 µL CaCl_2_ to obtain a uniform suspension, which could be used immediately or stored at -80 ºC with 15-20% glycerol. For transformation, 5 µL plasmid DNA was added to 100 µL competent cells, incubated on ice 30 min, heat-shocked at 42 ºC for 90 s, then chilled 2 min on ice. Following addition of 500 µL non-selective medium, cells were recovered at 37 ºC, 1 h shaking, centrifuged, resuspended, and plated on LB agar with appropriate antibiotics. Plates were incubated at 37 ºC for 12-16 h to allow colony formation.

**Text S5. Normalized kinetic analysis for cross-system comparison**

Direct comparison of catalytic oxidation performance reported in previous studies is often challenging because degradation efficiency is simultaneously affected by multiple experimental variables, including catalyst dosage, oxidant concentration, substrate concentration, and reaction configuration. This issue becomes particularly significant for nucleic-acid-related targets due to the large variation in initial concentrations and oxidation conditions among studies. Therefore, a normalized kinetic framework was introduced to enable a more reliable evaluation of intrinsic catalytic activity.

To minimize systematic deviations, only studies employing the same oxidant category (PMS activation systems) were included in the comparison. G, which represents the most oxidation-sensitive nucleobase and serves as a molecular surrogate for oxidative nucleic acid damage, was selected as the unified target molecule.

The normalized degradation rate constant was calculated according to Equation (S1):

$k_{norm}=k\times C_{cat}/{(C}_{cat}\times C_{0x})$ Equation S1

where (k_norm_) (L·g^-2^·µmol^-1^·min^-1^) is the normalized kinetic constant, (k_obs_) (min^-1^) is the pseudo-first-order degradation rate constant obtained from kinetic fitting, (C_cat_) (g·L^-1^) is the catalyst dosage, (C_ox_) (g·L^-1^) is the PMS concentration, and (C_sub_) (µmol·L^-1^) is the initial G concentration.

This normalization strategy eliminates the apparent enhancement originating from excessive catalyst or oxidant inputs and enables comparison of intrinsic oxidation capability across different PMS-based catalytic systems. It should be noted that the normalized constant is intended for relative performance benchmarking rather than mechanistic kinetic modeling, and therefore does not imply a strict reaction order with respect to catalyst, oxidant, or substrate concentrations.

**Text S6. Quantitative analysis of steady-state concentration of ROSs**

NB, BA, and FFA were used as probe compounds to determine the steady-state concentrations of ROS (•OH, SO_4_^•-^, and ^1^O_2_)^1-3^. The steady-state concentration equations for •OH and SO_4_^•-^ radicals are as follows:

$$k_{NB}^{'}t=ln\frac{[NB]_{0}}{[NB]}=k_{NB-\bullet OH}{[\bullet OH]}_{SS}t$$

$$k_{BA}^{'}t=ln\frac{[BA]_{0}}{[BA]}=k_{BA-\bullet OH}{[\bullet OH]}_{SS}t+k_{BA-\bullet SO_{4}^{-}}[SO_{4}^{\bullet-}]_{SS}t$$

By combining the above two equations, we can obtain:

$${[\bullet OH]}_{SS}=\frac{k_{NB}^{'}}{k_{NB-\bullet OH}}$$

$${[SO_{4}^{\bullet-}]}_{SS}=\frac{k_{NB}^{'}-k_{BA-\bullet OH[\bullet OH]_{SS}}}{k_{BA-{SO}_{4}^{\bullet-}}}$$

Among them, $k_{NB}^{'}$and $k_{BA}^{'}$ are the apparent reaction rate constants of NB and BA, [NB]_0_ and [BA]_0_ are the initial concentrations of NB and BA, and [NB] and [BA] are the concentrations of NB and BA at the reaction time (t). $k_{NB-\bullet OH}$ is the second-order rate constant of NB and •OH (3.9 × 10^9^ M^-1^ s^-1^), while $k_{BA-\bullet OH}$ (5.9 × 10^9^ M^-1^ s^-1^), and $k_{BA-{SO}_{4}^{\bullet-}}$ (1.2 × 10^9^ M^-1^ s^-1^) are the second-order rate constants of •OH and SO_4_^•-^ with BA, respectively. [•OH] ss and [SO_4_^•-^] ss are the steady-state concentrations of • OH and SO_4_^•-^.

The steady-state concentration equation of singlet oxygen is as follows:

$$k_{FFA}^{'}t=ln\frac{[FFA]_{0}}{[FFA]}={(k}_{FFA-\bullet OH}{[\bullet OH]}_{SS}t+k_{FFA-{{}^{1}O}_{2}}[{}^{1}{O_{2}}]_{SS})t$$

$${[{}^{1}{O_{2}}]}_{SS}=\frac{k_{FFA}^{'}-k_{FFA-\bullet OH[\bullet OH]_{SS}}}{k_{NB-\bullet OH}}$$

Among them, $k_{FFA}^{'}$is the apparent reaction rate constant of FFA, and [FFA]_0_ and [FFA] are the initial and reaction time (t) BPA concentrations. $k_{FFA-\bullet OH}$ (3.6 × 10^9^ M^-1^ s^-1^) and $k_{FFA-{{}^{1}O}_{2}}$ (1.2 × 10^8^ M^-1^ s^-1^) are the second-order rate constants of •OH and ^1^O_2_ with FFA, respectively. Add NB, BA, and FFA with an initial concentration of 50 μM to a single atom PMS system, and monitor the concentrations of NB, BA, and FFA by HPLC.

**Text S7. ARGs sequence**

***bla*_TEM-1_ (861bp)**

TTACCAATGCTTAATCAGTGAGGCACCTATCTCAGCGATCTGTCTATTTCGTTCATCCATAGTTGCCTGACTCCCCGTCGTGTAGATAACTACGATACGGGAGGGCTTACCATCTGGCCCCAGTGCTGCAATGATACCGCGAGACCCACGCTCACCGGCTCCAGATTTATCAGCAATAAACCAGCCAGCCGGAAGGGCCGAGCGCAGAAGTGGTCCTGCAACTTTATCCGCCTCCATCCAGTCTATTAATTGTTGCCGGGAAGCTAGAGTAAGTAGTTCGCCAGTTAATAGTTTGCGCAACGTTGTTGCCATTGCTGCAGGCATCGTGGTGTCACGCTCGTCGTTTGGTATGGCTTCATTCAGCTCCGGTTCCCAACGATCAAGGCGAGTTACATGATCCCCCATGTTGTGCAAAAAAGCGGTTAGCTCCTTCGGTCCTCCGATCGTTGTCAGAAGTAAGTTGGCCGCAGTGTTATCACTCATGGTTATGGCAGCACTGCATAATTCTCTTACTGTCATGCCATCCGTAAGATGCTTTTCTGTGACTGGTGAGTACTCAACCAAGTCATTCTGAGAATAGTGTATGCGGCGACCGAGTTGCTCTTGCCCGGCGTCAACACGGGATAATACCGCGCCACATAGCAGAACTTTAAAAGTGCTCATCATTGGAAAACGTTCTTCGGGGCGAAAACTCTCAAGGATCTTACCGCTGTTGAGATCCAGTTCGATGTAACCCACTCGTGCACCCAACTGATCTTCAGCATCTTTTACTTTCACCAGCGTTTCTGGGTGAGCAAAAACAGGAAGGCAAAATGCCGCAAAAAAGGGAATAAGGGCGACACGGAAATGTTGAATACTCAT

***tet*A (1191bp)**

ATGAAATCTAACAATGCGCTCATCGTCATCCTCGGCACCGTCACCCTGGATGCTGTAGGCATAGGCTTGGTTATGCCGGTACTGCCGGGCCTCTTGCGGGATATCGTCCATTCCGACAGCATCGCCAGTCACTATGGCGTGCTGCTAGCGCTATATGCGTTGATGCAATTTCTATGCGCACCCGTTCTCGGAGCACTGTCCGACCGCTTTGGCCGCCGCCCAGTCCTGCTCGCTTCGCTACTTGGAGCCACTATCGACTACGCGATCATGGCGACCACACCCGTCCTGTGGATCCTCTACGCCGGACGCATCGTGGCCGGCATCACCGGCGCCACAGGTGCGGTTGCTGGCGCCTATATCGCCGACATCACCGATGGGGAAGATCGGGCTCGCCACTTCGGGCTCATGAGCGCTTGTTTCGGCGTGGGTATGGTGGCAGGCCCCGTGGCCGGGGGACTGTTGGGCGCCATCTCCTTGCATGCACCATTCCTTGCGGCGGCGGTGCTCAACGGCCTCAACCTACTACTGGGCTGCTTCCTAATGCAGGAGTCGCATAAGGGAGAGCGTCGACCGATGCCCTTGAGAGCCTTCAACCCAGTCAGCTCCTTCCGGTGGGCGCGGGGCATGACTATCGTCGCCGCACTTATGACTGTCTTCTTTATCATGCAACTCGTAGGACAGGTGCCGGCAGCGCTCTGGGTCATTTTCGGCGAGGACCGCTTTCGCTGGAGCGCGACGATGATCGGCCTGTCGCTTGCGGTATTCGGAATCTTGCACGCCCTCGCTCAAGCCTTCGTCACTGGTCCCGCCACCAAACGTTTCGGCGAGAAGCAGGCCATTATCGCCGGCATGGCGGCCGACGCGCTGGGCTACGTCTTGCTGGCGTTCGCGACGCGAGGCTGGATGGCCTTCCCCATTATGATTCTTCTCGCTTCCGGCGGCATCGGGATGCCCGCGTTGCAGGCCATGCTGTCCAGGCAGGTAGATGACGACCATCAGGGACAGCTTCAAGGATCGCTCGCGGCTCTTACCAGCCTAACTTCGATCACTGGACCGCTGATCGTCACGGCGATTTATGCCGCCTCGGCGAGCACATGGAACGGGTTGGCATGGATTGTAGGCGCCGCCCTATACCTTGTCTGCCTCCCCGCGTTGCGTCGCGGTGCATGGAGCCGGGCCACCTCGACCTGA

***tet*M (1905bp)**

ATGAAAATTATTAATATTGGAGTTTTAGCTCATGTTGATGCAGGAAAAACTACCTTAACAGAAAGCTTATTATATAACAGTGGAGCGATTACAGAATTAGGAAGCGTGGACAAAGGTACAACGAGGACGGATAATACGCTTTTAGAACGTCAGAGAGGAATTACAATTCAGACAGGAATAACCTCTTTTCAGTGGGAAAATACGAAGGTGAACATCATAGACACGCCAGGACATATGGATTTCTTAGCAGAAGTATATCGTTCATTATCAGTTTTAGATGGGGCAATTCTACTGATTTCTGCAAAAGATGGCGTACAAGCACAAACTCGTATATTATTTCATGCACTTAGGAAAATGGGGATTCCCACAATCTTTTTTATCAATAAGATTGACCAAAATGGAATTGATTTATCAACGGTTTATCAGGATATTAAAGAGAAACTTTCTGCCGAAATTGTAATCAAACAGAAGGTAGAACTGTATCCTAATATGTGTGTGACGAACTTTACCGAATCTGAACAATGGGATACGGTAATAGAGGGAAACGATGACCTTTTAGAGAAATATATGTCCGGTAAATCATTAGAAGCATTGGAACTCGAACAAGAGGAAAGCATAAGATTTCATAATTGTTCCCTGTTCCCTGTTTATCACGGAAGTGCAAAAAACAATATAGGGATTGATAACCTTATAGAAGTGATTACGAATAAATTTTATTCATCAACACATCGAGGTCCGTCTGAACTTTGCGGAAATGTTTTCAAAATTGAATATACAAAAAAAAGACAACGTCTTGCATATATACGCCTTTATAGTGGAGTACTACATTTACGAGATTCGGTTAGAGTATCAGAAAAAGAAAAAATAAAAGTTACAGAAATGTATACTTCAATAAATGGTGAATTATGTAAGATTGATAGAGCTTATTCTGGAGAAATTGTTATTTTGCAAAATGAGTTTTTGAAGTTAAATAGTGTTCTTGGAGATACAAAACTATTGCCACAGAGAAAAAAGATTGAAAATCCGCACCCTCTACTACAAACAACTGTTGAACCGAGTAAACCTGAACAGAGAGAAATGTTGCTTGATGCCCTTTTGGAAATCTCAGATAGTGATCCGCTTCTACGATATTACGTGGATTCTACGACACATGAAATTATACTTTCTTTCTTAGGGAAAGTACAAATGGAAGTGATTAGTGCACTGTTGCAAGAAAAGTATCATGTGGAGATAGAACTAAAAGAGCCTACAGTCATTTATATGGAGAGACCGTTAAAAAATGCAGAATATACCATTCACATCGAAGTGCCGCCAAATCCTTTCTGGGCTTCCATTGGTTTATCTGTATCACCGCTTCCGTTGGGAAGTGGAATGCAGTATGAGAGCTCGGTTTCTCTTGGATACTTAAATCAATCATTTCAAAATGCAGTTATGGAAGGGATACGCTATGGTTGCGAACAAGGATTATATGGTTGGAATGTGACGGATTGTAAAATCTGTTTTAAGTATGGCTTATACTATAGCCCTGTTAGTACCCCAGCAGATTTTCGGATGCTTGCTCCTATTGTATTGGAACAAGTCTTAAAAAAAGCTGGAACAGAATTGTTAGAGCCATATCTTAGTTTTAAAATTTATGCGCCACAGGAATATCTTTCACGAGCATACAACGATGCTCCTAAATATTGTGCGAACATCGTAGACACTCAATTGAAAAATAATGAGGTCATTCTTAGTGGAGAAATCCCTGCTCGGTGTATTCAAGAATATCGTAGTGATTTAACTTTCTTTACAAATGGACGTAGTGTTTGTTTAACAGAGTTAAAAGGGTACCATGTTACTACCGGTGAACCTGTTTGCCAGCCCCGTCGTCCAAATAGTCGGATAGATAAAGTACGATATATGTTC

***cat* (660bp)**

TTACGCCCCGCCCTGCCACTCATCGCAGTACTGTTGTAATTCATTAAGCATTCTGCCGACATGGAAGCCATCACAAACGGCATGATGAACCTGAATCGCCAGCGGCATCAGCACCTTGTCGCCTTGCGTATAATATTTGCCCATAGTGAAAACGGGGGCGAAGAAGTTGTCCATATTGGCCACGTTTAAATCAAAACTGGTGAAACTCACCCAGGGATTGGCTGAGACGAAAAACATATTCTCAATAAACCCTTTAGGGAAATAGGCCAGGTTTTCACCGTAACACGCCACATCTTGCGAATATATGTGTAGAAACTGCCGGAAATCGTCGTGGTATTCACTCCAGAGCGATGAAAACGTTTCAGTTTGCTCATGGAAAACGGTGTAACAAGGGTGAACACTATCCCATATCACCAGCTCACCGTCTTTCATTGCCATACGGAACTCCGGATGAGCATTCATCAGGCGGGCAAGAATGTGAATAAAGGCCGGATAAAACTTGTGCTTATTTTTCTTTACGGTCTTTAAAAAGGCCGTAATATCCAGCTGAACGGTCTGGTTATAGGTACATTGAGCAACTGACTGAAATGCCTCAAAATGTTCTTTACGATGCCATTGGGATATATCAACGGTGGTATATCCAGTGATTTTTTTCTCCAT

***kan*A (816bp)**

TTAGAAAAACTCATCGAGCATCAAATGAAACTGCAATTTATTCATATCAGGATTATCAATACCATATTTTTGAAAAAGCCGTTTCTGTAATGAAGGAGAAAACTCACCGAGGCAGTTCCATAGGATGGCAAGATCCTGGTATCGGTCTGCGATTCCGACTCGTCCAACATCAATACAACCTATTAATTTCCCCTCGTCAAAAATAAGGTTATCAAGTGAGAAATCACCATGAGTGACGACTGAATCCGGTGAGAATGGCAAAAGCTTATGCATTTCTTTCCAGACTTGTTCAACAGGCCAGCCATTACGCTCGTCATCAAAATCACTCGCATCAACCAAACCGTTATTCATTCGTGATTGCGCCTGAGCGAGACGAAATACGCGATCGCTGTTAAAAGGACAATTACAAACAGGAATCGAATGCAACCGGCGCAGGAACACTGCCAGCGCATCAACAATATTTTCACCTGAATCAGGATATTCTTCTAATACCTGGAATGCTGTTTTCCCGGGGATCGCAGTGGTGAGTAACCATGCATCATCAGGAGTACGGATAAAATGCTTGATGGTCGGAAGAGGCATAAATTCCGTCAGCCAGTTTAGTCTGACCATCTCATCTGTAACATCATTGGCAACGCTACCTTTGCCATGTTTCAGAAACAACTCTGGCGCATCGGGCTTCCCATACAATCGATAGATTGTCGCACCTGATTGCCCGACATTATCGCGAGCCCATTTATACCCATATAAATCAGCATCCATGTTGGAATTTAATCGCGGCCTCGAGCAAGACGTTTCCCGTTGAATATGGCTCAT

***van*A (1032bp)**

ATGAATAGAATAAAAGTTGCAATACTGTTTGGGGGTTGCTCAGAGGAGCATGACGTATCGGTAAAATCTGCAATAGAGATAGCCGCTAACATTAATAAAGAAAAATACGAGCCGTTATACATTGGAATTACGAAATCTGGTGTATGGAAAATGTGCGAAAAACCTTGCGCGGAATGGGAAAACGACAATTGCTATTCAGCTGTACTCTCGCCGGATAAAAAAATGCACGGATTACTTGTTAAAAAGAACCATGAATATGAAATCAACCATGTTGATGTAGCATTTTCAGCTTTGCATGGCAAGTCAGGTGAAGATGGATCCATACAAGGTCTGTTTGAATTGTCCGGTATCCCTTTTGTAGGCTGCGATATTCAAAGCTCAGCAATTTGTATGGACAAATCGTTGACATACATCGTTGCGAAAAATGCTGGGATAGCTACTCCCGCCTTTTGGGTTATTAATAAAGATGATAGGCCGGTGGCAGCTACGTTTACCTATCCTGTTTTTGTTAAGCCGGCGCGTTCAGGCTCATCCTTCGGTGTGAAAAAAGTCAATAGCGCGGACGAATTGGACTACGCAATTGAATCGGCAAGACAATATGACAGCAAAATCTTAATTGAGCAGGCTGTTTCGGGCTGTGAGGTCGGTTGTGCGGTATTGGGAAACAGTGCCGCGTTAGTTGTTGGCGAGGTGGACCAAATCAGGCTGCAGTACGGAATCTTTCGTATTCATCAGGAAGTCGAGCCGGAAAAAGGCTCTGAAAACGCAGTTATAACCGTTCCCGCAGACCTTTCAGCAGAGGAGCGAGGACGGATACAGGAAACGGCAAAAAAAATATATAAAGCGCTCGGCTGTAGAGGTCTAGCCCGTGTGGATATGTTTTTACAAGATAACGGCCGCATTGTACTGAACGAAGTCAATACTCTGCCCGGTTTCACGTCATACAGTCGTTATCCCCGTATGATGGCCGCTGCAGGTATTGCACTTCCCGAACTGATTGACCGCTTGATCGTATTAGCGTTAAAGGGGTGA

***amp*R (861bp)**

ATGAGTATTCAACATTTCCGTGTCGCCCTTATTCCCTTTTTTGCGGCATTTTGCCTTCCTGTTTTTGCTCACCCAGAAACGCTGGTGAAAGTAAAAGATGCTGAAGATCAGTTGGGTGCACGAGTGGGTTACATCGAACTGGATCTCAACAGCGGTAAGATCCTTGAGAGTTTTCGCCCCGAAGAACGTTTTCCAATGATGAGCACTTTTAAAGTTCTGCTATGTGGCGCGGTATTATCCCGTATTGACGCCGGGCAAGAGCAACTCGGTCGCCGCATACACTATTCTCAGAATGACTTGGTTGAGTACTCACCAGTCACAGAAAAGCATCTTACGGATGGCATGACAGTAAGAGAATTATGCAGTGCTGCCATAACCATGAGTGATAACACTGCGGCCAACTTACTTCTGACAACGATCGGAGGACCGAAGGAGCTAACCGCTTTTTTGCACAACATGGGGGATCATGTAACTCGCCTTGATCGTTGGGAACCGGAGCTGAATGAAGCCATACCAAACGACGAGCGTGACACCACGATGCCTGCAGCAATGGCAACAACGTTGCGCAAACTATTAACTGGCGAACTACTTACTCTAGCTTCCCGGCAACAATTAATAGACTGGATGGAGGCGGATAAAGTTGCAGGACCACTTCTGCGCTCGGCCCTTCCGGCTGGCTGGTTTATTGCTGATAAATCTGGAGCCGGTGAGCGTGGGTCTCGCGGTATCATTGCAGCACTGGGGCCAGATGGTAAGCCCTCCCGTATCGTAGTTATCTACACGACGGGGAGTCAGGCAACTATGGATGAACGAAATAGACAGATCGCTGAGATAGGTGCCTCACTGATTAAGCATTGGTAA

***pBR*322 (4361bp)**

ATTGTCTCATGAGCGGATACATATTTGAATGTATTTAGAAAAATAAACAAATAGGGGTTCCGCGCACATTTCCCCGAAAAGTGCCACCTGACGTCTAAGAAACCATTATTATCATGACATTAACCTATAAAAATAGGCGTATCACGAGGCCCTTTCGTCTTCAAGAATTCTCATGTTTGACAGCTTATCATCGATAAGCTTTAATGCGGTAGTTTATCACAGTTAAATTGCTAACGCAGTCAGGCACCGTGTATGAAATCTAACAATGCGCTCATCGTCATCCTCGGCACCGTCACCCTGGATGCTGTAGGCATAGGCTTGGTTATGCCGGTACTGCCGGGCCTCTTGCGGGATATCGTCCATTCCGACAGCATCGCCAGTCACTATGGCGTGCTGCTAGCGCTATATGCGTTGATGCAATTTCTATGCGCACCCGTTCTCGGAGCACTGTCCGACCGCTTTGGCCGCCGCCCAGTCCTGCTCGCTTCGCTACTTGGAGCCACTATCGACTACGCGATCATGGCGACCACACCCGTCCTGTGGATCCTCTACGCCGGACGCATCGTGGCCGGCATCACCGGCGCCACAGGTGCGGTTGCTGGCGCCTATATCGCCGACATCACCGATGGGGAAGATCGGGCTCGCCACTTCGGGCTCATGAGCGCTTGTTTCGGCGTGGGTATGGTGGCAGGCCCCGTGGCCGGGGGACTGTTGGGCGCCATCTCCTTGCATGCACCATTCCTTGCGGCGGCGGTGCTCAACGGCCTCAACCTACTACTGGGCTGCTTCCTAATGCAGGAGTCGCATAAGGGAGAGCGTCGACCGATGCCCTTGAGAGCCTTCAACCCAGTCAGCTCCTTCCGGTGGGCGCGGGGCATGACTATCGTCGCCGCACTTATGACTGTCTTCTTTATCATGCAACTCGTAGGACAGGTGCCGGCAGCGCTCTGGGTCATTTTCGGCGAGGACCGCTTTCGCTGGAGCGCGACGATGATCGGCCTGTCGCTTGCGGTATTCGGAATCTTGCACGCCCTCGCTCAAGCCTTCGTCACTGGTCCCGCCACCAAACGTTTCGGCGAGAAGCAGGCCATTATCGCCGGCATGGCGGCCGACGCGCTGGGCTACGTCTTGCTGGCGTTCGCGACGCGAGGCTGGATGGCCTTCCCCATTATGATTCTTCTCGCTTCCGGCGGCATCGGGATGCCCGCGTTGCAGGCCATGCTGTCCAGGCAGGTAGATGACGACCATCAGGGACAGCTTCAAGGATCGCTCGCGGCTCTTACCAGCCTAACTTCGATCATTGGACCGCTGATCGTCACGGCGATTTATGCCGCCTCGGCGAGCACATGGAACGGGTTGGCATGGATTGTAGGCGCCGCCCTATACCTTGTCTGCCTCCCCGCGTTGCGTCGCGGTGCATGGAGCCGGGCCACCTCGACCTGAATGGAAGCCGGCGGCACCTCGCTAACGGATTCACCACTCCAAGAATTGGAGCCAATCAATTCTTGCGGAGAACTGTGAATGCGCAAACCAACCCTTGGCAGAACATATCCATCGCGTCCGCCATCTCCAGCAGCCGCACGCGGCGCATCTCGGGCAGCGTTGGGTCCTGGCCACGGGTGCGCATGATCGTGCTCCTGTCGTTGAGGACCCGGCTAGGCTGGCGGGGTTGCCTTACTGGTTAGCAGAATGAATCACCGATACGCGAGCGAACGTGAAGCGACTGCTGCTGCAAAACGTCTGCGACCTGAGCAACAACATGAATGGTCTTCGGTTTCCGTGTTTCGTAAAGTCTGGAAACGCGGAAGTCAGCGCCCTGCACCATTATGTTCCGGATCTGCATCGCAGGATGCTGCTGGCTACCCTGTGGAACACCTACATCTGTATTAACGAAGCGCTGGCATTGACCCTGAGTGATTTTTCTCTGGTCCCGCCGCATCCATACCGCCAGTTGTTTACCCTCACAACGTTCCAGTAACCGGGCATGTTCATCATCAGTAACCCGTATCGTGAGCATCCTCTCTCGTTTCATCGGTATCATTACCCCCATGAACAGAAATCCCCCTTACACGGAGGCATCAGTGACCAAACAGGAAAAAACCGCCCTTAACATGGCCCGCTTTATCAGAAGCCAGACATTAACGCTTCTGGAGAAACTCAACGAGCTGGACGCGGATGAACAGGCAGACATCTGTGAATCGCTTCACGACCACGCTGATGAGCTTTACCGCAGCTGCCTCGCGCGTTTCGGTGATGACGGTGAAAACCTCTGACACATGCAGCTCCCGGAGACGGTCACAGCTTGTCTGTAAGCGGATGCCGGGAGCAGACAAGCCCGTCAGGGCGCGTCAGCGGGTGTTGGCGGGTGTCGGGGCGCAGCCATGACCCAGTCACGTAGCGATAGCGGAGTGTATACTGGCTTAACTATGCGGCATCAGAGCAGATTGTACTGAGAGTGCACCATATGCGGTGTGAAATACCGCACAGATGCGTAAGGAGAAAATACCGCATCAGGCGCTCTTCCGCTTCCTCGCTCACTGACTCGCTGCGCTCGGTCGTTCGGCTGCGGCGAGCGGTATCAGCTCACTCAAAGGCGGTAATACGGTTATCCACAGAATCAGGGGATAACGCAGGAAAGAACATGTGAGCAAAAGGCCAGCAAAAGGCCAGGAACCGTAAAAAGGCCGCGTTGCTGGCGTTTTTCCATAGGCTCCGCCCCCCTGACGAGCATCACAAAAATCGACGCTCAAGTCAGAGGTGGCGAAACCCGACAGGACTATAAAGATACCAGGCGTTTCCCCCTGGAAGCTCCCTCGTGCGCTCTCCTGTTCCGACCCTGCCGCTTACCGGATACCTGTCCGCCTTTCTCCCTTCGGGAAGCGTGGCGCTTTCTCATAGCTCACGCTGTAGGTATCTCAGTTCGGTGTAGGTCGTTCGCTCCAAGCTGGGCTGTGTGCACGAACCCCCCGTTCAGCCCGACCGCTGCGCCTTATCCGGTAACTATCGTCTTGAGTCCAACCCGGTAAGACACGACTTATCGCCACTGGCAGCAGCCACTGGTAACAGGATTAGCAGAGCGAGGTATGTAGGCGGTGCTACAGAGTTCTTGAAGTGGTGGCCTAACTACGGCTACACTAGAAGGACAGTATTTGGTATCTGCGCTCTGCTGAAGCCAGTTACCTTCGGAAAAAGAGTTGGTAGCTCTTGATCCGGCAAACAAACCACCGCTGGTAGCGGTGGTTTTTTTGTTTGCAAGCAGCAGATTACGCGCAGAAAAAAAGGATCTCAAGAAGATCCTTTGATCTTTTCTACGGGGTCTGACGCTCAGTGGAACGAAAACTCACGTTAAGGGATTTTGGTCATGAGATTATCAAAAAGGATCTTCACCTAGATCCTTTTAAATTAAAAATGAAGTTTTAAATCAATCTAAAGTATATATGAGTAAACTTGGTCTGACAGTTACCAATGCTTAATCAGTGAGGCACCTATCTCAGCGATCTGTCTATTTCGTTCATCCATAGTTGCCTGACTCCCCGTCGTGTAGATAACTACGATACGGGAGGGCTTACCATCTGGCCCCAGTGCTGCAATGATACCGCGAGACCCACGCTCACCGGCTCCAGATTTATCAGCAATAAACCAGCCAGCCGGAAGGGCCGAGCGCAGAAGTGGTCCTGCAACTTTATCCGCCTCCATCCAGTCTATTAATTGTTGCCGGGAAGCTAGAGTAAGTAGTTCGCCAGTTAATAGTTTGCGCAACGTTGTTGCCATTGCTGCAGGCATCGTGGTGTCACGCTCGTCGTTTGGTATGGCTTCATTCAGCTCCGGTTCCCAACGATCAAGGCGAGTTACATGATCCCCCATGTTGTGCAAAAAAGCGGTTAGCTCCTTCGGTCCTCCGATCGTTGTCAGAAGTAAGTTGGCCGCAGTGTTATCACTCATGGTTATGGCAGCACTGCATAATTCTCTTACTGTCATGCCATCCGTAAGATGCTTTTCTGTGACTGGTGAGTACTCAACCAAGTCATTCTGAGAATAGTGTATGCGGCGACCGAGTTGCTCTTGCCCGGCGTCAACACGGGATAATACCGCGCCACATAGCAGAACTTTAAAAGTGCTCATCATTGGAAAACGTTCTTCGGGGCGAAAACTCTCAAGGATCTTACCGCTGTTGAGATCCAGTTCGATGTAACCCACTCGTGCACCCAACTGATCTTCAGCATCTTTTACTTTCACCAGCGTTTCTGGGTGAGCAAAAACAGGAAGGCAAAATGCCGCAAAAAAGGGAATAAGGGCGACACGGAAATGTTGAATACTCATACTCTTCCTTTTTCAATATTATTGAAGCATTTATCAGGGTT

The DNA fragment marked above serves as the primer segment for ARG, with the specific primers detailed in the Table S5.


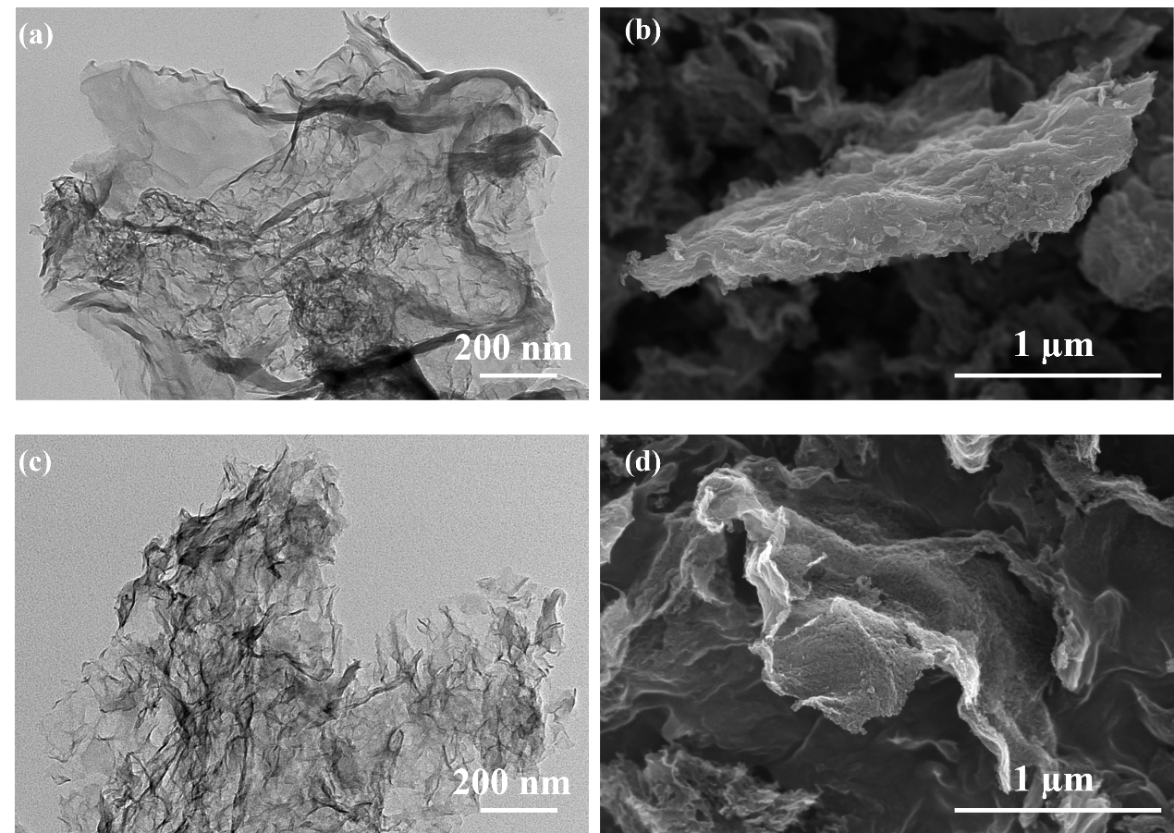


**Figure S1.** TEM (a) and SEM (b) images of Co-NC and TEM (c) and SEM (d) images of Fe-NC.


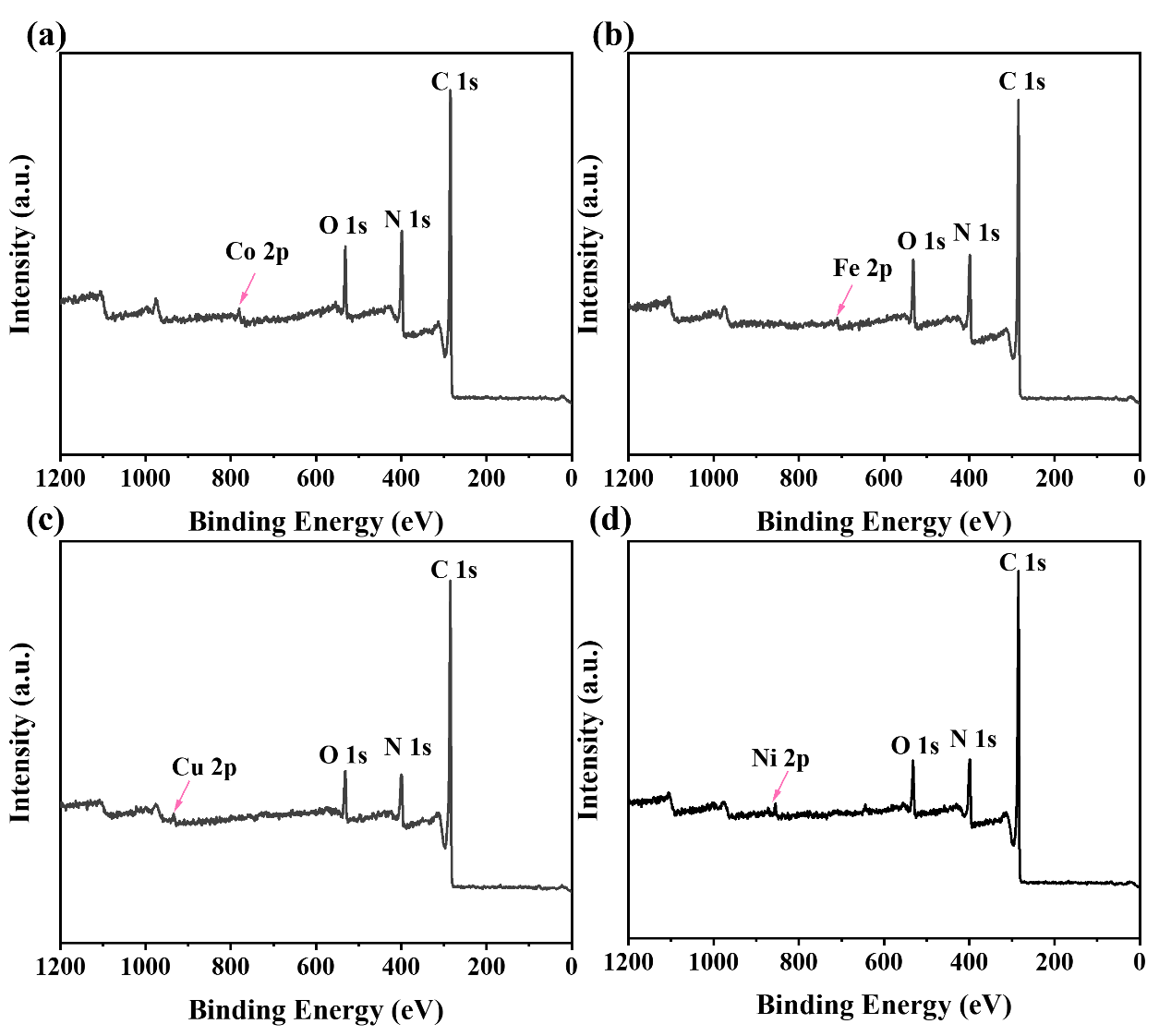


**Figure S2.** Full XPS spectra of Co-NC, Fe-NC, Cu-NC and Ni-NC.


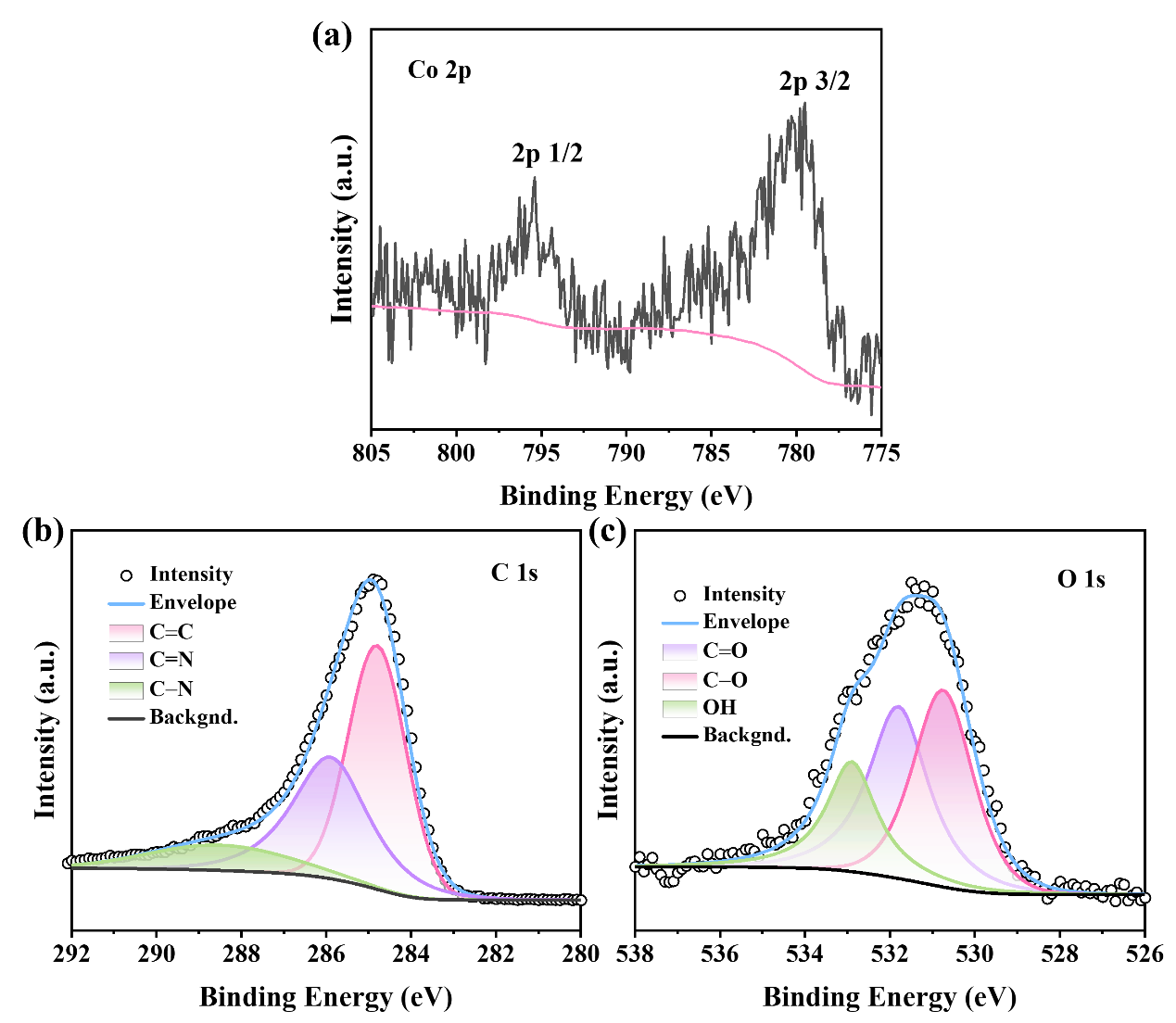


**Figure S3.** The high-resolution XPS spectra of the Co-NC: (a) Co 2p, (b) C 1s and (c) O 1s.

**
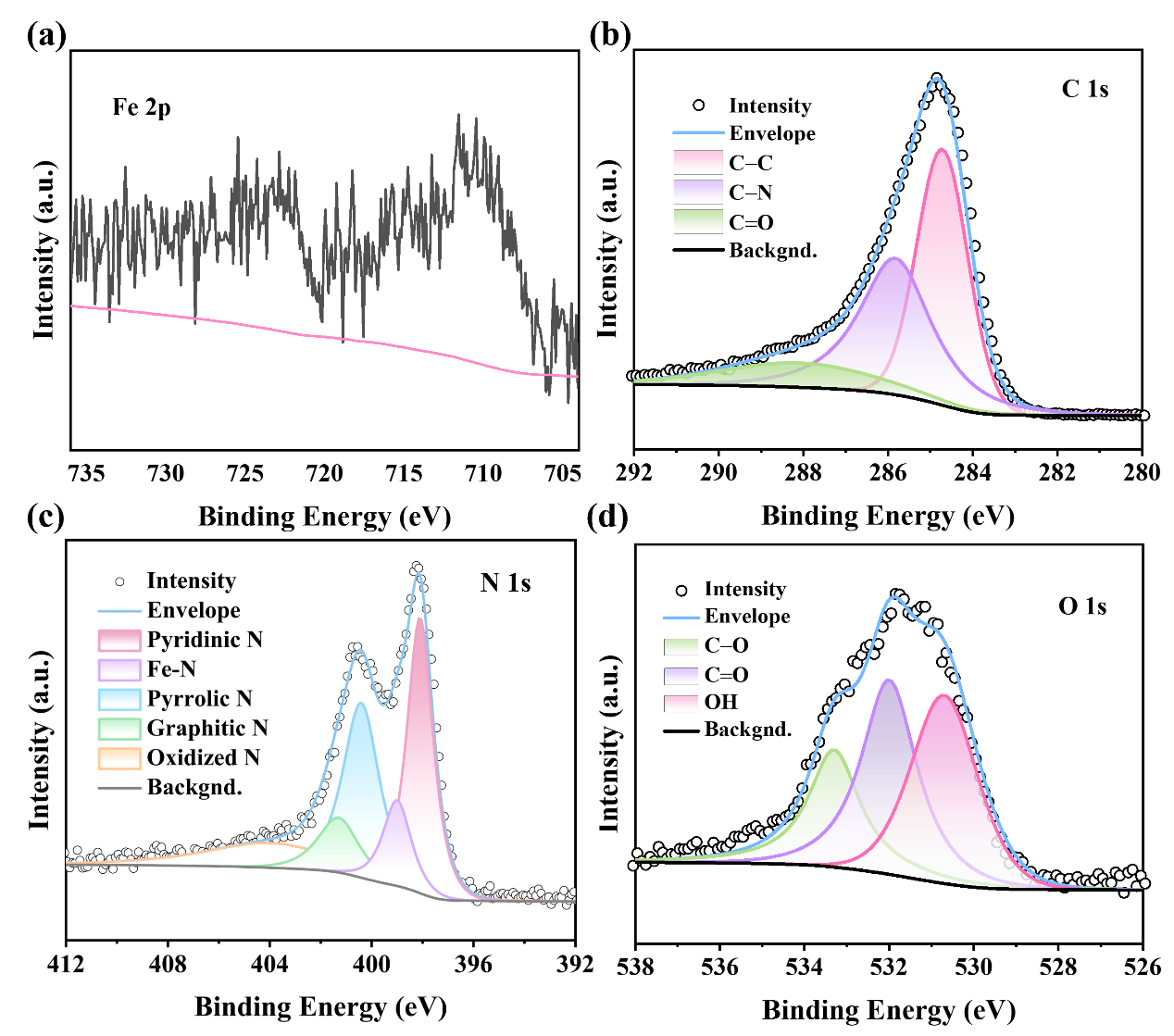
**

**Figure S4.** The high-resolution XPS spectra of the Fe-NC: (a) Fe 2p, (b) C 1 s, (c) N 1s and (d) O 1s.


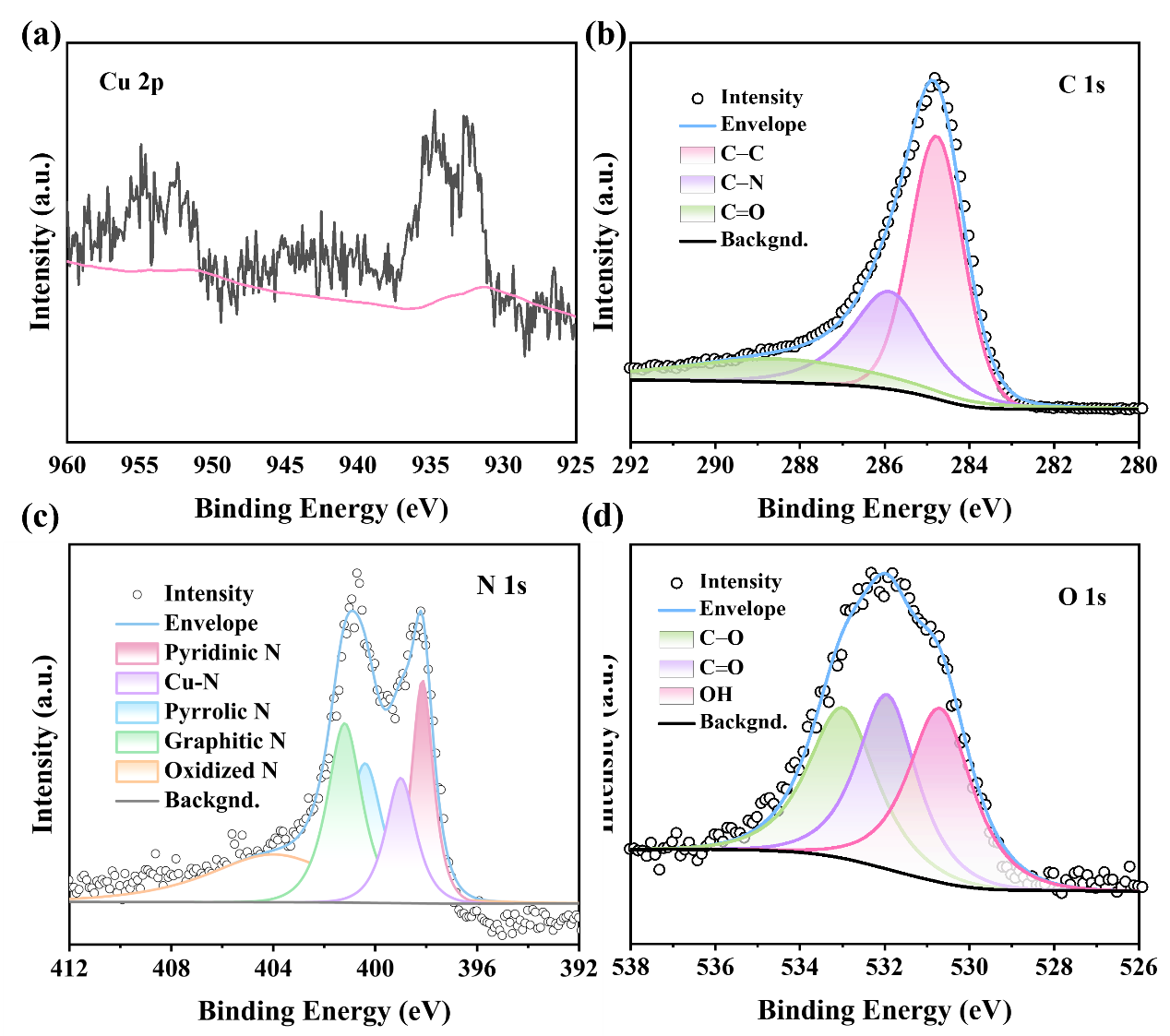


**Figure S5.** The high-resolution XPS spectra of the Cu-NC: (a) Cu 2p, (b) C 1 s, (c) N 1s and (d) O 1s.


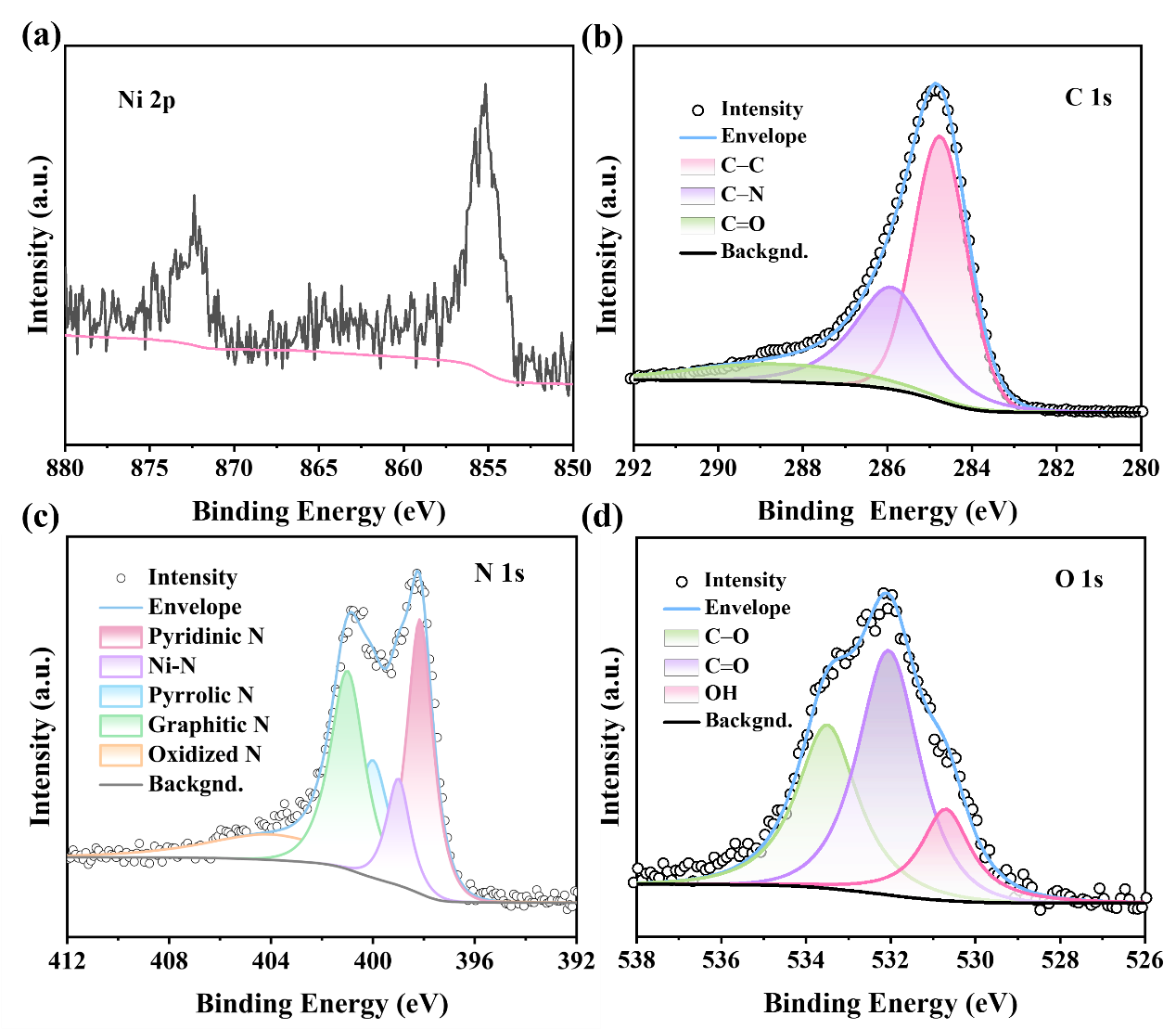


**Figure S6.** The high-resolution XPS spectra of the Ni-NC: (a) Ni 2p, (b) C 1 s, (c) N 1s and (d) O 1s.


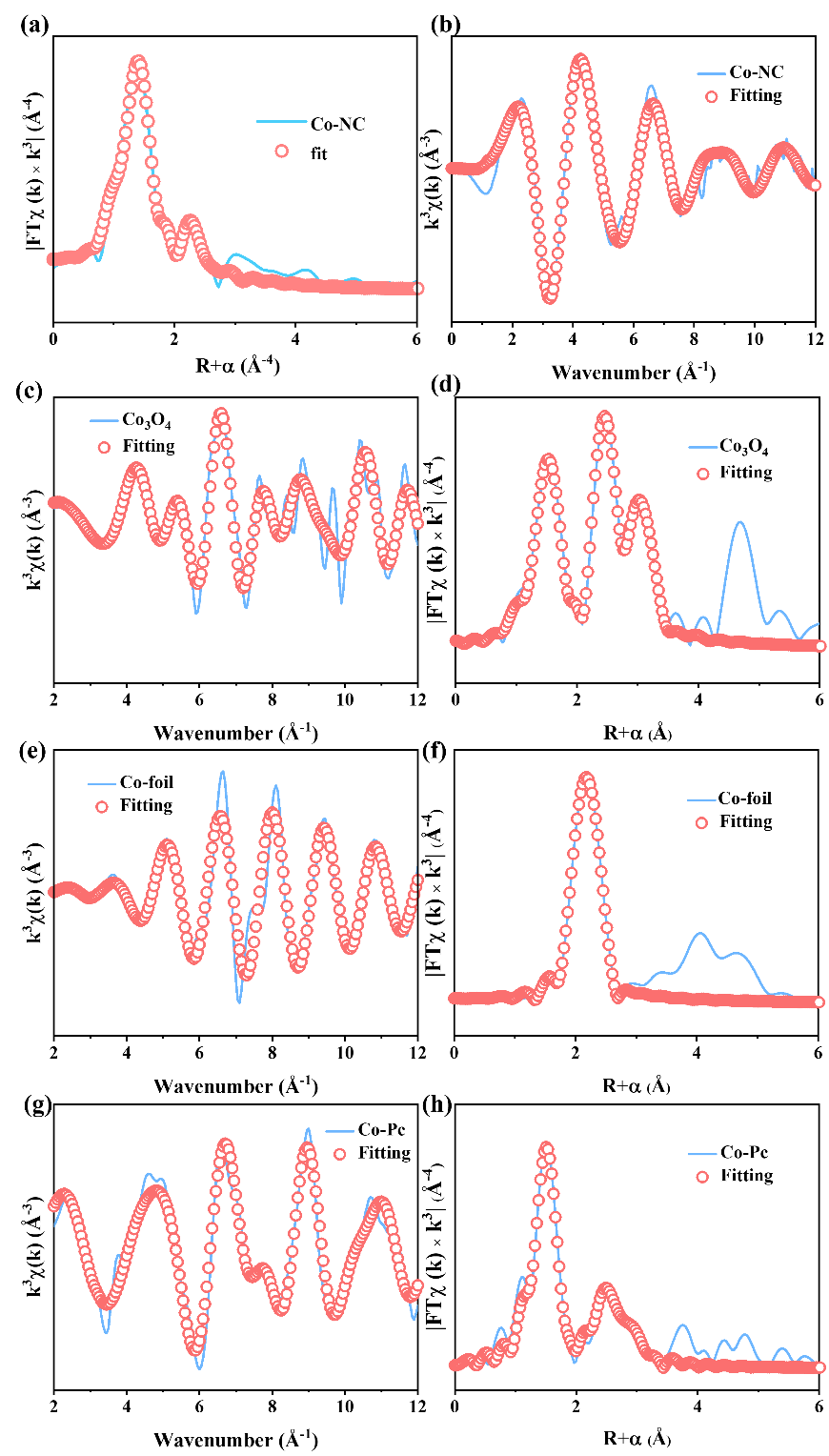


**Figure S7.** The Fourier transformed k3-weight EXAFS curve and curve fit line in k space and R space for (a-b) Co-NC, (c-d) Co_3_O_4_, (e-f) Co-foil, (g-h) Co-Pc.


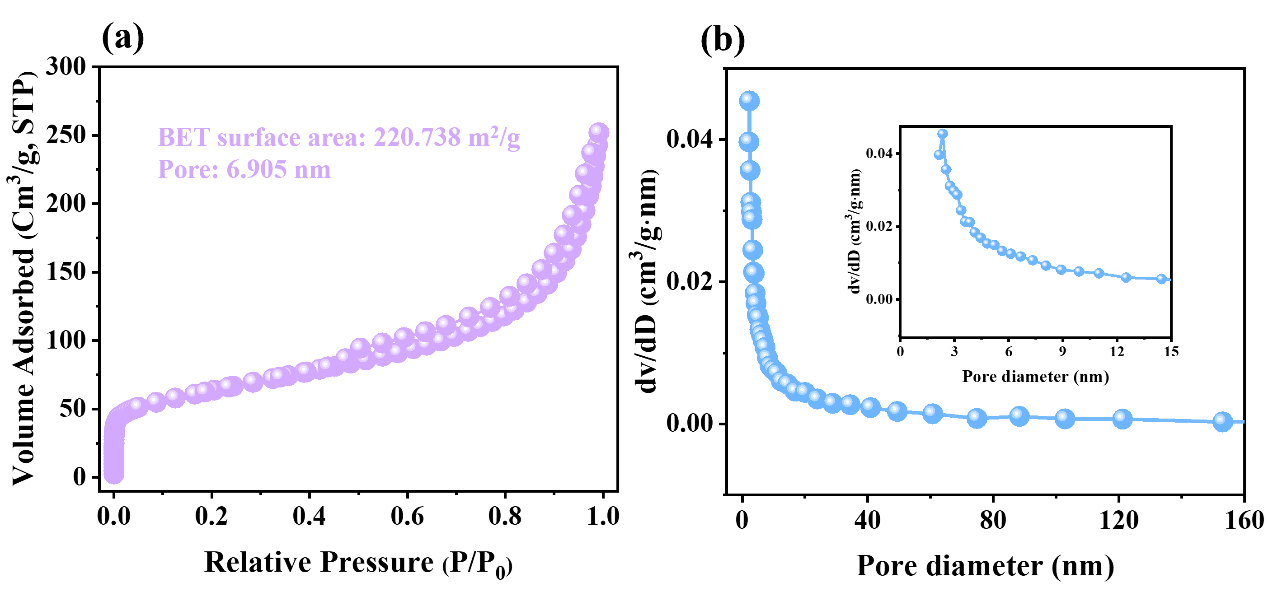


**Figure S8.** (a) Nitrogen adsorption-desorption curve and (b) the corresponding pore-size distribution curves of Co-NC.

NOTE: The isotherm shapes of all the prepared catalysts are type IV with distinct H_3_-type hysteresis lines, indicating a well-defined mesoporous structure^4^. The specific surface areas of Co-NC are similar at 220.738 m^2^/g.


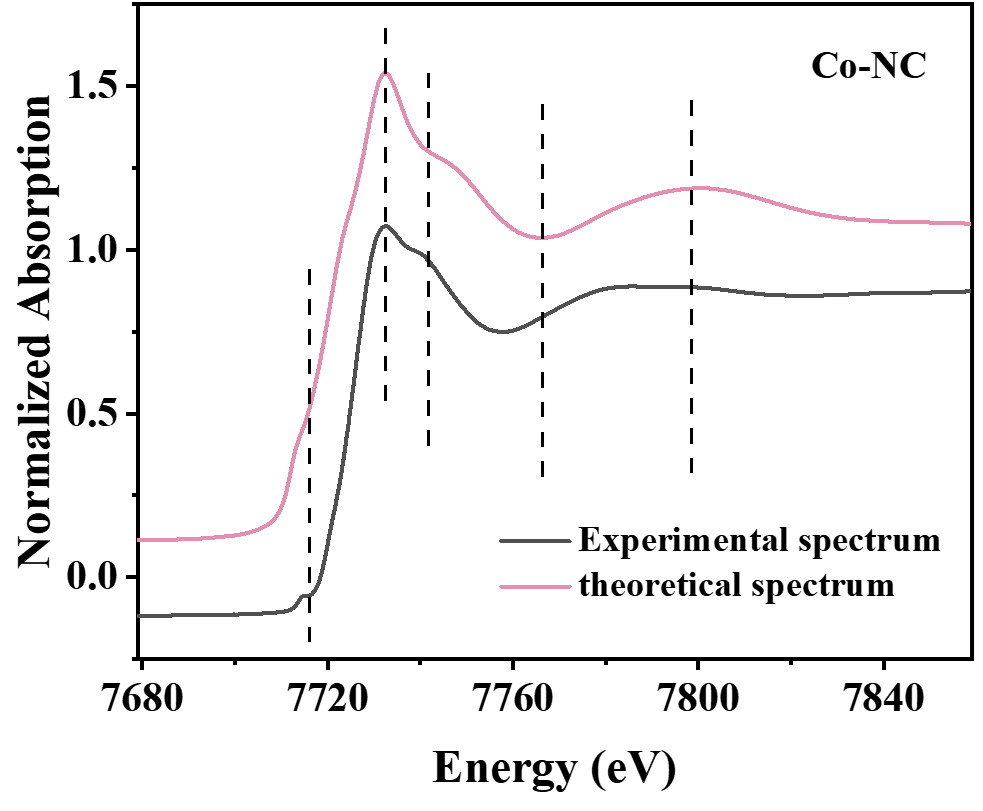


**Figure S9.** CIF-based optimized structural model for EXAFS fitting.


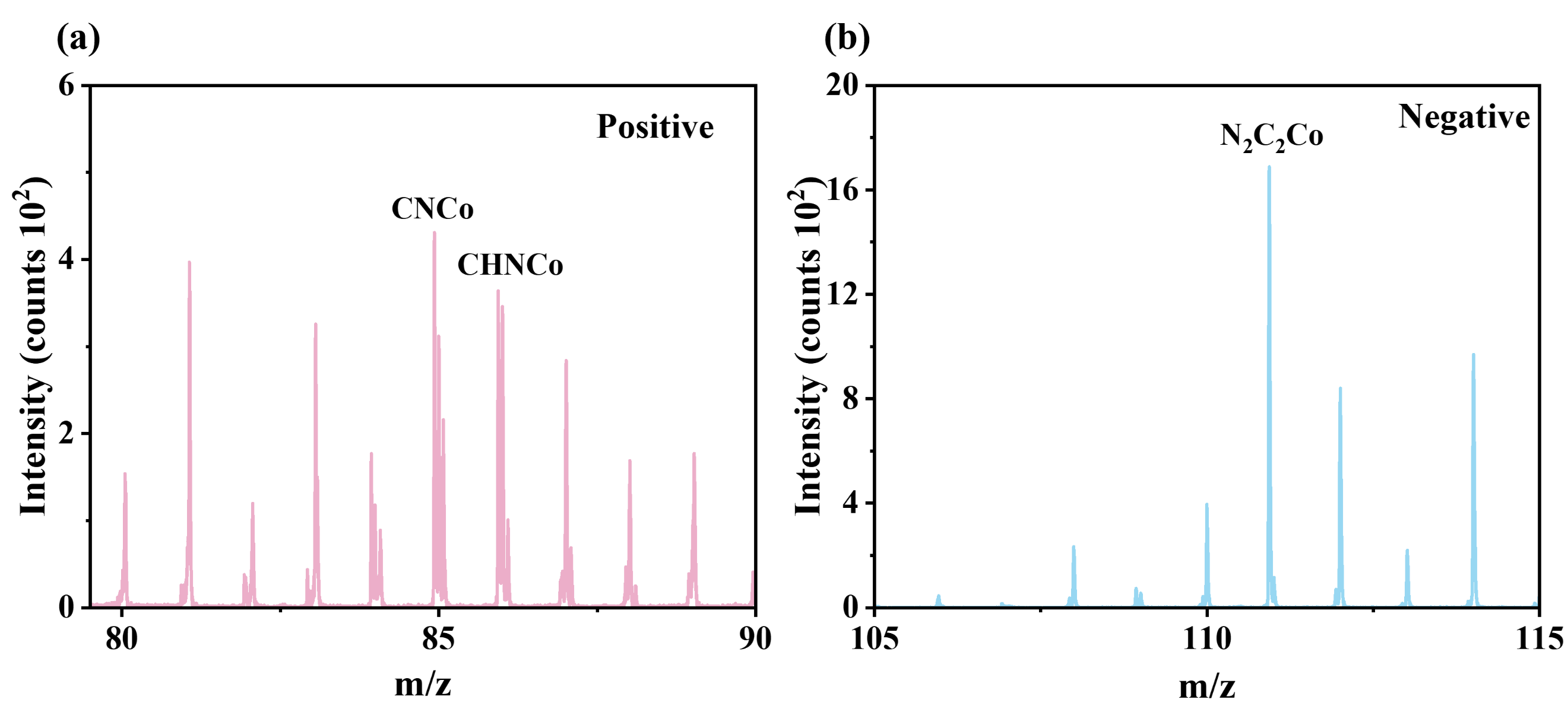


**Figure S10.** TOF-SIMS fragment analysis of Co-NC.


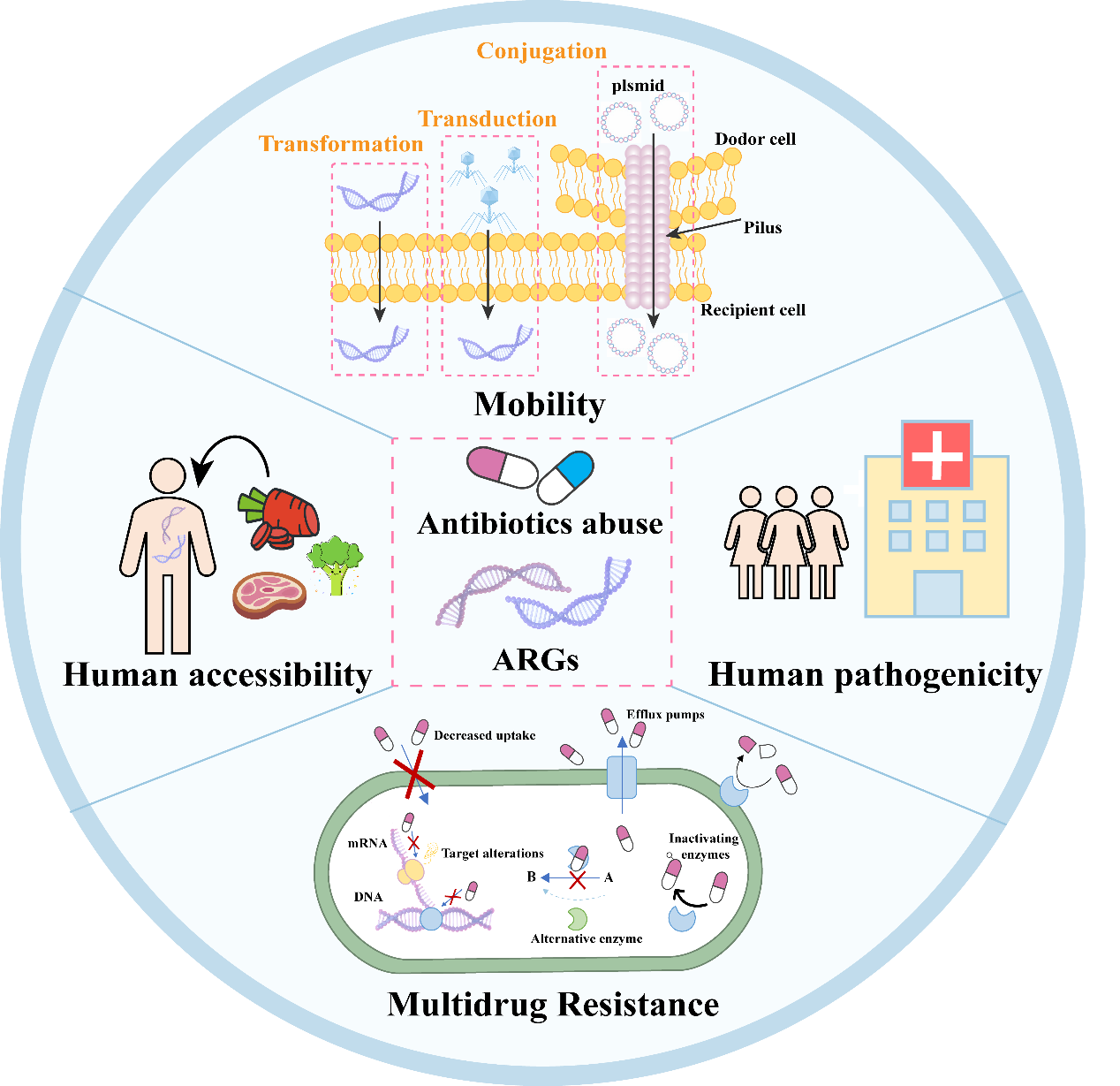


**Figure S11.** The transfer of ARGs and the sources of ARBs resistance.


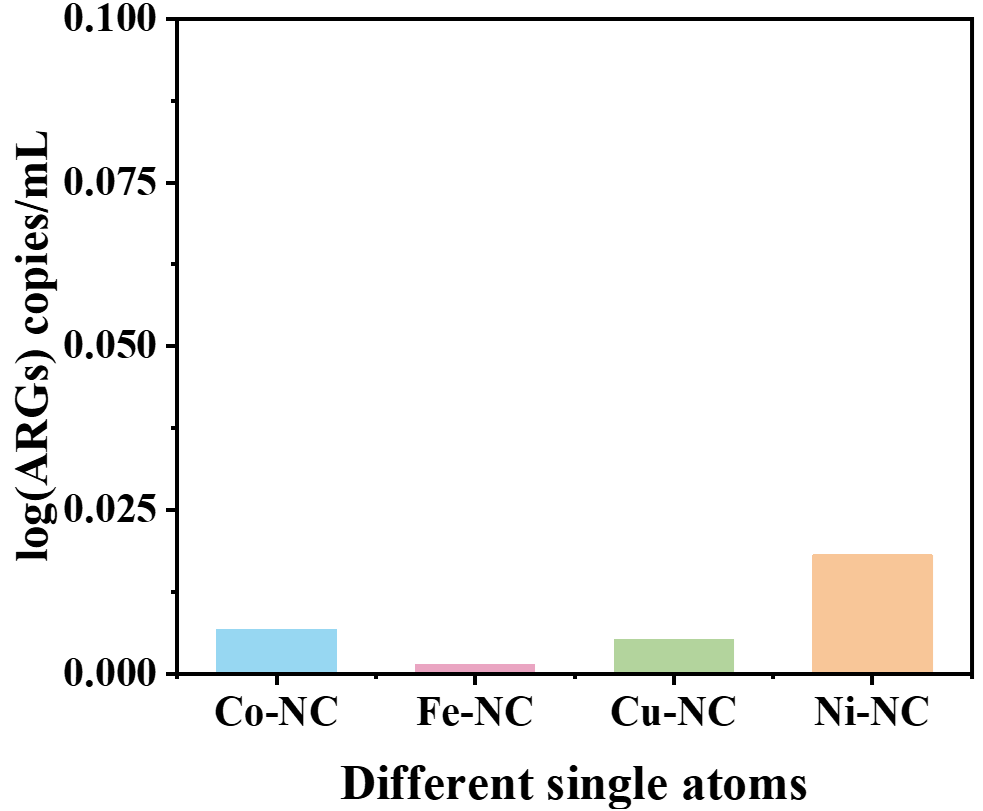


**Figure S12.** The adsorption of ARGs by different single-atom catalyst systems.


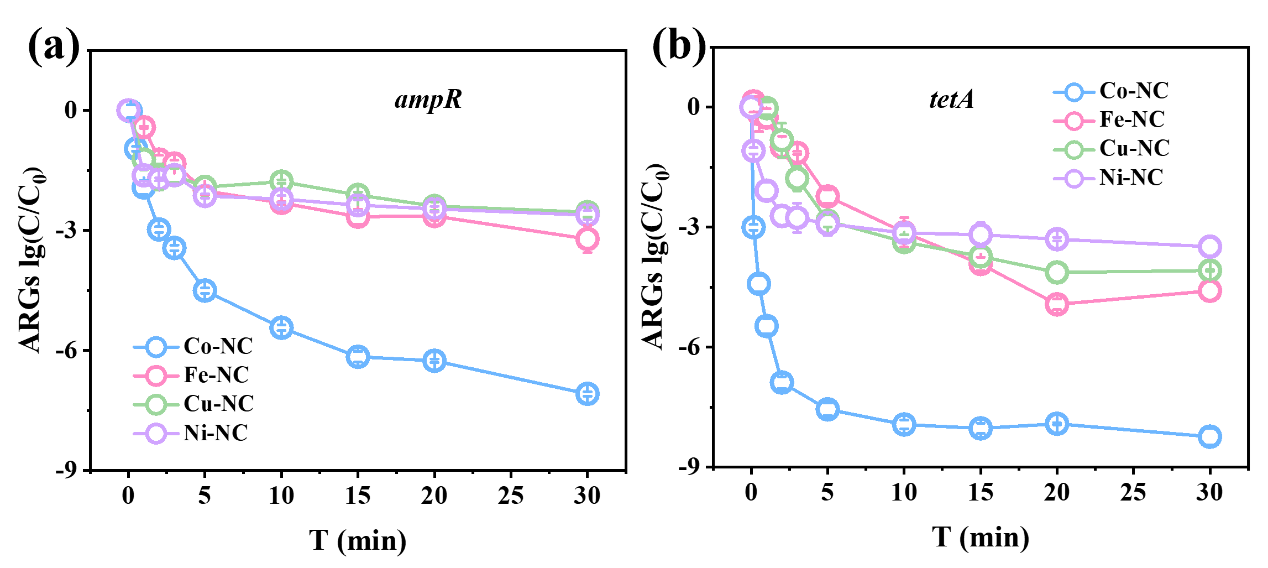


**Figure S13.** Comparison of degradation and first-order rate constant of different ARGs by Co-NC, Fe-NC, Cu-NC and Ni-NC. [Catalyst] = 100 mg/L, [PMS] = 0.5 mM, T = 25 ºC, [ARGs] = 10^12^ copies/mL.


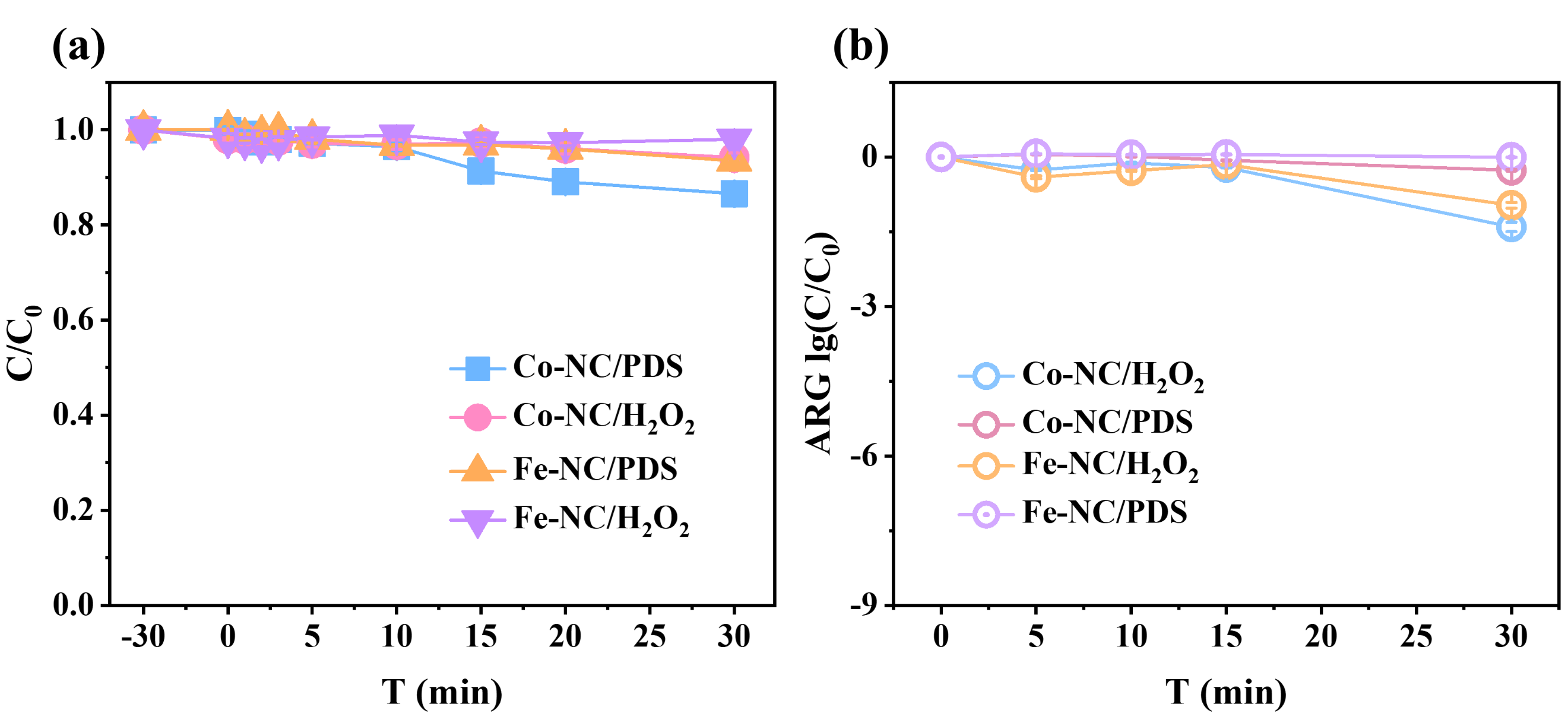


**Figure S14.** Degradation performance of (a) G and (b) ARGs in different activation systems, including Co-NC/PDS, Co-NC/H_2_O_2_, Fe-NC/PDS, and Fe-NC/H_2_O_2_ oxidation systems.


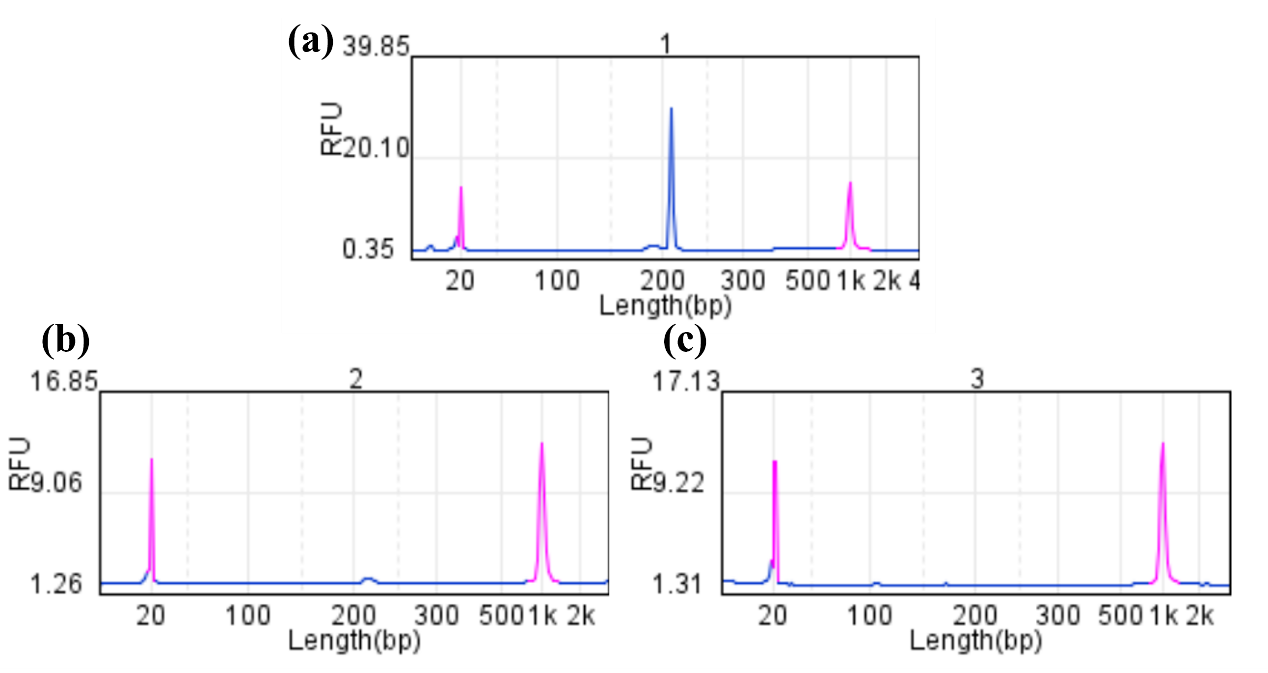


**Figure S15.** Analysis of nucleic acid fragments after degradation of ARGs by Co-NC at 0 min (a), 1 min (b) and 3 min (c), respectively.

Here, nucleic acid fragment analysis and gel electrophoresis experiments are only used for qualitative analysis.


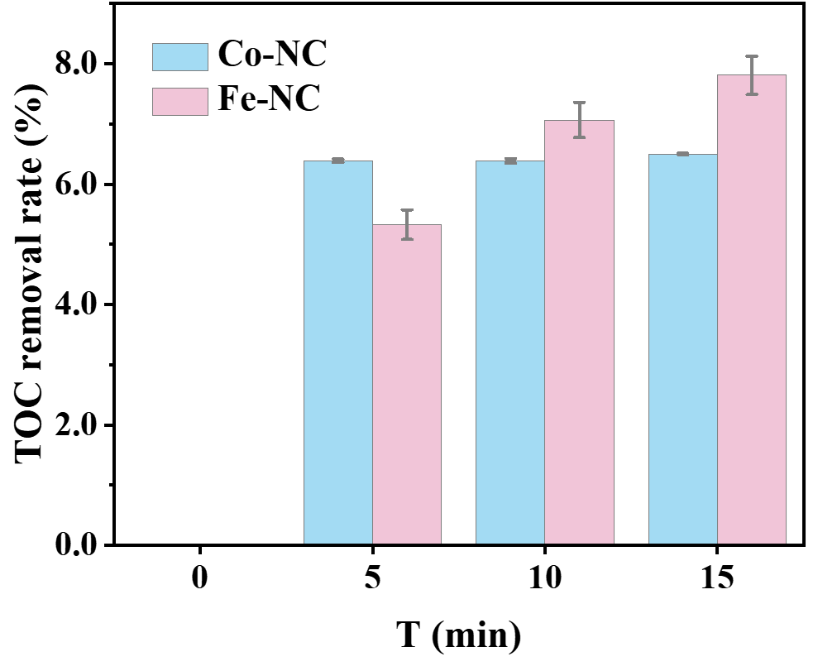


**Figure S16.** Mineralization performance of G in Co-NC/PMS and Fe-NC/PMS oxidation systems.


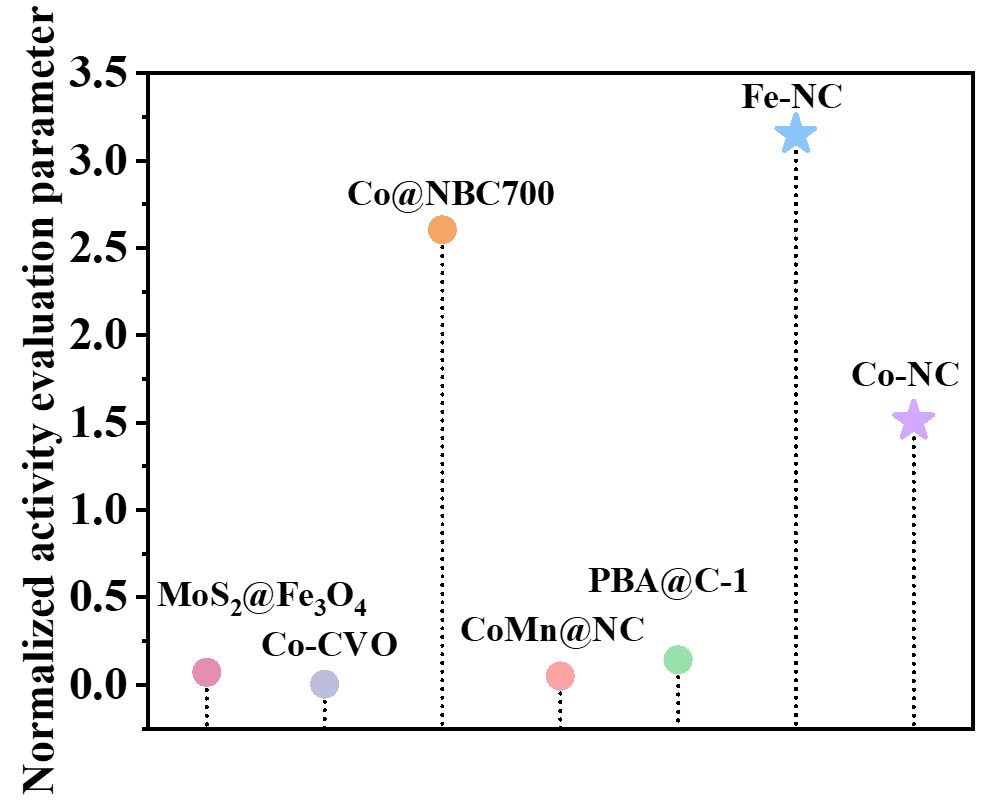


**Figure S17.** Comparison with the normalized activity evaluation parameters of G degradation reported in the literature.

Considering the substantial heterogeneity across previous studies in ARG type, initial gene abundance (10^12^-10^15^ copies·L^-1^), oxidant category, and reaction conditions, direct comparison of ARG removal efficiencies may not accurately reflect the intrinsic catalytic activity of different oxidation systems. Therefore, G was employed as a unified molecular target and a normalized kinetic framework was established under identical conditions to eliminate dosage effects (**Figure S17 and Table S2, Supporting Information**), revealing that both Fe-NC/PMS and Co-NC/PMS exhibit superior intrinsic oxidation activity toward G compared with most reported catalytic oxidation systems.


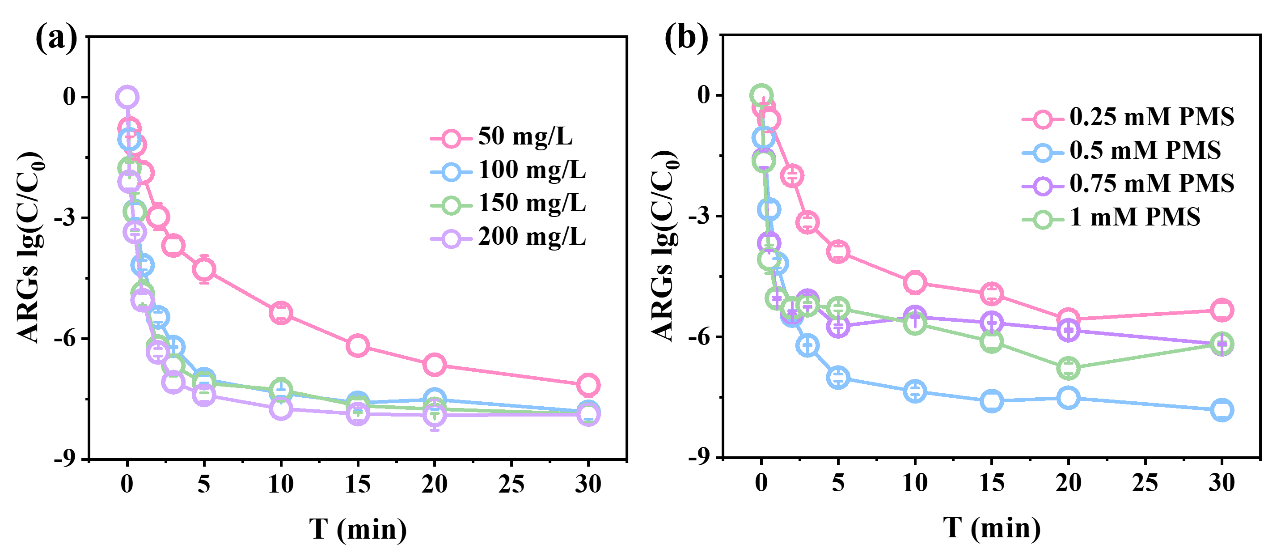


**Figure S18.** Optimization experiments of Co-NC under different conditions: (a) catalyst concentration, (b) PMS concentration. Experimental conditions: T = 25 ºC, [Catalyst] = 100 mg/L, [PMS] = 0.5 mM, pH=7, [ARG] = 10^12^ copies/mL.

When the catalyst concentration was increased from 50 mg/L to 100 mg/L, the catalytic activity of the Co-NC/PMS system was significantly enhanced. However, further increasing the catalyst concentration did not result in a notable improvement in reaction efficiency. This phenomenon can be attributed to the fact that, at 100 mg/L, the number of active sites in the reaction system approaches saturation, and additional catalyst cannot further accelerate the reaction kinetics. Under these conditions, the rate-limiting factor shifts to the concentration of PMS or the pollutant. Similarly, the effect of PMS concentration was investigated, revealing a volcano-type dependence of catalytic efficiency. As PMS concentration increased, ROS generation was greatly enhanced, leading to higher reaction rates. However, when PMS reached 0.5 mM, further addition caused self-quenching of PMS and promoted side reactions between excess PMS and ROS, resulting in a marked decrease in catalytic activity. Therefore, considering both reaction efficiency and economic feasibility, the optimal concentrations for the Co-NC/PMS system were determined to be 100 mg/L for the catalyst and 0.5 mM for PMS.


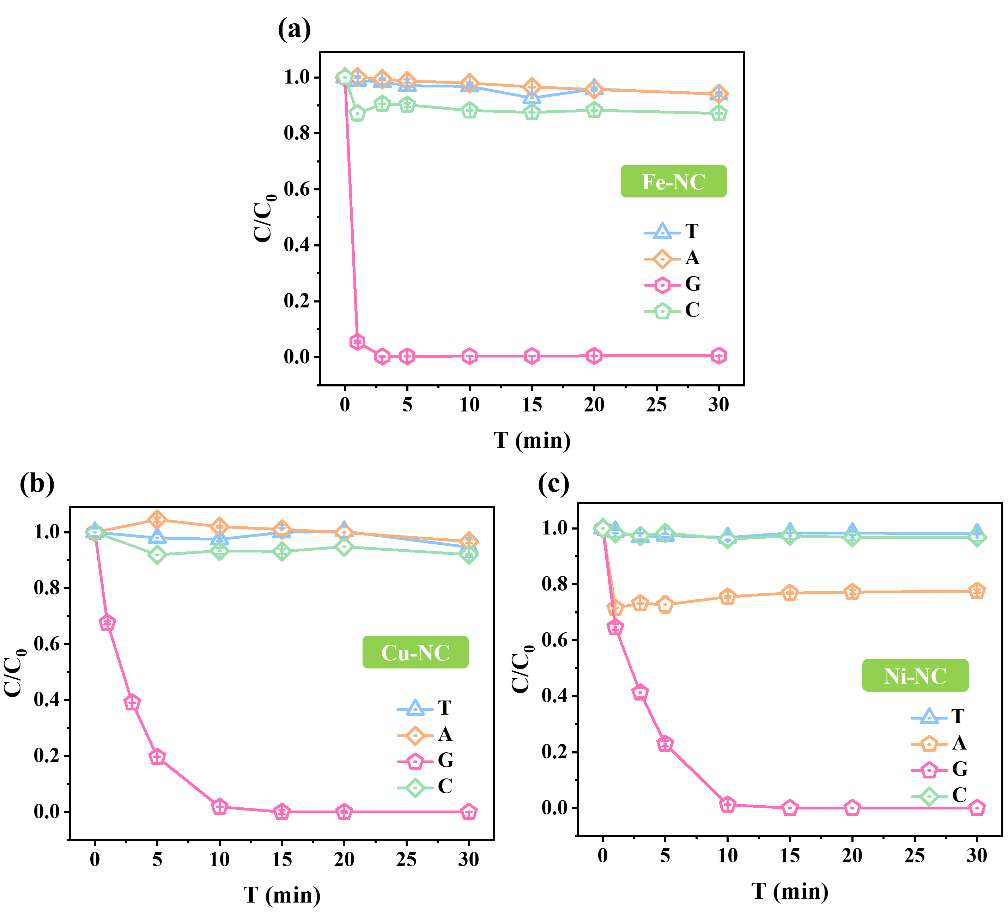


**Figure S19.** Degradation processes and (b) degradation rate of A, T, C and G by Fe-NC (a), Cu-NC (b) and Ni-NC (c). Experimental conditions: [Catalyst] = 100 mg/L, [PMS] = 0.5 mM, [Pollutant] = 50 µM, T = 25 ºC.


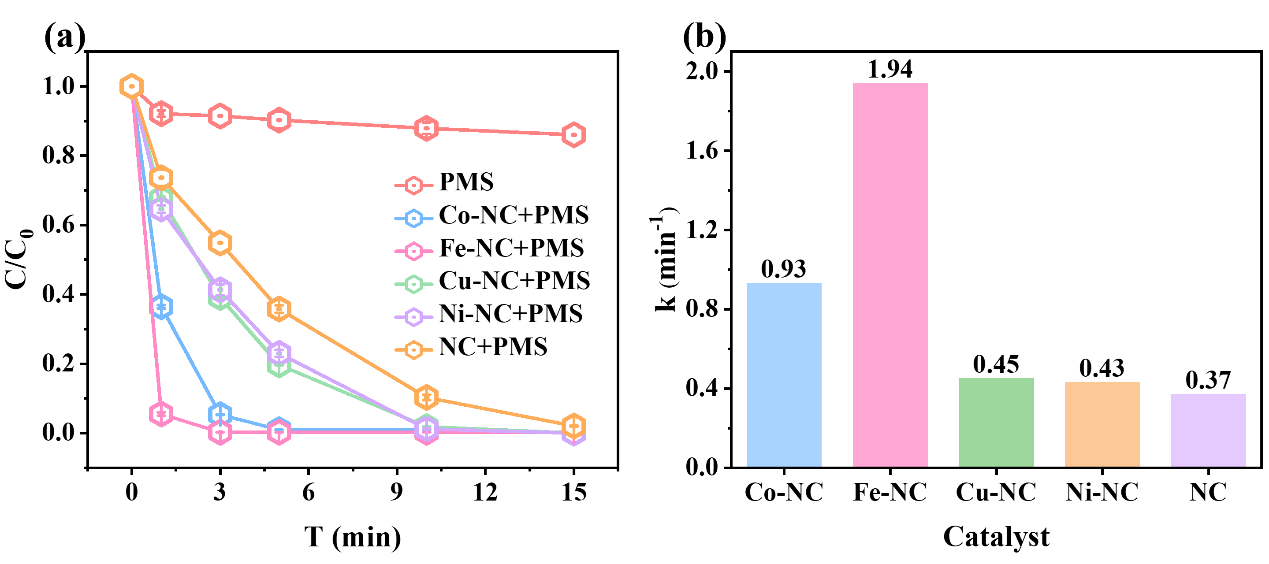


**Figure S20.** (a) Comparison of degradation of base G in different systems. (b) Comparison of apparent first-order rate constant of G by different catalysts. Experimental conditions: [Catalyst] = 100 mg/L, [PMS] = 0.5 mM, [Pollutant] = 50 µM, T = 25 ºC.


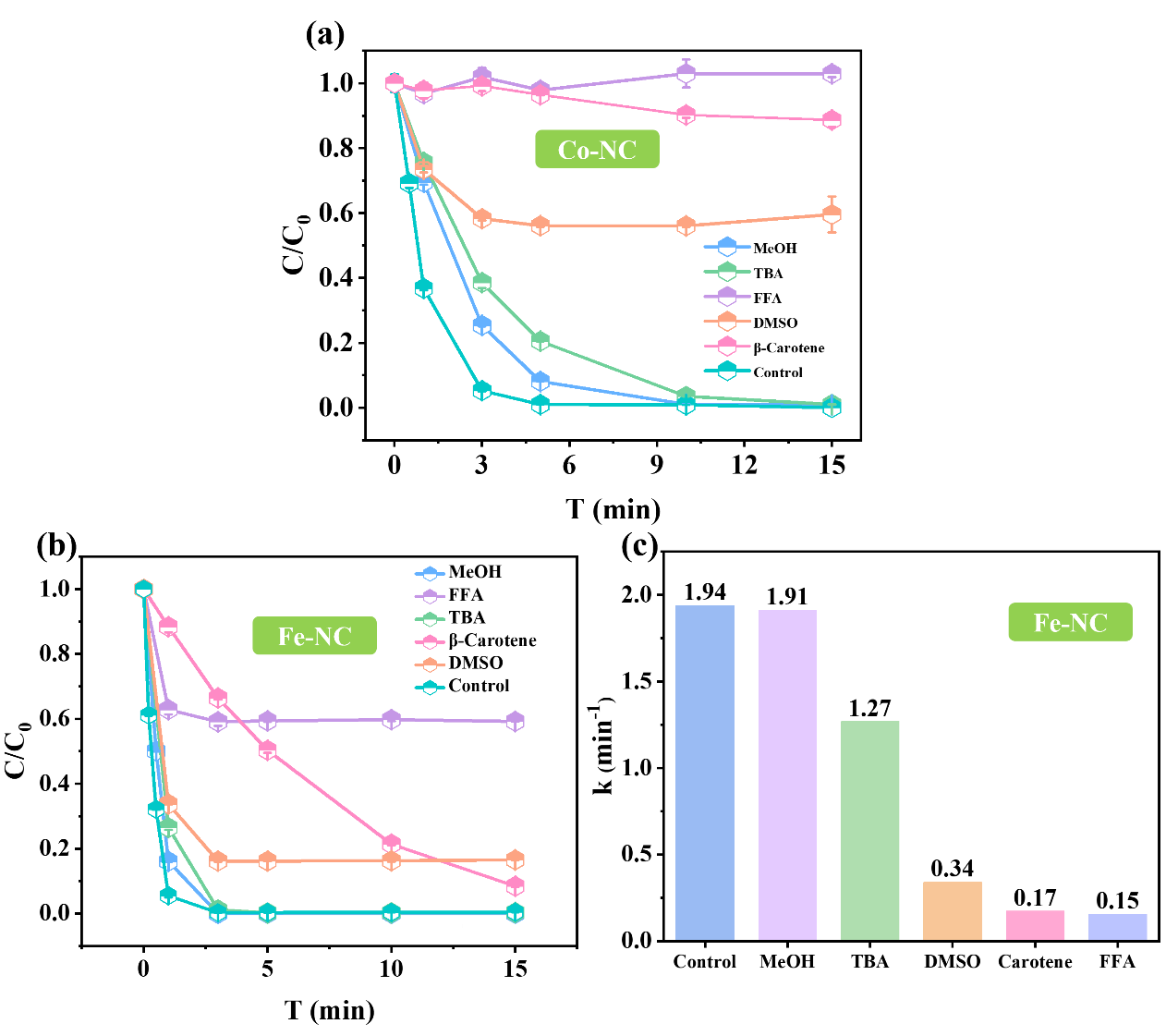


**Figure S21.** (a) Quenching experiments of the Co-NC/PMS system. (b) The effect of different quenchers on the degradation of G in Fe-NC system and (c) comparison of apparent first-order rate constant. Experimental conditions: [Carotene] = 5 mM, [MeOH] = [TBA] = [FFA] = [DMSO] = 100 mM, [Catalyst] = 100 mg/L, [PMS] = 0.5 mM, [G] = 50 µM, T = 25 ºC.


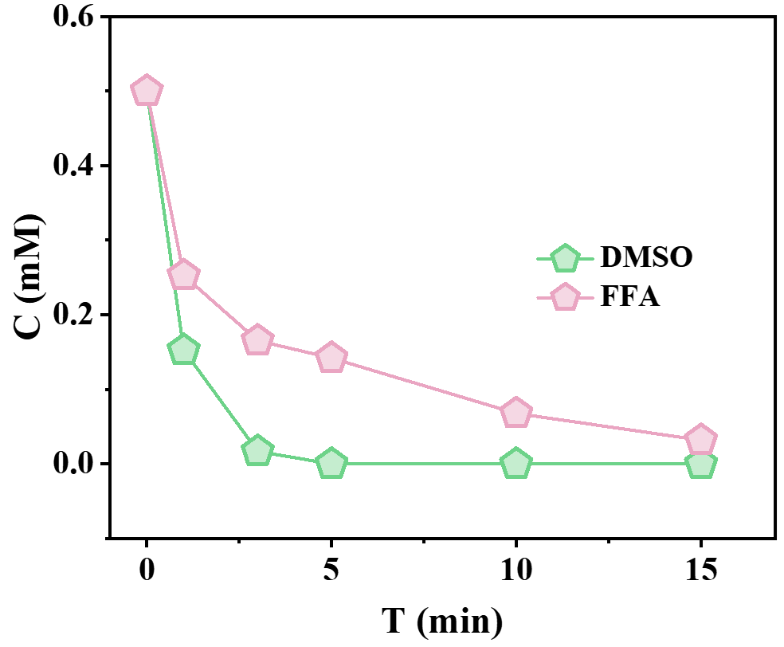


**Figure S22.** Consumption of PMS by different quenchers. Experimental conditions: [FFA] = [DMSO] = 100 mM, [Catalyst] = 100 mg/L, [PMS] = 0.5 mM, T = 25 ºC.


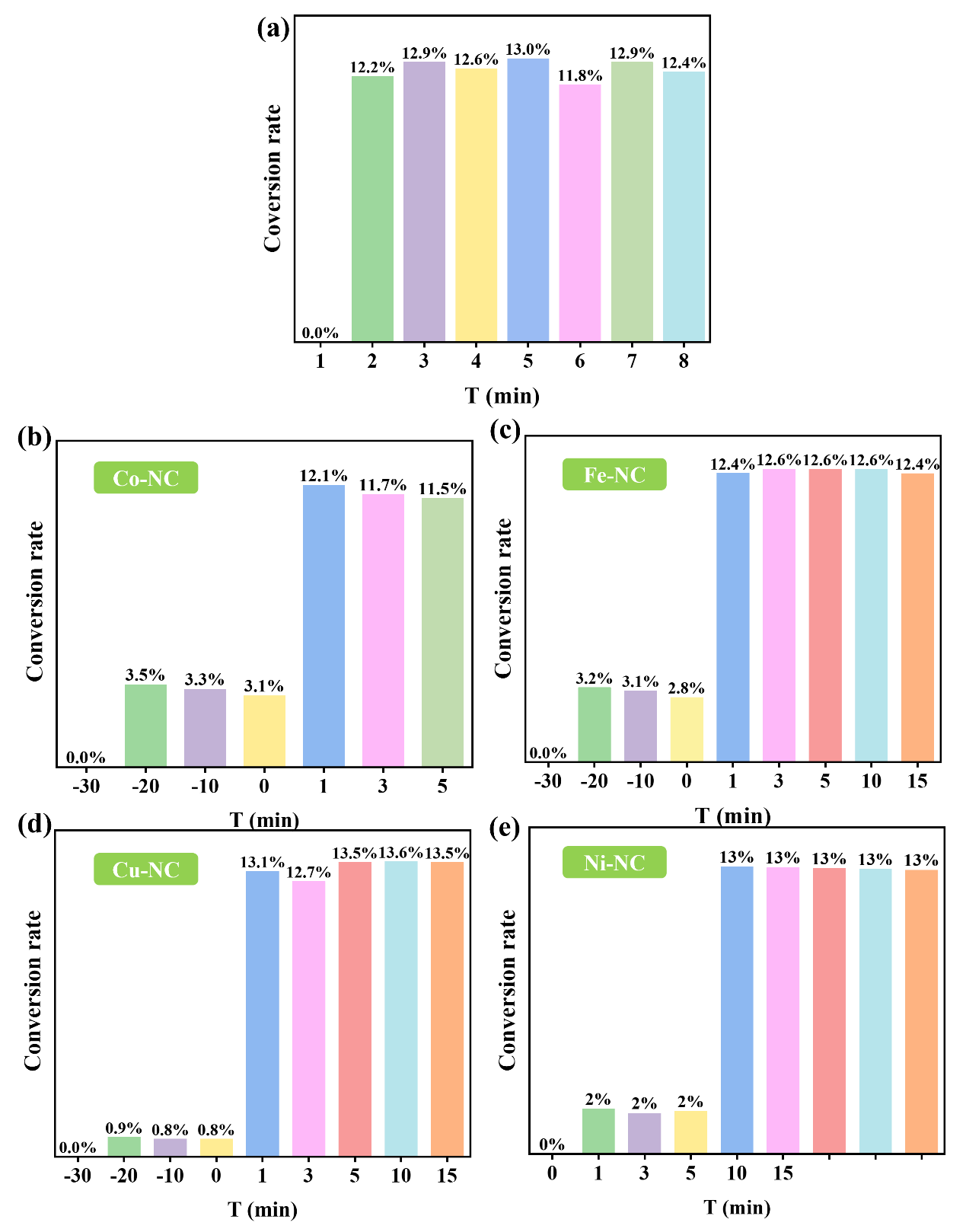


**Figure S23.** (a) Single PMS conversion experiment from PMSO to PMSO_2_. Experimental study on the conversion of PMSO to PMSO_2_ in Co-NC (b), Fe-NC (c), Cu-NC (d), Ni-NC (e) systems. Experimental conditions: [PMSO] = 100 mM, [Catalyst] = 100 mg/L, [PMS] = 0.5 mM, T = 25 ºC.

**
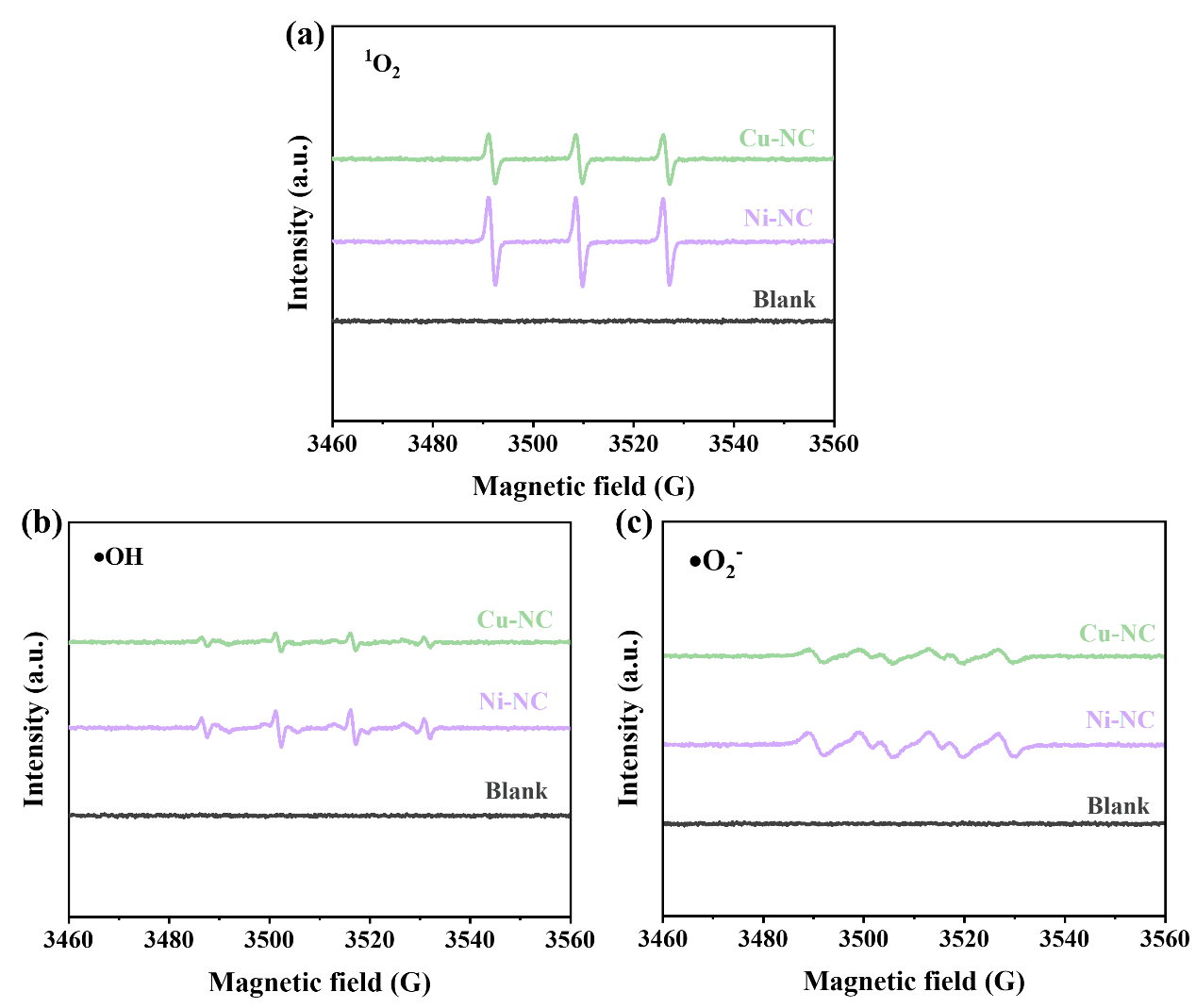
**

**Figure S24.** (a) EPR spectra of the different systems captured by TEMP. (b-c) EPR spectra of the different systems captured by DMPO.


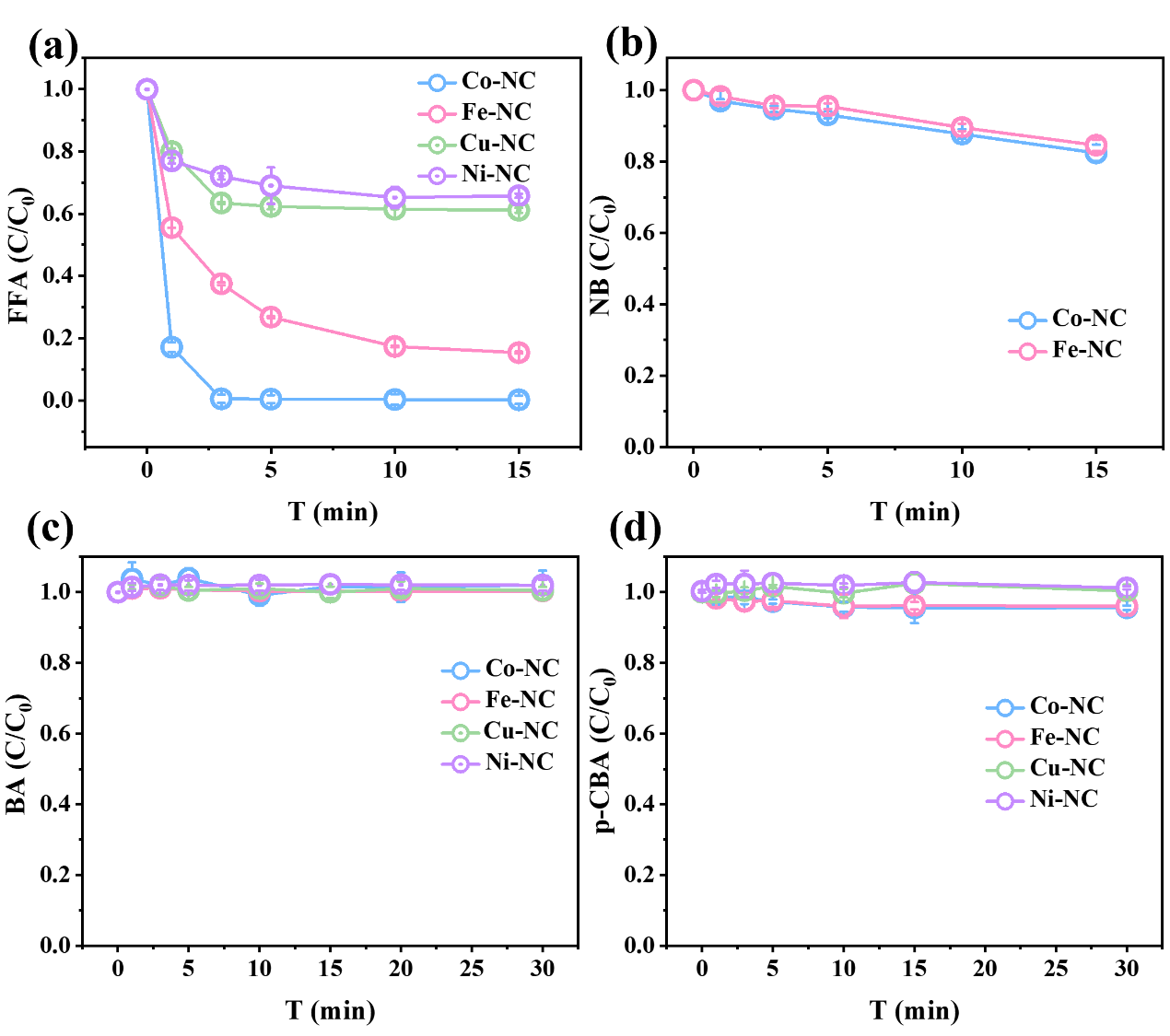


**Figure S25.** Degradation efficiency of (a) FFA, (b) NB (c) BA and (d) p-CBA in different catalytic systems. Reaction conditions: [BA] = [FFA] = [NB] = [p-CBA] = 10 µM, [PMS] = 0.5 mM, [Catalyst] = 100 mg/L.


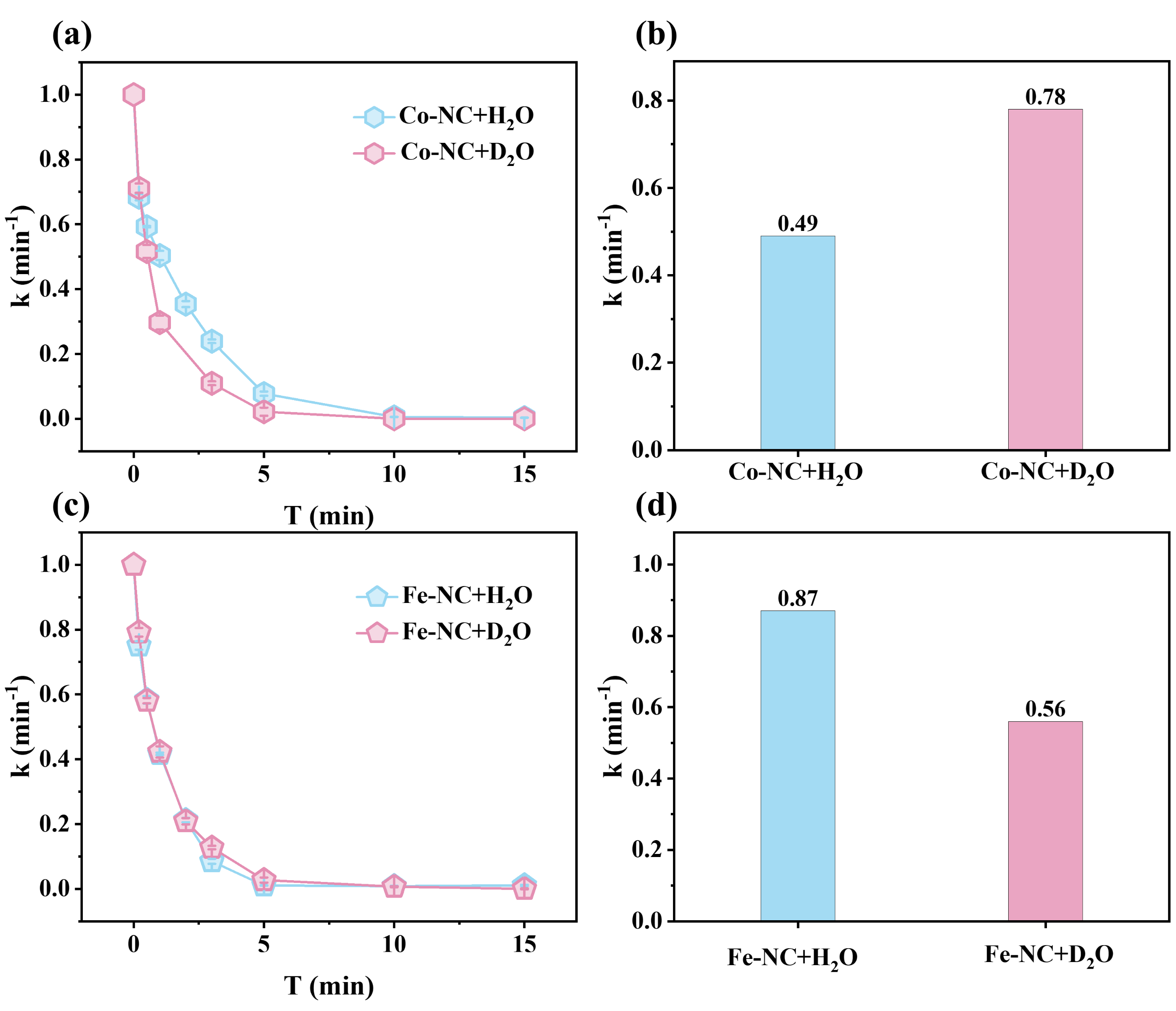


**Figure S26.** Degradation efficiency of ARGs in Co-NC/PMS (a-b) and Fe-NC/PMS (c-d) systems with D_2_O and H_2_O as the reaction solvents, respectively.


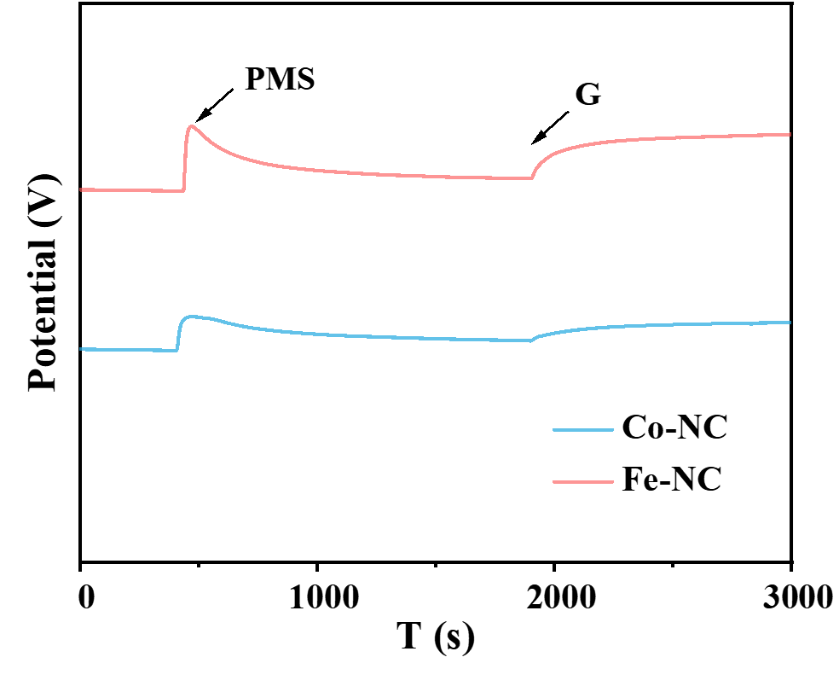


**Figure S27.** Open-circuit potential spectra of Co-NC and Fe-NC systems.


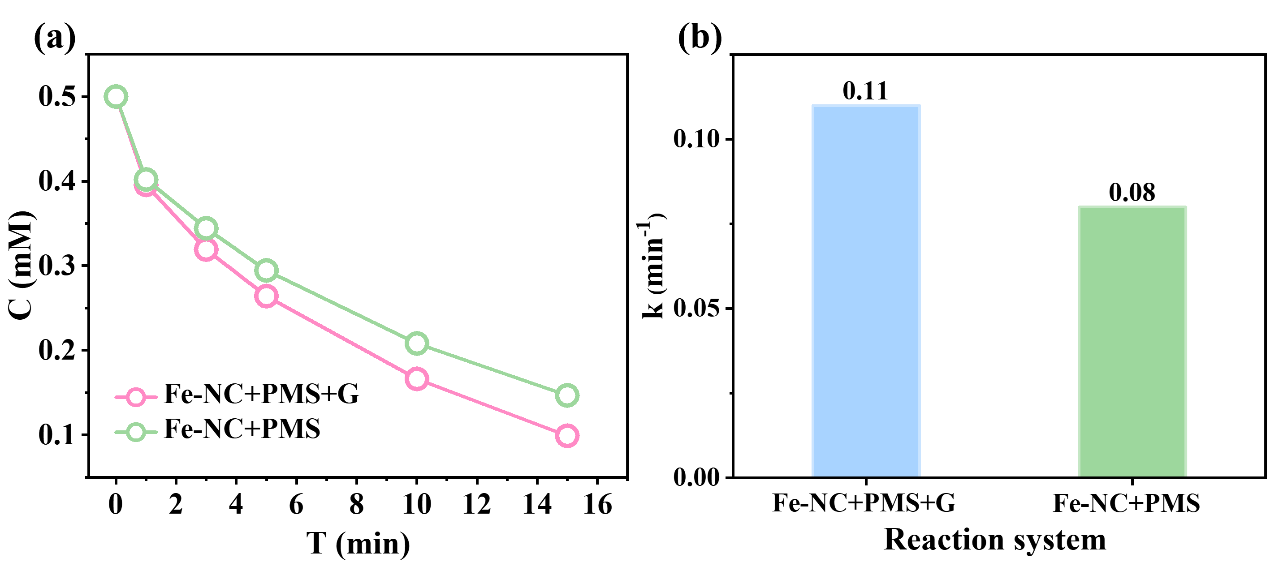


**Figure S28.** The change of PMS concentration (a) and the apparent first-order rate constant of PMS consumption (b) in the reaction systems of Fe-NC/PMS/G and Fe-NC/PMS. Experimental conditions: [Catalyst] = 100 mg/L, [PMS] = 0.5 mM, [G] = 50 µM, T = 25 ºC.


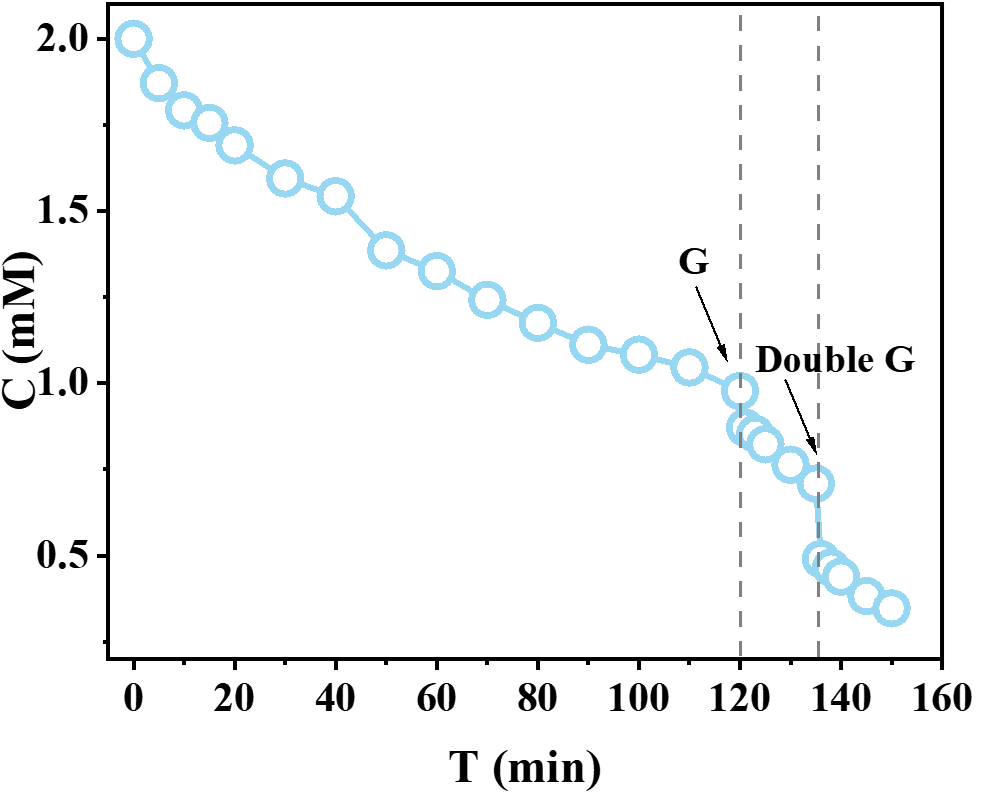


**Figure S29.** PMS consumption process after introducing G at different reaction stages in the Fe-NC system.


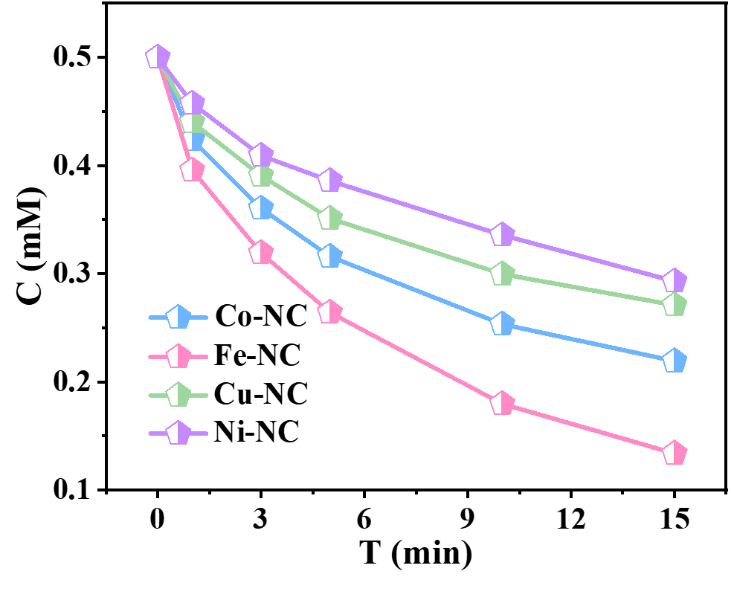


**Figure S30.** Consumption of PMS by different single-atom systems.


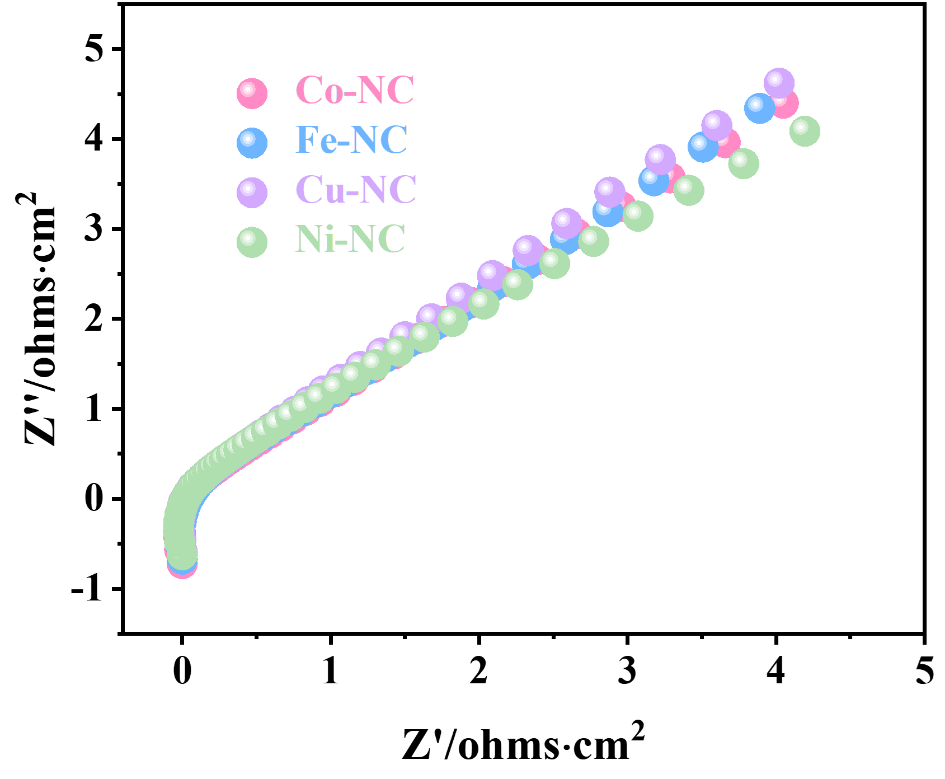


**Figure S31.** Electrochemical impedance spectroscopy of different single-atom catalysts.


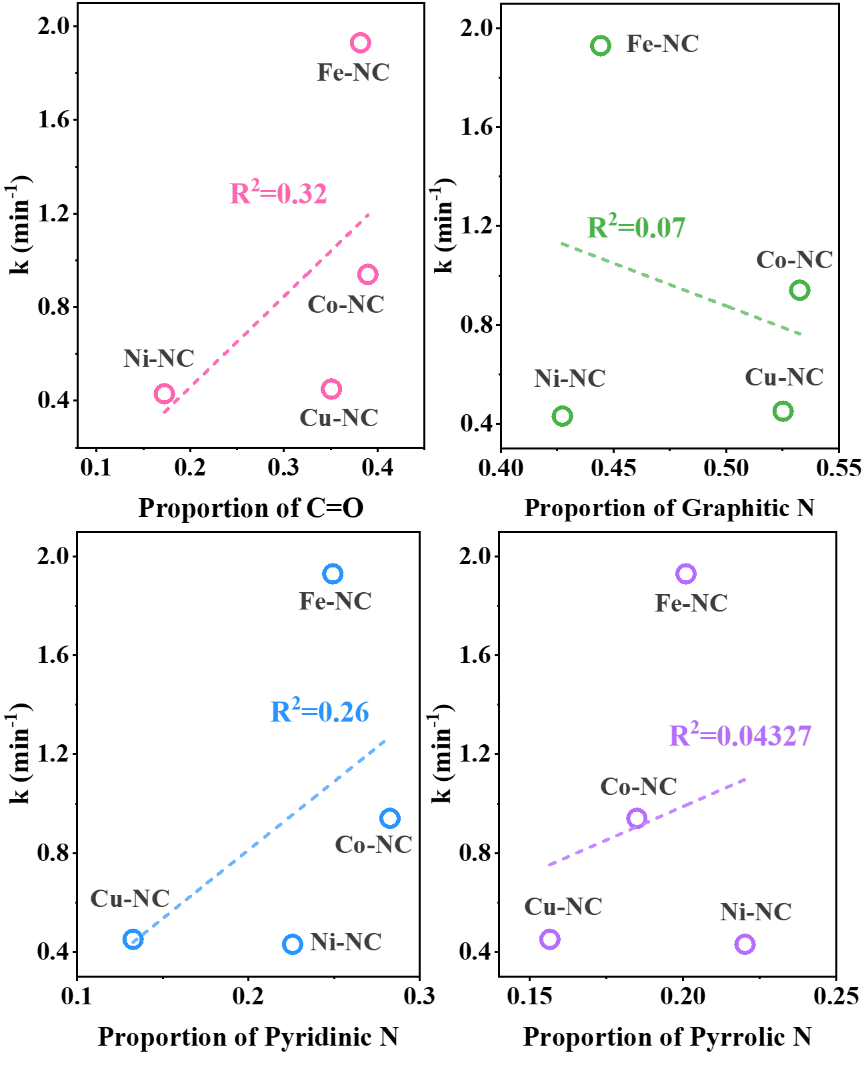


**Figure S32.** The correlation between the first-order rate constant of G degradation in different catalysis system and the proportion of different chemical species.


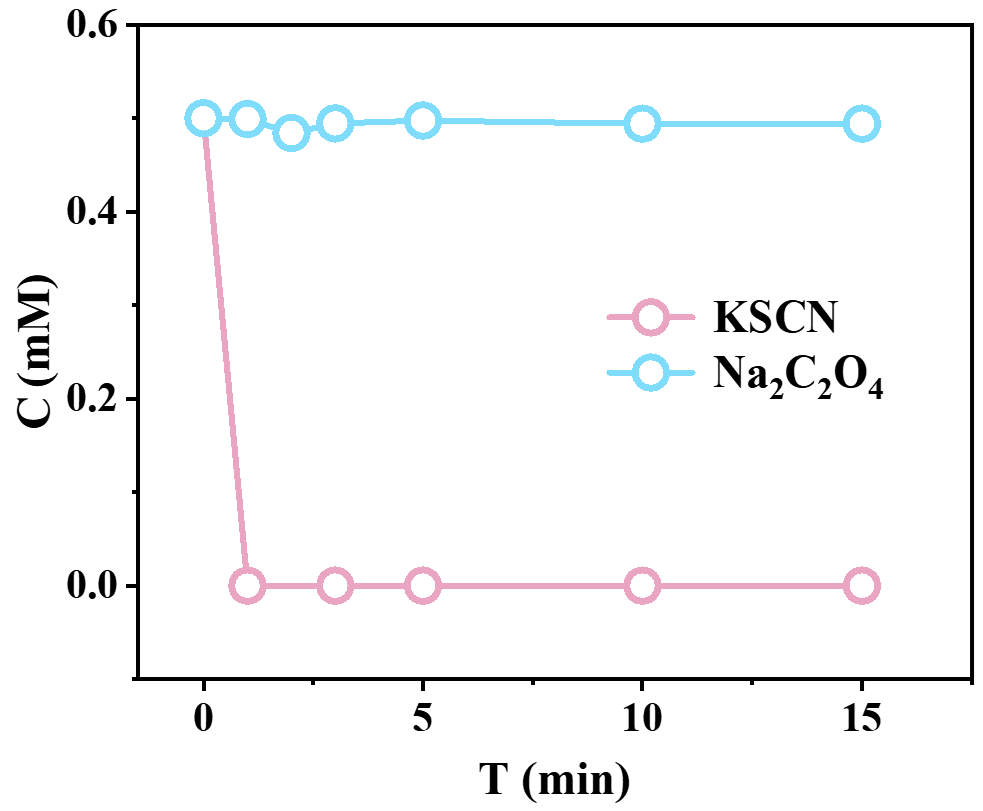


**Figure S33.** Consumption of PMS in the presence of Na_2_C_2_O_4_ and potassium thiocyanate KSCN.


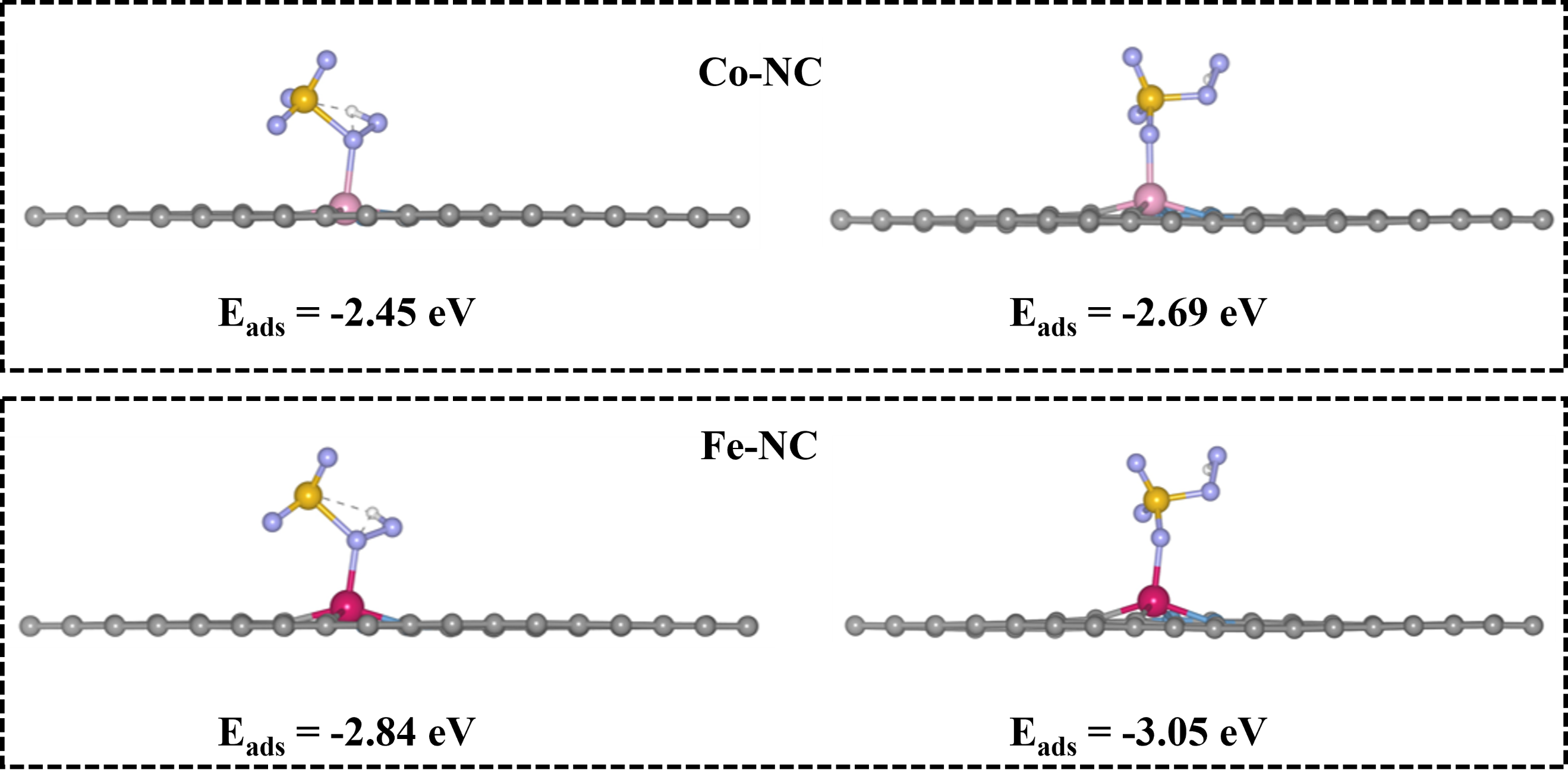


**Figure S34.** Optimized configurations of PMS adsorbed on Co-NC and Fe-NC and the corresponding O-O bond length and adsorption energy.


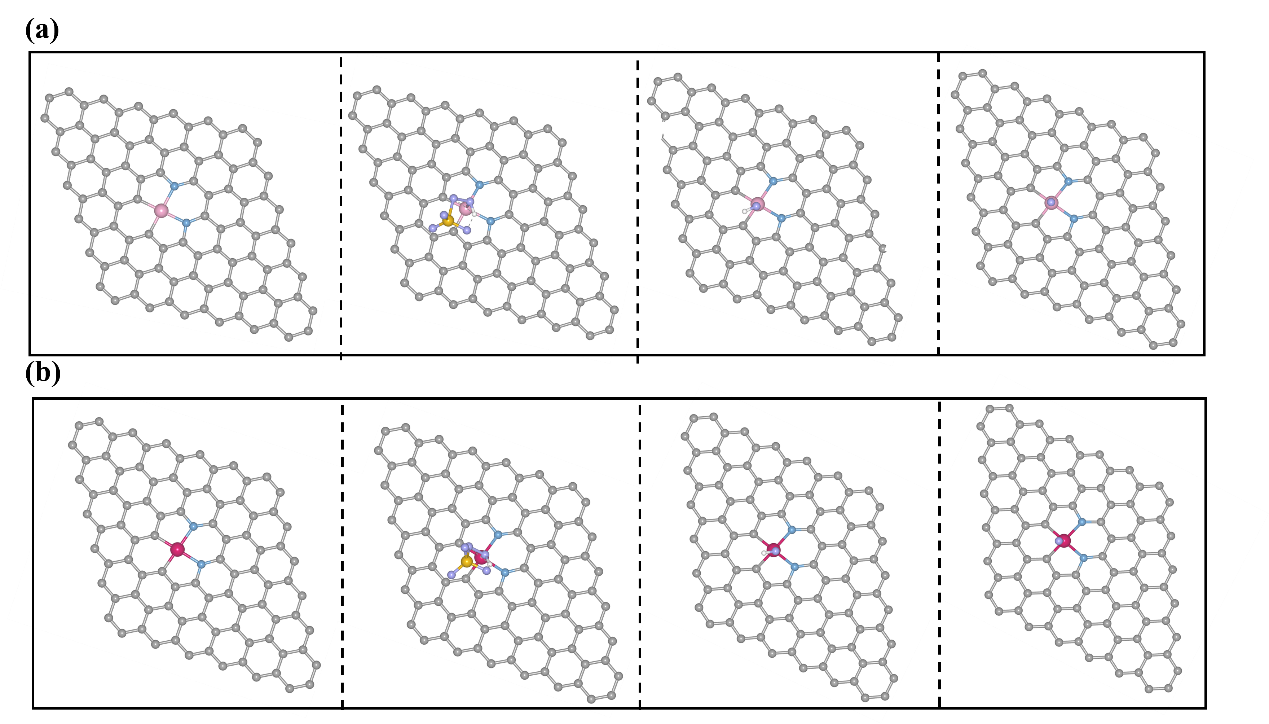


**Figure S35.** The intermediate adsorption and reaction structure of Co-NC (a) and Fe-NC (b).


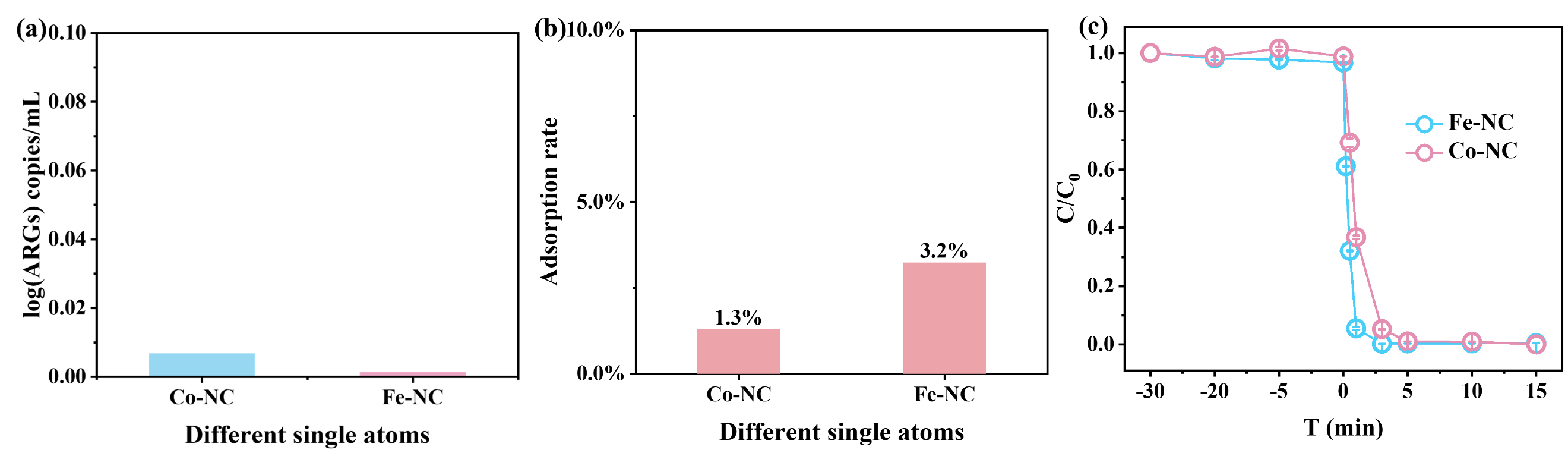


**Figure S36.** The adsorption of *bla*_TEM-1_ (a) and G (b) by Co-NC and Fe-NC. (c) Adsorption and degradation of G by Co-NC and Fe-NC.


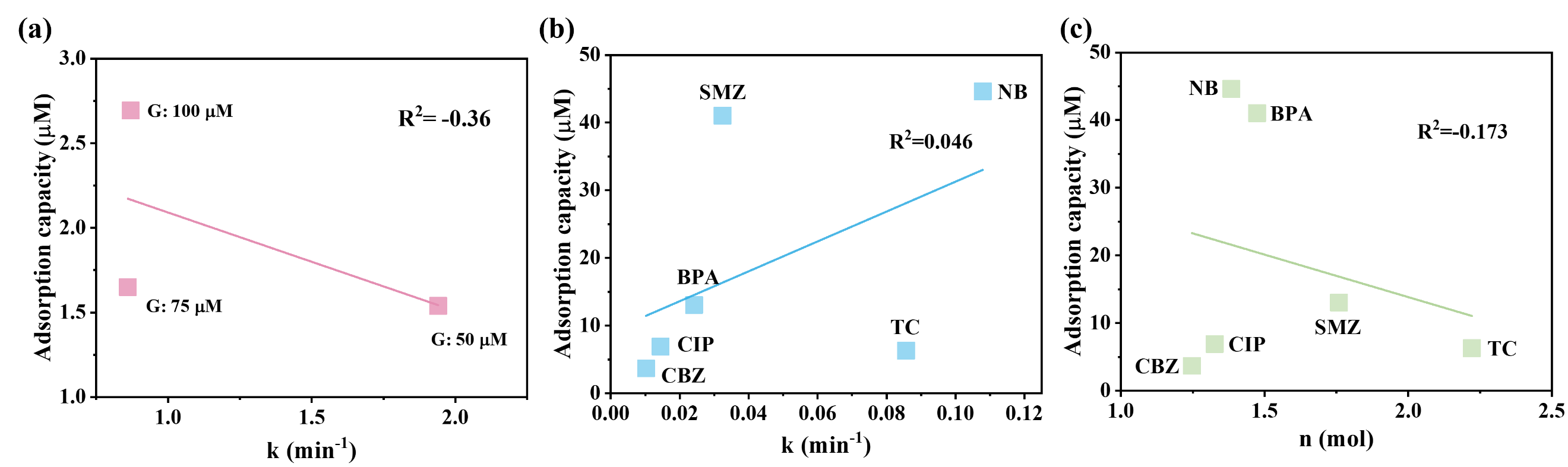


**Figure S37.** (a) The correlation between adsorption capacity and degradation efficiency in the Fe-NC/PMS system. (b) The correlation between adsorption capacity and degradation efficiency for different pollutants. (c) The correlation between adsorption capacity and removal efficiency for different pollutants.


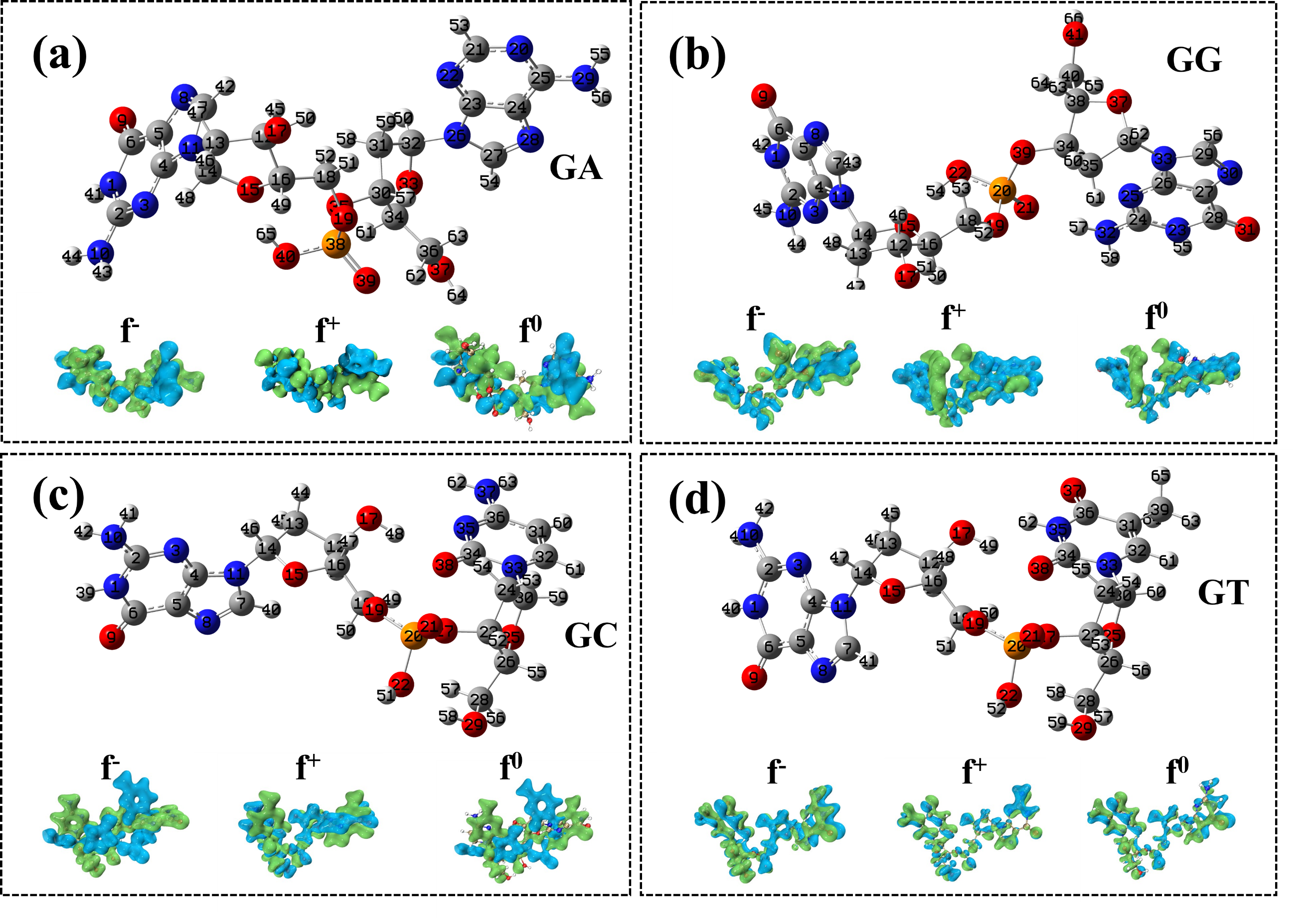


**Figure S38.** Fukui function values of GA (a), GG (b), GC (c) and GT (d).


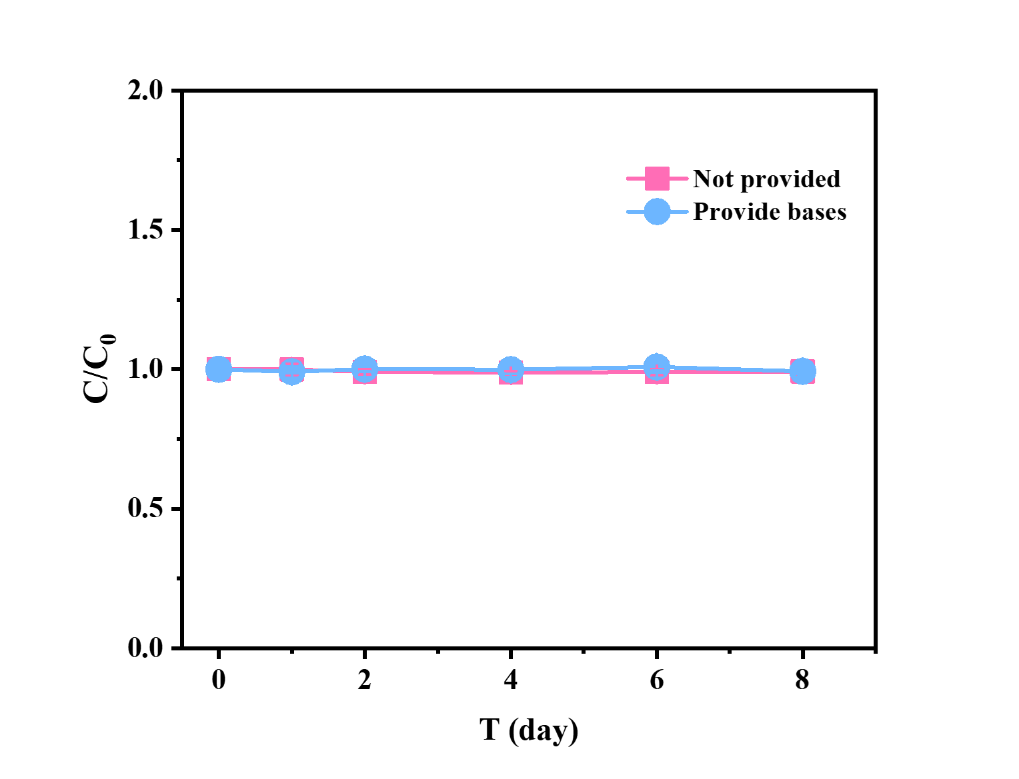


**Figure S39.** Regeneration of ARG after degradation.

In order to investigate whether ARG will regenerate after degradation with the provision of base A, T, C and G, it can be seen from Figure S31 that ARG will not regenerate after degradation, regardless of whether the base is provided or not.


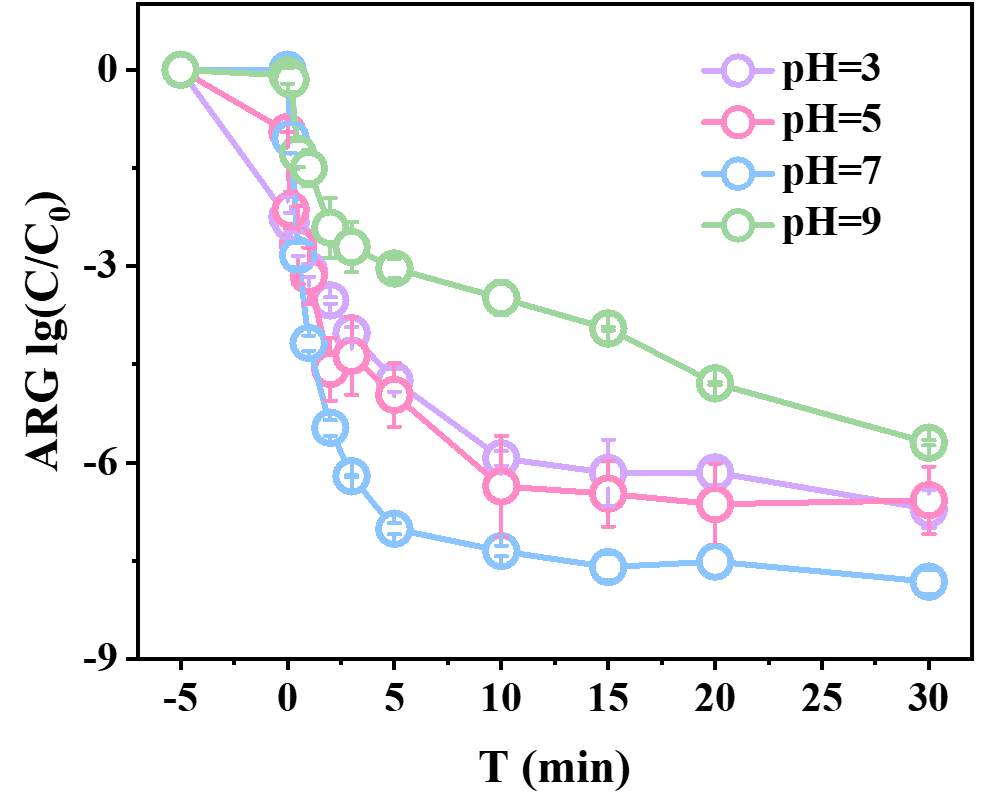


**Figure S40.** The degradation of *bla*_TEM-1_ by the Co-NC/PMS system after 30 min of reaction at different pH.


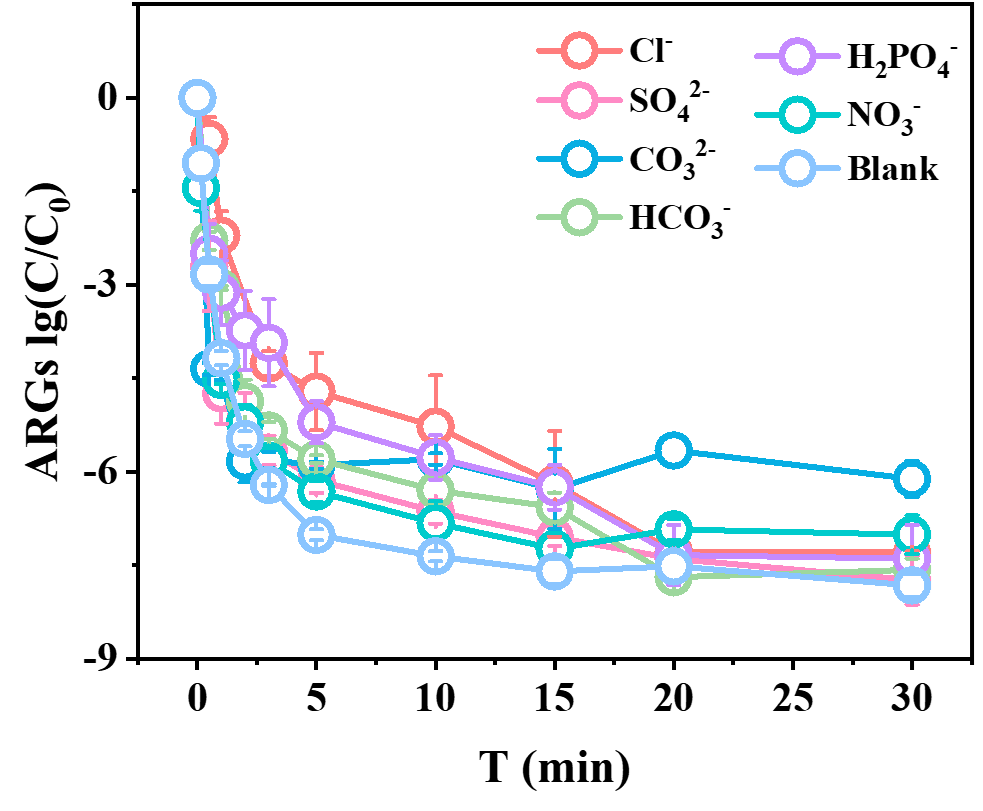


**Figure S41.** Effects of different inorganic anions on degradation of ARGs by Co-NC activated PMS. Experimental conditions: [Catalyst] = 100 mg/L, [PMS] = 0.5 mM, T = 25 ºC, [ARG] = 10^12^ copies/mL, [Ionic] = 50 mg/L.


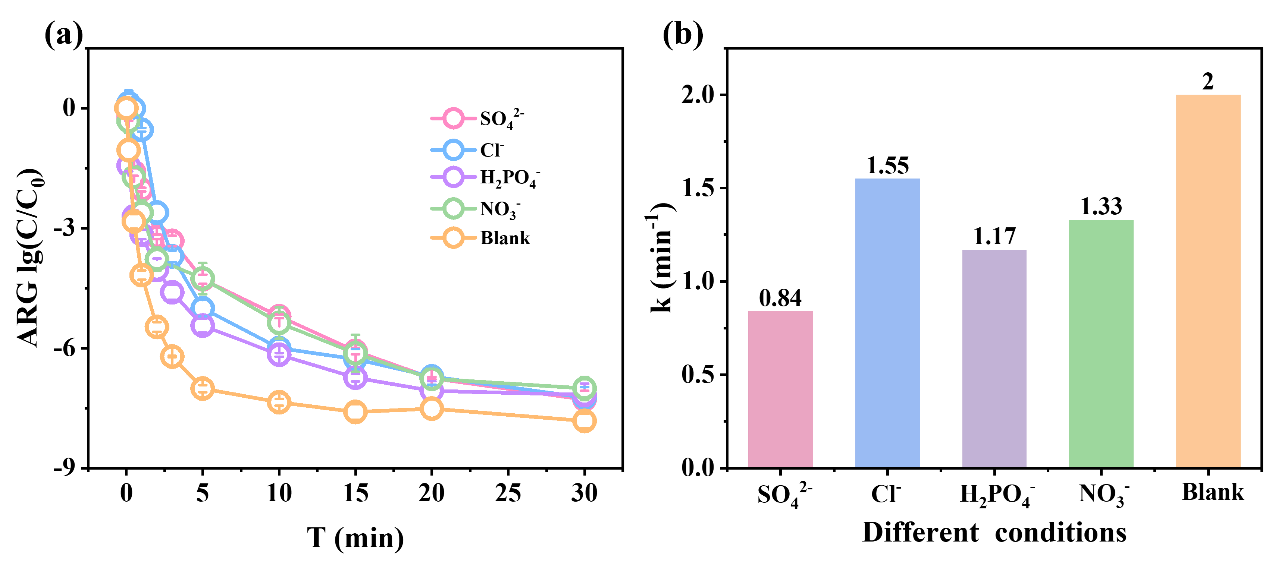


**Figure S42.** The degradation of *bla*_TEM-1_ by the Co-NC/PMS system after 30 min of reaction at different ions concentrations. Experimental conditions: [Catalyst] = 100 mg/L, [PMS] = 0.5 mM, [Ions] = 300 mg/L, [ARG] = 10^12^ copies/mL, T = 25 ºC.


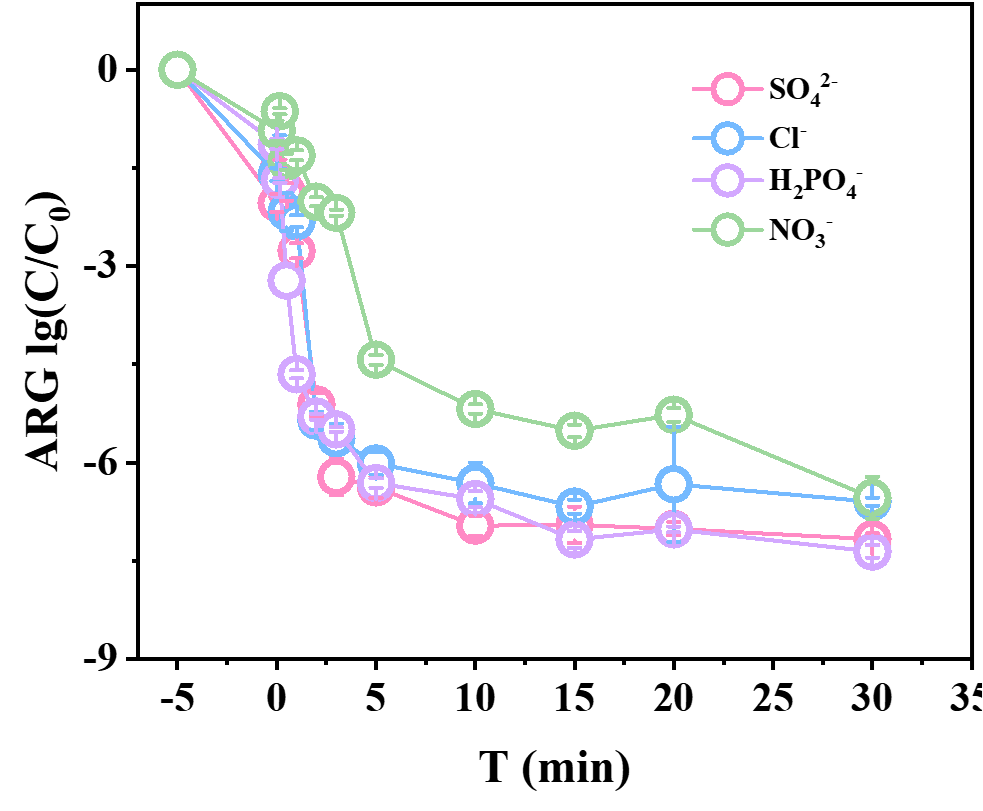


**Figure S43.** The degradation of *bla*_TEM-1_ by the Co-NC/PMS system after 30 min of reaction at different ions concentrations. Experimental conditions: [Catalyst] = 100 mg/L, [PMS] = 0.5 mM, [Ions] = 3000 mg/L, [ARG] = 10^12^ copies/mL, T = 25 ºC.


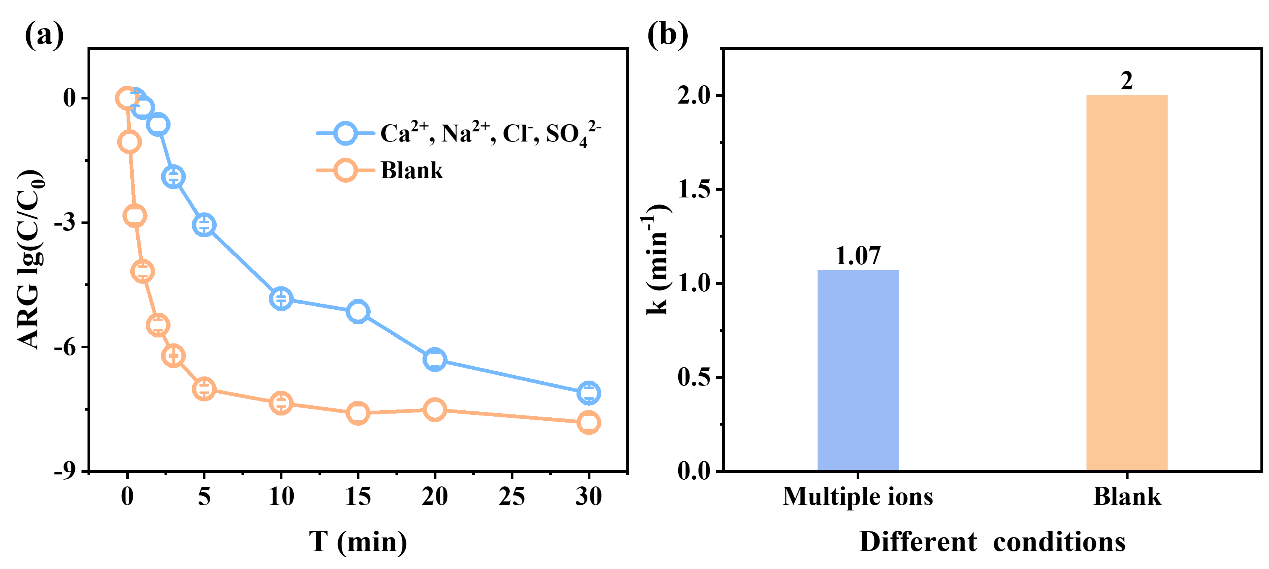


**Figure S44.** The degradation of *bla*_TEM-1_ by the Co-NC/PMS system after 30 min of reaction at the conditions of Ca^2+^, Na^+^, Cl^-^, SO_4_^2-^. Experimental conditions: [Catalyst] = 100 mg/L, [PMS] = 0.5 mM, [Ions] = 300 mg/L, [ARG] = 10^12^ copies/mL, T = 25 ºC.


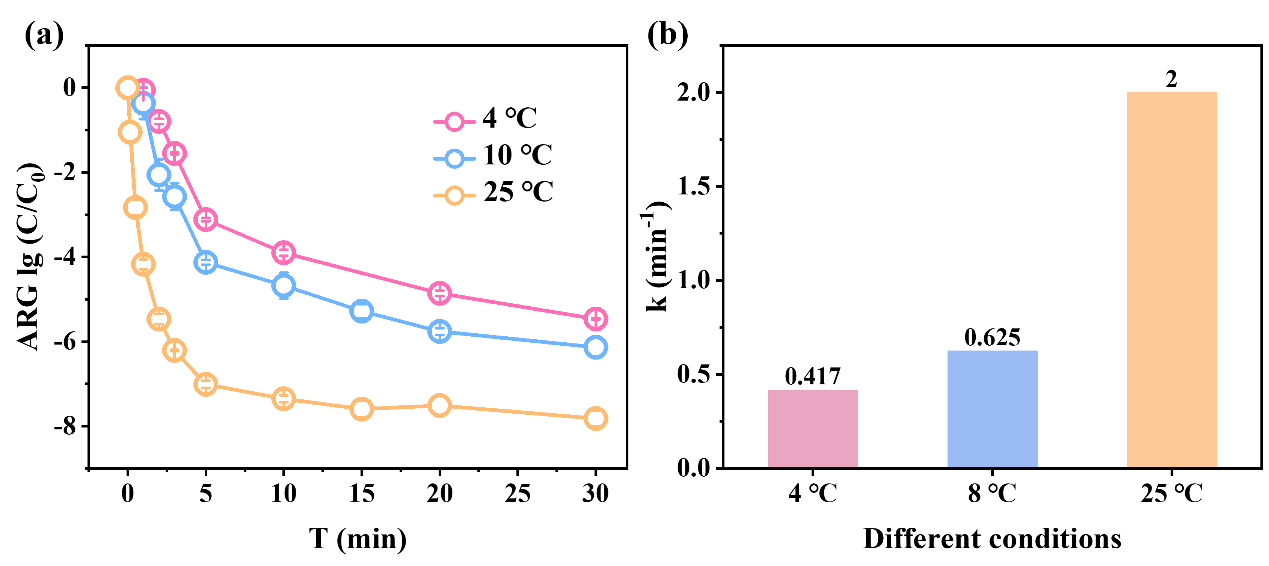


**Figure S45.** The degradation of *bla*_TEM-1_ by the Co-NC/PMS system after 30 min of reaction at different temperatures. Experimental conditions: [Catalyst] = 100 mg/L, [PMS] = 0.5 mM, [ARG] = 10^12^ copies/mL.


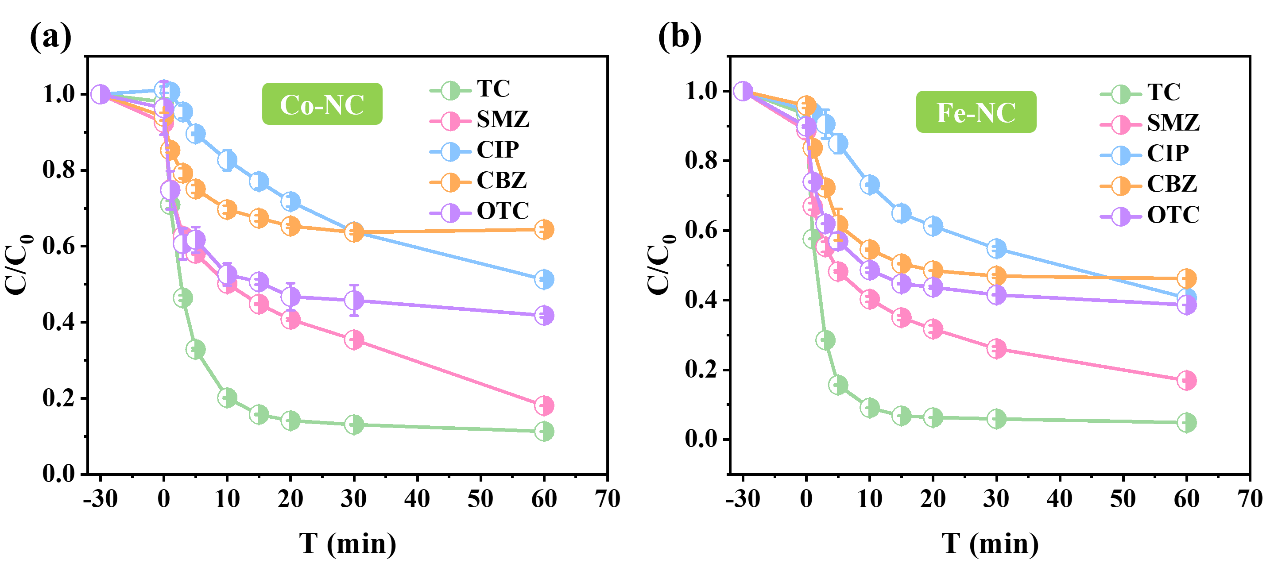


**Figure S46.** Degradation of different antibiotics by Co-NC (a) and Fe-NC (b). Experimental conditions: [Catalyst] = 100 mg/L, [PMS] = 0.5 mM, T = 25 ℃, [Pollutant] = 100 µM.


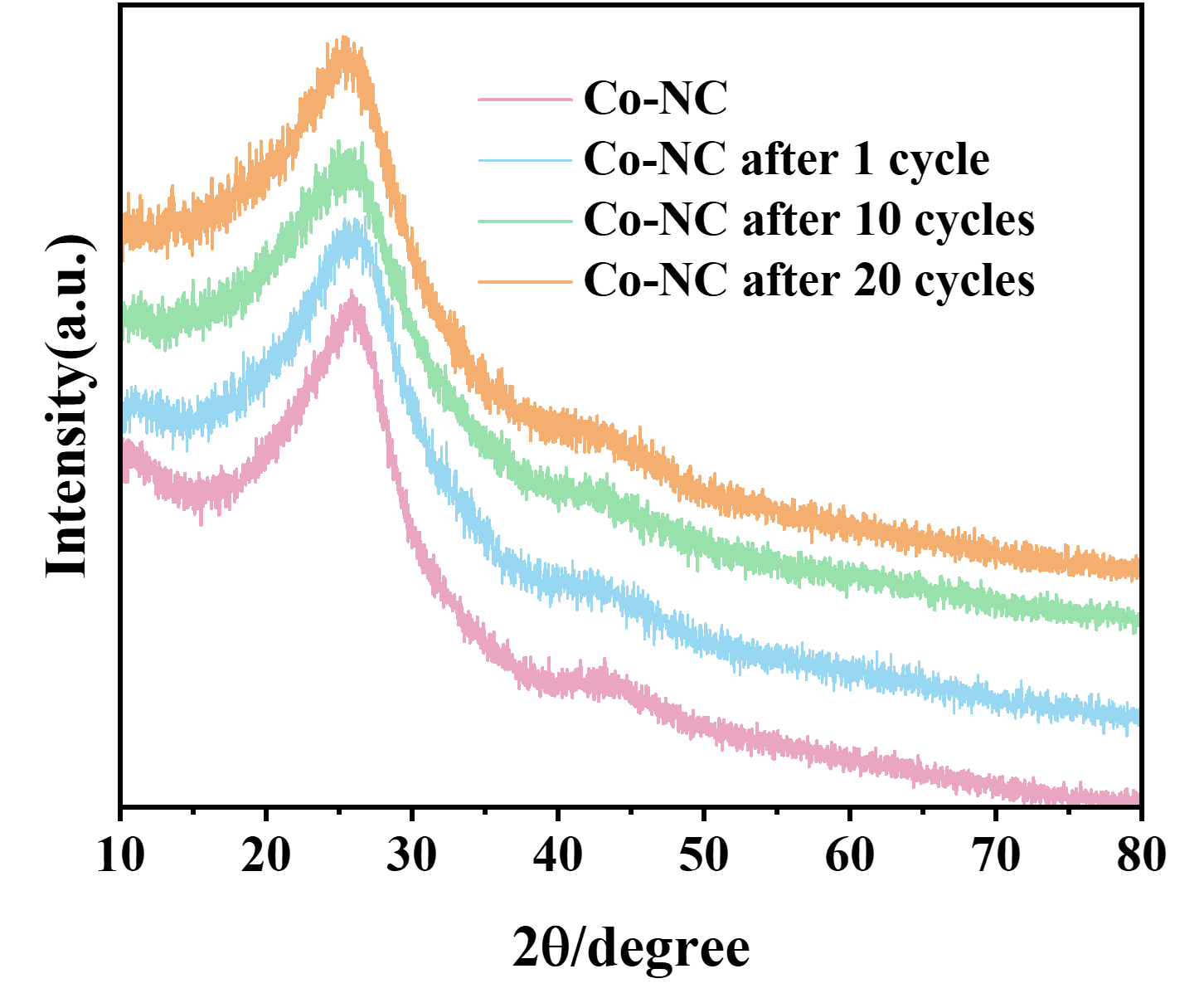


**Figure S47.** XRD patterns of Co-NC after cycling.


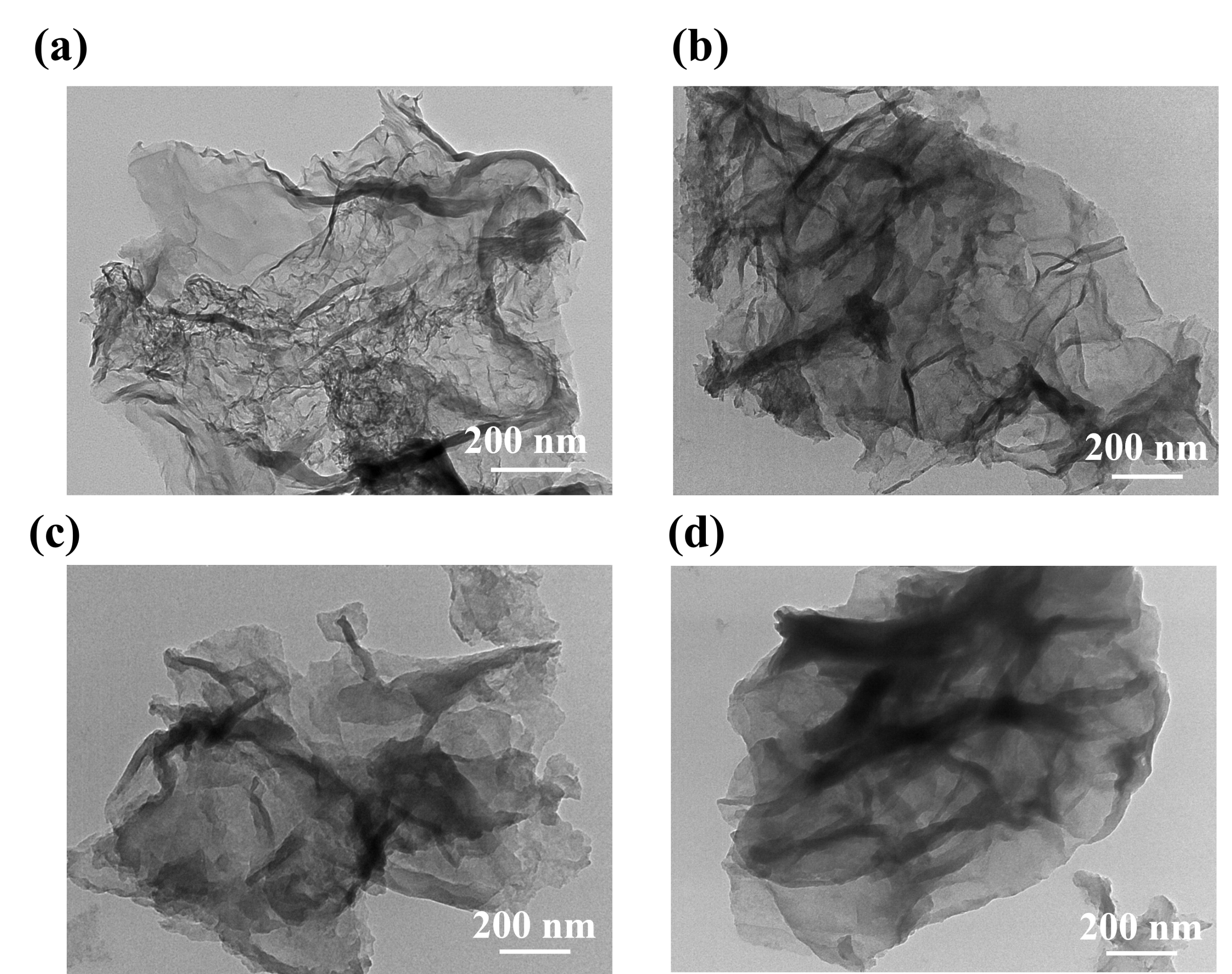


**Figure S48.** TEM images of Co-NC initially (a), after 1 cycle (b) and after 10 cycles (c) and after 20 cycles (d).


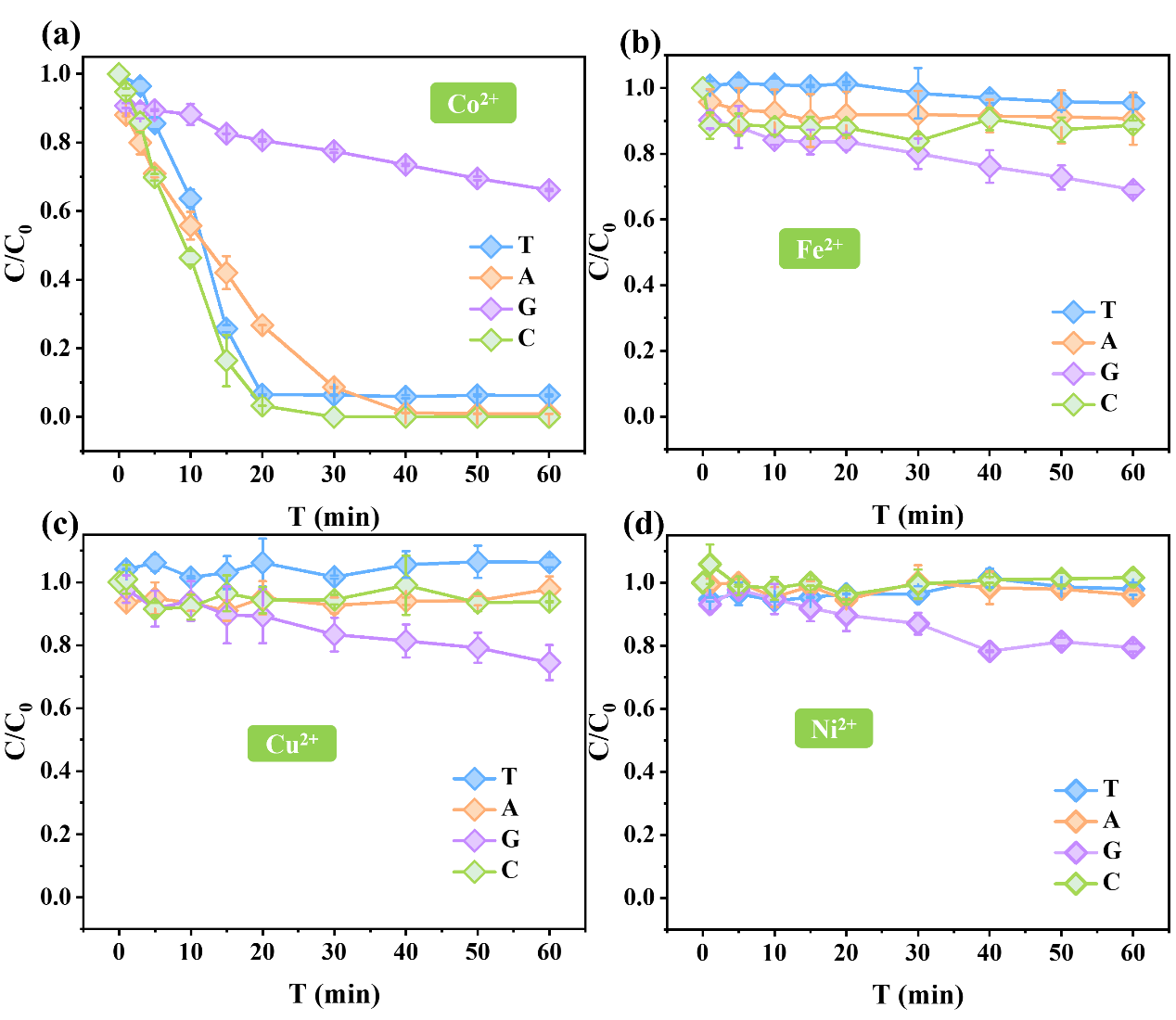


**Figure S49.** (a) Degradation performance of different metal ions to different bases: A, T, G and C. Experimental conditions: [Co^2+^] = 0.7 mg/L, [Fe^2+^] = 0.5 mg/L, [Cu^2+^] = 0.5 mg/L, [Ni^2+^] = 0.5 mg/L, [PMS] = 0.5 mM, T = 25 ºC, [A] = [T] = [C] = [G] = 50 µM.


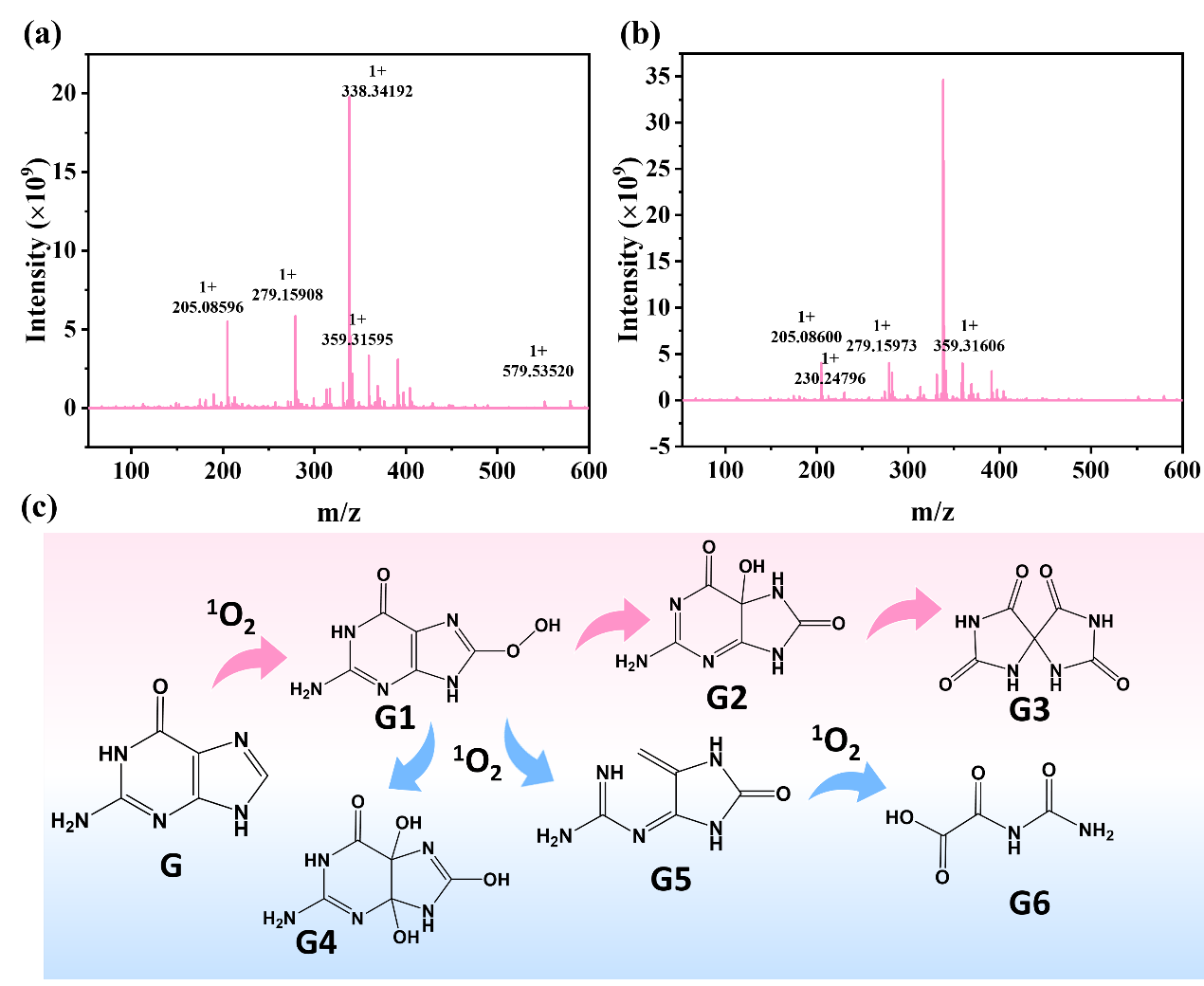


**Figure S50.** (a-b) Ultra-high-resolution mass spectra during the G degradation in Co-NC/PMS reaction system. (c) Proposed degradation pathways of G in Co-NC system. Conditions: [G] = 100 µM, [Catalyst] = 100 mg/L, [PMS] = 0.5 mM, T = 25 ºC.


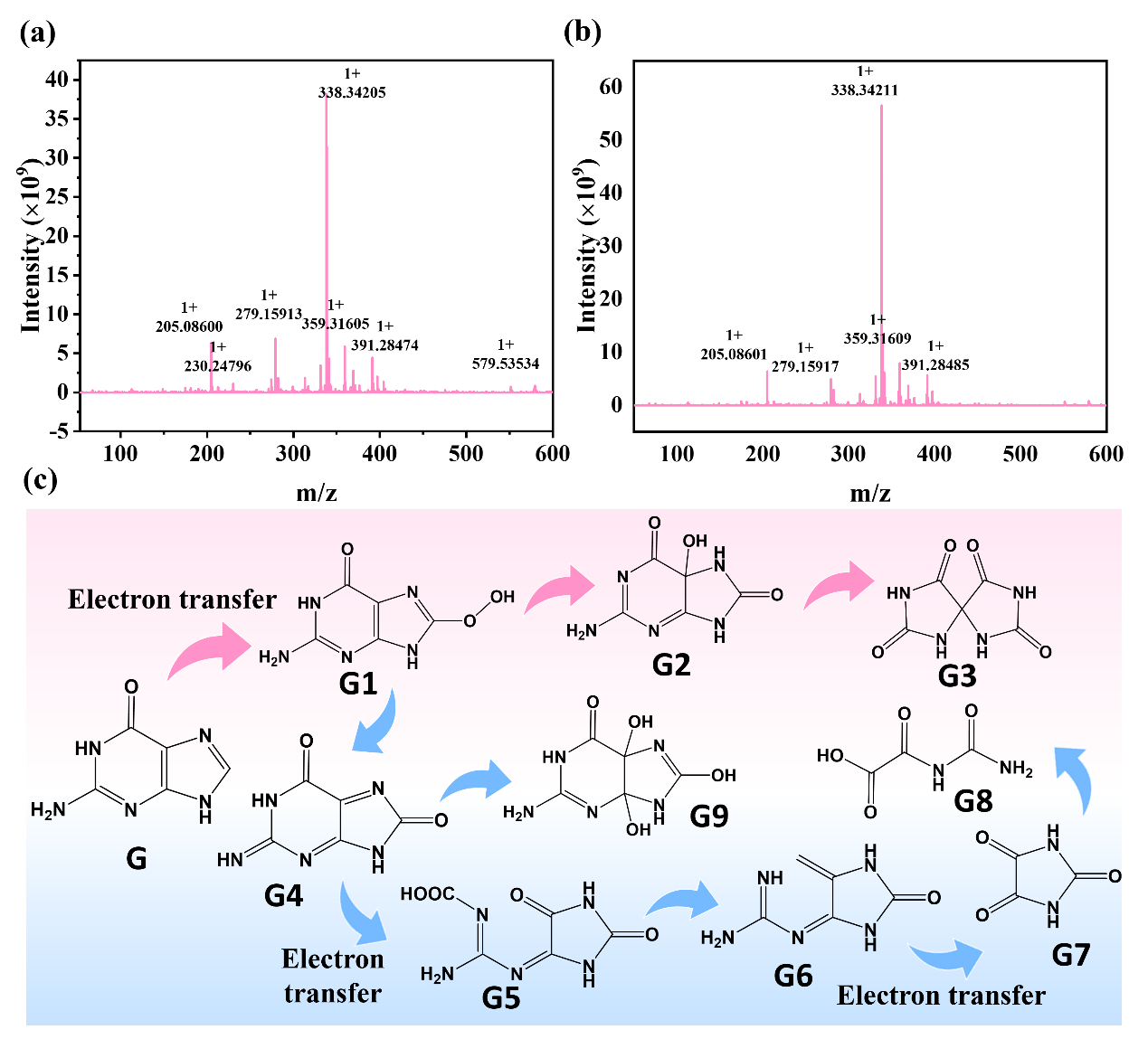


**Figure S51.** (a-b) Ultra-high-resolution mass spectra during the G degradation in Fe-NC/PMS reaction system. (c) Proposed degradation pathways of G in Fe-NC system. Conditions: [G] = 100 µM, [Catalyst] = 100 mg/L, [PMS] = 0.5 mM, T = 25 ºC.

The Co-NC and Fe-NC degradation pathway is based on the peak analysis in the LC-MS chromatogram. Other undetected reaction intermediates may also exist.


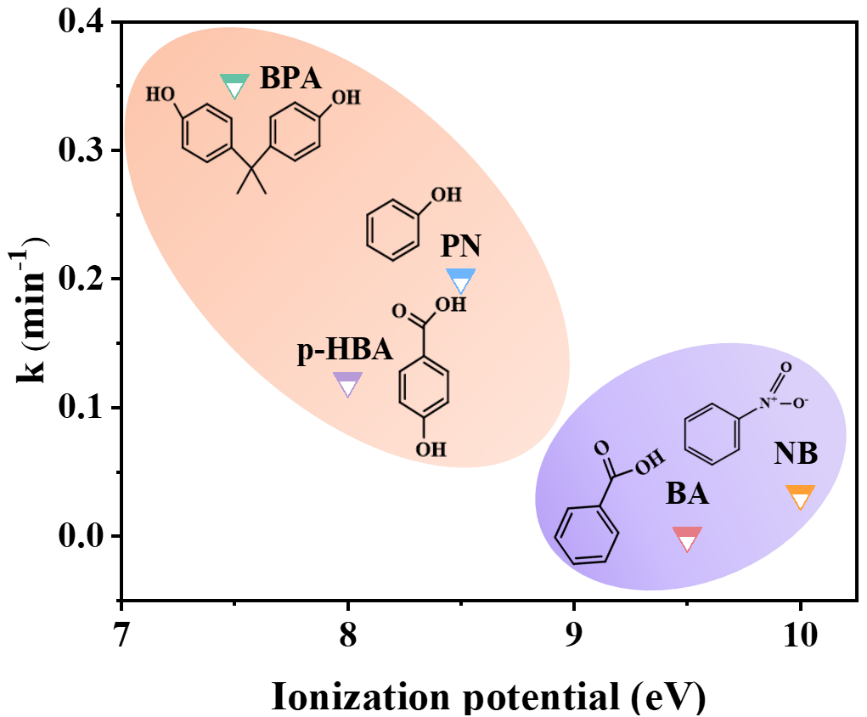


**Figure S52.** The relationship between degradation rate constant and ionization potential. Experimental conditions: [Catalyst] = 100 mg/L, [PMS] = 0.5 mM, [Pollutant] = 50 µM, T = 25 ºC.

**Table S1. The proportion of different kinds of C, N, and O in different single atoms catalysts**

| Element | | Composition | Proportion of different compositions | | | |
| --- | --- | --- | --- | --- | --- | --- |
|  |  |  | Co-NC | Fe-NC | Cu-NC | Ni-NC |
| C | C=O | | 50.32% | 45.73% | 51.20% | 54.61% |
|  | C=N | | 36.74% | 40.54% | 29.78% | 33.21% |
|  | C-N | | 12.94% | 13.73% | 19.02% | 12.18% |
| N | Pyridinic N | | 28.27% | 24.93% | 13.26% | 22.58% |
|  | Pyrrolic N | | 18.49% | 20.09% | 15.65% | 22.02% |
|  | Graphitic N | | 53.26% | 44.43% | 52.52% | 42.73% |
|  | Oxidized N | | 9.38% | 10.55% | 18.57% | 12.67% |
| O | C=O | | 38.96% | 38.18% | 35.07% | 17.24% |
|  | C-O | | 38.31% | 37.58% | 32.54% | 48.28% |
|  | OH | | 22.73% | 24.24% | 32.37% | 34.48% |

**New Table S2.** **The normalized kinetic comparison for G degradation in different reaction system**

| Catalyst^a^ | Catalyst dose (mg/L) | PMS Concentration (mg/L) | G removal  (μM) | Time  (min) | Degradation rate constant *k* (min^-1^) | Normalized activity evaluation parameters | References |
| --- | --- | --- | --- | --- | --- | --- | --- |
| MF/PMS | 200 | 200 | 10 | 5 | 0.29 | 0.071 | 19 |
| Co-CVO/PMS | 200 | 200 | 10 | 60 | 0.013 | 0.0030 | 4 |
| Co@NBC700/PMS | 30 | 123 | 10 | 4 | 0.96 | 2.60 | 20 |
| CoMn@NC)/PMS | 300 | 308 | 10 | 5 | 0.46 | 0.050 | 21 |
| [PBA@C-1/PMS](mailto:PBA@C-1/PMS) | 100 | 300 | 50 | 30 | 0.086 | 0.14 | 22 |
| Fe-NC/PMS | 100 | 308 | 50 | 3 | 1.94 | 3.15 | This work |
| Co-NC/PMS | 100 | 308 | 50 | 10 | 0.93 | 1.51 | This work |

^a^MF: MoS_2_@Fe_3_O_4_; CVO: CeVO_4_; NBC: N-doped biochar; NC: N-doped composites; PBA: Prussian blue analogues

**Table S3. EXAFS fitting parameters at the Co K-edge for various samples.**

| Sample | | Shell | CN^a^ | R(Å)^b^ | σ^2^(Å^2^)^c^ | ΔE_0_(eV)^d^ | | K-range/Å^-1^ | R-range/Å | R factor |
| --- | --- | --- | --- | --- | --- | --- | --- | --- | --- | --- |
| Co foil | Co-Co | | 12* | 2.50±0.01 | 0.0065±0.0004 | 8.5±0.2 | 3.0-12.0 | | 1.0-3.0 | 0.0027 |
| CoPc | Co-N | | 4.0±0.3 | 1.90±0.01 | 0.0029±0.0007 | 5.8±0.6 | 2.2-13.5 | | 1.1-3.2 | 0.0096 |
|  | Co-C | | 7.9±2.1 | 2.90±0.01 | 0.0054±0.0026 | 1.3±0.9 |  |  |  |  |
|  | Co-N | | 3.2±1.5 | 3.26±0.01 | 0.0018±0.0036 | 3.5±1.7 |  |  |  |  |
| Co_3_O_4_ | Co-O | | 4.0±0.2 | 1.92±0.01 | 0.0021±0.0006 | -0.1±0.4 | 3.0-12.0 | | 1.0-3.5 | 0.0021 |
|  | Co-Co | | 3.8±0.4 | 2.85±0.01 | 0.0037±0.0007 | -1.8±0.5 |  |  |  |  |
|  | Co-Co | | 8.1±0.9 | 3.36±0.01 | 0.0070±0.0009 | -2.9±0.4 |  |  |  |  |
| Co-NC | Co-N | | 2.3±0.3 | 1.90±0.01 | 0.0071±0.0018 | -1.0±1.1 | 2.6-12.0 | | 1.0-2.8 | 0.0039 |
|  | Co-C | | 1.8±0.3 | 2.10±0.01 |  |  |  |  |  |  |

*^a^CN*, coordination number; *^b^R*, the distance to the neighboring atom; *^c^σ*^2^, Debye-Waller factor, the Mean Square Relative Displacement (MSRD); *^d^ΔE*_0_, inner potential correction; *R* factor indicates the goodness of the fit. *S*0^2^ was fixed to 0.800, according to the experimental EXAFS fit of Co foil by fixing *CN* as the known crystallographic value. * This value was fixed during EXAFS fitting, based on the known structure of Co. Error bounds that characterize the structural parameters obtained by EXAFS spectroscopy were estimated as CN ± 20%; R ± 1%; σ^2^ ± 20%; ΔE_0_ ± 20%. A reasonable range of EXAFS fitting parameters: 0.700 < *Ѕ*_0_^2^ < 1.000; *CN >* 0; *σ*^2^ > 0 Å^2^; |Δ*E*_0_| < 15 eV; *R* factor < 0.02.

**Table S4.** **Fukui function values of GA and GC**

**Table S5. Fukui function values of GG and GT**

**Table S6. Relative parameters of different pollutants.**

|  | Mulliken  electronegativity  (eV) | Chemical  potential   (eV) | Hard-ness  (eV) | Soft-ness  (eV^-1^) | Electrophilicity  index (eV) | Nucleophilicity  index (eV) | HOMO (eV) | LUMO  (eV) |
| --- | --- | --- | --- | --- | --- | --- | --- | --- |
| TC | 4.29 | -4.29 | 6.81 | 0.15 | 1.35 | 3.21 | -6.25 | -2.35 |
| BPA | 3.61 | -3.61 | 7.72 | 0.13 | 0.84 | 3.5 | -5.96 | -0.62 |
| CBZ | 4.04 | -4.04 | 7.47 | 0.13 | 1.09 | 3.22 | -6.24 | -1.79 |
| CIP | 4.38 | -4.38 | 7.29 | 0.14 | 1.31 | 2.89 | -6.57 | -2.16 |
| SMZ | 4.13 | -4.13 | 8.17 | 0.12 | 1.04 | 2.79 | -6.67 | -1.43 |
| NB | 5.54 | -5.54 | 9.06 | 0.11 | 1.7 | 1.46 | -7.99 | -3.04 |

**Table S7. HPLC for each pollutant**

| Pollutant | Flow velocity  (mL/min) | Mobile phase A: B | Volume ratio (V: V) | Detection wavelength  (nm) |
| --- | --- | --- | --- | --- |
| A | 1 | 1% formic acid water: methanol | 95:5 | 266 |
| T | 1 | 1% formic acid water: methanol | 95:5 | 266 |
| C | 0.7 | 1% formic acid water: methanol | 70:30 | 266 |
| G | 1 | 1% formic acid water: methanol | 95:5 | 266 |
| PN | 0.8 | 1% formic acid water: methanol | 30:70 | 270 |
| BPA | 0.8 | water: methanol | 30:70 | 280 |
| PN | 0.8 | 1% formic acid water: methanol | 30:70 | 270 |
| BPA | 0.8 | water: methanol | 30:70 | 280 |
| BA | 1 | 1% formic acid water: methanol | 40:60 | 270 |
| NB | 1 | water: acetonitrile | 30:70 | 270 |
| FFA | 1 | water: methanol | 50:50 | 219 |
| p-CBA | 1 | 1% formic acid water: acetonitrile | 40:60 | 239 |
| TC | 0.8 | 1% formic acid water: methanol | 40:60 | 375 |
| SMZ | 1 | 1% formic acid water: acetonitrile | 60:40 | 275 |
| CIP | 1 | 1% formic acid water: acetonitrile | 40:60 | 278 |
| CBZ | 1 | water: methanol | 40:60 | 240 |

**Table S8. The information of primers and amplicons for qPCR analysis.**

| ARGs | Prime sequence | Amplicon length | Amplification efficiency |
| --- | --- | --- | --- |
| *bla*_TEM-1_ | F: AATAAACCAGCCAGCCGGAA | 209 bp | 105.60% |
|  | R: TTGATCGTTGGGAACCGGAG |  |  |
| *tet*A | F: GACTATCGTCGCCGCACTTA | 216 bp | 101.80% |
|  | R: ATAATGGCCTGCTTCTCGCC |  |  |
| *ampR* | F: GGCTCATCCTTCGGTGTGAA | 192 bp | 105.70% |
|  | R: TTGGAAAACGTTCTTCGGGG |  |  |
| *tet*M | F: AGCTCATGTTGATGCAGGAA | 97 bp | 103.70% |
|  | R: CGTTGTACCTTTGTCCACGC |  |  |
| *cat* | F: CATCAGGCGGGCAAGAATGTG | 119 bp | 106.30% |
|  | R: TTGCTCAATGTACCTATAACCAGACC |  |  |
| *kan*A | F: CATTCGTGATTGCGCCTGAG | 168 bp | 101.20% |
|  | R: ATCCCCGGGAAAACAGCATT |  |  |
| *van*A | F: GGCTCATCCTTCGGTGTGAA | 171 bp | 99.70% |
|  | R: GTCCACCTCGCCAACAACTA |  |  |

**Table S9. The information related to ion chromatography detection**

|  | Retention time (min) | Peak name | Peak area (µS*min) | Peak height (µS) | Sample size (ppm) |
| --- | --- | --- | --- | --- | --- |
| Co-NC | 6.61 | NO_3_^-^ | 0.05 | 0.31 | 0.88 |
| Fe-NC | 6.62 | NO_3_^-^ | 0.05 | 0.32 | 0.89 |

**Table S10. The comparison of the efficiency of different AOPs for ARG degradation.**

| Catalyst^a^ | ARGs | Catalyst  dose | PMS  dose | ARG removal  (lg C_0_/C copies/mL) | Time | Degradation rate constant k (min^-1^) | Ref |
| --- | --- | --- | --- | --- | --- | --- | --- |
| Co_SA_/Ti_3_C_2_T_x_/PMS | *tet*A | 200 mg/L | 200 mg/L | 7 | 20 min | 0.94 | 7 |
| MFAs-0.1/PMS | *sul1* and *AmpR* | 250 mg/L | 500 mg/L | 5.47 | 80 min | 0.161 | 8 |
| S-nZVI/PMS | *sul1*, *intI1 and tet*A | 28 mg/L | 0.5 mM | 1.52, 1.79, 1.56 | 25 min | 0.14 | 9 |
| CN-CV_2_/PS | *tetA*, *intI*1 | 1000 mg/L | 2 mM | 2.5 | 180 min | 0.032 | 10 |
| TiO_2_-CuPd | *bla*_TEM_*, ermB, tetM, intl1* | 200 mg/L |  | More than 2 | 120 min | 0.0384 | 11 |
| Co_3_O_4_-SiO_2_/PMS | *intI1*, *sul1* | 100 mg/L | 20 mg/L | 7.43, 7.36 | 60 min | 0.17 | 12 |
| UVA/MIP-C_3_N_4_ | *bla*_NDM-1_ | 500 mg/L |  | 6.33 | 30 min | 0.111 | 13 |
| UVA/NGWM | *bla*_NDM_ | 200 mg/L |  | 8 | 120 min | 0.042 | 14 |
| Fe-Co@BC/PMS | *bla*_TEM-1_ | 80 mg/L | 1 mM | 6.47 | 60 min | 0.058 | 15 |
| FeN-RBC/PMS | *tet*B | 50 mg/L | 1 mM | 6 | 30 min | 0.1 | 16 |
| C/O-g-C_3_N_4_/PDS | *tet*B | 100 mg/L | 6 mM | 5 | 40 min | 0.125 | 17 |
| Co-CVO/PMS | *tet*A | 200 mg/L | 200 mg/L | 5.4 | 15 min | 1.241 | 6 |
| S-nZVI/PS | *bla*_TEM-1_ *tet*A | 100 mg/L | 1 mM | 8 | 5 | 1.6 | 18 |
| nZVI/PS | *bla*_TEM-1_ *tet*A | 100 mg/L | 1 mM | 8 | 5 | 1.2 | 18 |

^a^Co_SA_/Ti_3_C_2_T_x_: Single cobalt atoms immobilized on Ti_3_C_2_T_x_ Mxene; MFAs: Mo-doped FeS_2_ nanosheets grown on activated carbon fibers; S-nZVI: Sulfidated nanoscale zerovalent iron; CN-CV_2_: C-(N)_3_ defect carbon; UVA: Ultraviolet A; MIP: Molecularly-imprinted; NGWM: nitrogen-doped reduced graphene oxide-wrapped hierarchical Bi_2_O_2_CO_3_ microspheres; BC: Biochar; FeN-RBC: Iron and nitrogen-doped biochar derived from rice straw.

**Table S11. The intermediate products formed during the degradation of base G in Co-NC/PMS reaction system.**

| Compounds | m/z | Tentative structure |
| --- | --- | --- |
| G | 151.0494 |  |
| G1 | 183.0392 |  |
| G2 | 183.0392 |  |
| G3 | 183.0392 |  |
| G4 | 201.0498 | 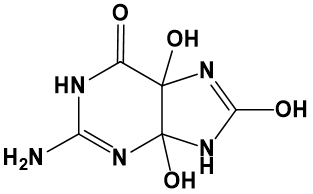 |
| G5 | 155.0443 |  |
| G6 | 132.0171 |  |

**Table S12. The intermediate products formed during the degradation of base G in Fe-NC/PMS system.**

| Compounds | m/z | Tentative structure |
| --- | --- | --- |
| G | 151.0494 |  |
| G1 | 183.0392 |  |
| G2 | 183.0392 |  |
| G3 | 183.0392 |  |
| G4 | 165.0287 |  |
| G5 | 199.0342 | \|  \| \| --- \| |
| G6 | 155.0443 |  |
| G7 | 114.0065 |  |
| G8 | 132.0171 |  |
| G9 | 201.0498 | 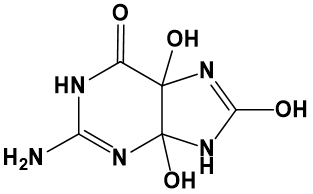 |

**References for SI only**

[1] G. F. de Lima, H. A. Duarte, L. G. M. Pettersson, X-ray absorption near-Edge spectroscopy calculations on pristine and modified chalcopyrite surfaces. *J. Phys. Chem. C*. **2018,** 122(35), 20200–20200

[2] C. Xu, R. Schierac, C. Cocchi, Ab initio X-ray near-edge spectroscopy of sodium-based multi-alkali antimonides. *Phys. Chem. Chem. Phys*. **2025,** 27, 19071-19080.

[3] J. Z. Zhen, J. H. Sun, X. W. Xu Z. L Wu, W. K. Song, Y. Z. Ying, S. K. Liang, L. S. Miao, J. Z. Cao, W. Y. Lv, C. S. Song, Y. Y. Yao, M. Y. Xing, M−N_3_ configuration on boron nitride boosts singlet oxygen generation via peroxymonosulfate activation for selective oxidation. *Angew. Chem. Int. Ed.* **2024,** 63(26), e202402669.

[4] Y. H. Long, Z. H. Cao, W. R. Wu, W. H. Liu, P. Z. Yang, X. S. Zhan, R. Z. Chen, D. F. Liu, W. L. Huang, Rational modulation of Fe single-atom electronic structure in a Fe-N_2_B_4_ configuration for preferential ^1^O_2_ generation in Fenton-like reactions. *Appl. Catal. B* **2024,** 344, 123643.

[5] Y. Y. Wang, B. Ma, J. Zhao, Z. Y. Tang, W. X. Li, C. He, D. H. Xia, K. G. Linden, R. Yin, Rapid inactivation of fungal spores in drinking water by far-UVC photolysis of free chlorine. *Environ. Sci. Technol.* **2023,** 57(51), 21876-21887.

[6] F. Li, P. F. Wang, T. Zhang, M. M. Li, S. Yue, S. H. Zhan, Y. Li, Efficient removal of antibiotic resistance genes through 4f-2p-3d gradient orbital coupling mediated Fenton-like redox processes. *Angew. Chem. Int. Ed.* **2023,** 62(47), e202313298.

[7] M. M. Li, P. F. Wang, K. D. Zhang, H. X. Zhang, Y. P. Bao, Y. Li, S. H. Zhan, J. C. Crittenden, Single cobalt atoms anchored on Ti_3_C_2_T_x_ with dual reaction sites for efficient adsorption-degradation of antibiotic resistance genes. *Proc. Natl. Acad. Sci. U.S.A.* **2023,** 120(29), e2305705120.

[8] C. C. Yan, J. Li, Z. H. Sun, L. Y. Chen, X. Sun, X. J. Wang, S. Q. Xia, Engineering sulfur vacancies on Mo-doped FeS_2_ nanosheets grown on activated carbon fibers enhances peroxymonosulfate activation for efficient elimination of sulfamethazine, antibiotic resistant bacteria and antibiotic resistance genes: The dominant role of singlet oxygen. *Chem. Eng. J.* **2024,** 493, 152643.

[9] Y. Liu, J. F. Gao, Y. W. Wang, W. J. Duan, J. Liu, Y. Zhang, H. R. Zhang, M. Y. Zhao, The removal of antibiotic resistant bacteria and genes and inhibition of the horizontal gene transfer by contrastive research on sulfidated nanoscale zerovalent iron activating peroxymonosulfate or peroxydisulfate. *J. Hazard. Mater.* **2022,** 423, 126866.

[10] B. R. Gao, M. M. Dou, J. Wang, S. M. Li, D. Y. Wang, L. Ci, Y. Fu, Efficient persulfate activation by carbon defects g-C_3_N_4_ containing electron traps for the removal of antibiotics, resistant bacteria and genes. *Chem. Eng. J.* **2021,** 426, 131677.

[11] M. Gmurek, J. Alexander, P. Mazierski, M. Miody, M. Fronczak, T. Klimczuk, Zaleska- A. Medynska, H. Horn, T. Schwartz, Enhancement of photocatalytic-based processes by mono- and bimetallic (CuPd) rutile loaded nanoparticles for antibiotic resistance genes and facultative pathogenic bacteria removal. *Chem. Eng. J.* **2023,** 462, 142243.

[12] Y. H. Tian, S. J. Yao, L. Zhou, Y. R. Hu, J. Y. Lei, L. Z. Wang, J. L. Zhang, Y. D. Liu, C. Z. Cui, Efficient removal of antibiotic-resistant bacteria and intracellular antibiotic resistance genes by heterogeneous activation of peroxymonosulfate on hierarchical macro-mesoporous Co_3_O_4_-SiO_2_ with enhanced photogenerated charges. *J. Hazard. Mater.* **2022,** 430, 127414.

[13] Q. B. Yuan, D. N. Zhang, P. F. Yu, R. N. Sun, H. Javed, G. Wu, P. J. J. Alvarez, Selective adsorption and photocatalytic degradation of extracellular antibiotic resistance genes by molecularly-imprinted graphitic carbon nitride. *Environ. Sci. Technol.* **2020,** 54(7), 4621-4630.

[14] D. Y. Li, P. F. Yu, X. F. Zhou, J. H. Kim, Y. L. Zhang, P. J. J. Alvarez, Hierarchical Bi_2_O_2_CO_3_ wrapped with modified graphene oxide for adsorption-enhanced photocatalytic inactivation of antibiotic resistant bacteria and resistance genes. *Water Res.* **2020,** 184, 116157.

[15] L.; An, J. Wang, J. Wang, H. R. Liu, F. Wu, T. Hu, X. Qian, L. Zhang, Y. F. Sun, X. J. Wang, J. Gu, Efficient peroxymonosulfate activation by iron-cobalt bimetallic biochar for rapid removal of antibiotic resistant bacteria, antibiotic resistance genes, and ampicillin: The coexisting of free-radical and non-radical pathways. *Sep. Purif. Technol.* **2024,** 342, 127025.

[16] D. L. Huang, H. Huang, G. F. Wang, R. J. Li, R. H. Xiao, L. Du, W. Zhou, W. B. Xu, Simultaneous elimination of antibiotic-resistant bacteria and antibiotic resistance genes by different Fe-N Co-doped biochars activating peroxymonosulfate: The key role of pyridine-N and Fe-N sites. *J. Colloid Interface Sci.* **2024,** 668, 12-24.

[17] J. E. Du, N. Zhang, S. L. Ma, G. S. Wang, C. Ma, G. Y. Liu, Y. Wang, J. Z. Wang, T. J. Ni, Z. An, W. D. Wu, Visible light-driven C/O-g-C_3_N_4_ activating peroxydisulfate to effectively inactivate antibiotic resistant bacteria and inhibit the transformation of antibiotic resistance genes: Insights on the mechanism. *J. Hazard. Mater.* **2024,** 464, 132972.

[18] Z. G. Yu, H. Rabiee, J. H. Guo, Synergistic effect of sulfidated nano zerovalent iron and persulfate on inactivating antibiotic resistant bacteria and antibiotic resistance genes. *Water Res.* **2021,** 198, 117141.

[19] Y. N. Chen, Y. H. Hou, F. Y. Liu, Q. Q. Dong, Z. M. Li, P. Han, M. P. Tong, Efficient peroxymonosulfate activation by magnetic MoS_2_@Fe_3_O_4_ for rapid degradation of free DNA bases and antibiotic resistance genes. *Water Res.***2023,** 239, 120026.

[20] H. Huang, L. Shen, D. L. Huang, G. F. Wang, R. J. Li, L. Du, W. B. Xu, W. Zhou, H. J. Chen, T. Tong, Y. Lei, S. B. Liu, Peroxymonosulfate activation by Co-doped chlorella biochar for destructing antibiotic resistance: Targeting the degradation of free DNA bases and inactivation of resistant bacteria. *J. Hazard. Mater.* **2023,** 503, 141058.

[21] H. Huang, D. L. Huang, G. F. Wang, R. J. Li, L. Du, W. B. Xu, H. J. Chen, W. Zhou, R. H. Xiao, L. Shen, Y. Lei, Contaminants modulated the dominant ROS and reaction pathways: Rapid degradation for free DNA bases and antibiotic resistance genes in fenton-like process. *Chem. Eng. J*. **2025**, 511, 162008.

[22] Z. Y. Tong, Q. Li, C. Gao, W. J. Qi, Z. F. Wan, L. Zhang, B. F. Zhang, Modulated oxidation pathways enabled by Co-Fe bimetallic alloy catalysts for effective elimination of antibiotics. *J. Colloid Interface Sci.* **2026**, 707,139680.
